# Effect of the time of day for vaccination on the immune response to Ebola Virus Disease vaccines: A modeling study from PREVAC randomized trial

**DOI:** 10.1371/journal.pntd.0013950

**Published:** 2026-01-30

**Authors:** Ange-Marie D. Kpetigo, Marie Alexandre, Aboubacar Camara, Abdoul Beavogui, Seydou Doumbia, Mark Kieh, Bailah Leigh, Samba Sow, Linda Wittkop, Anne-Aygline Soutthiphong, Irina Maljkovic Berry, Suzanne Fleck, Pauline Akoo, Benjamin Hamze, Deborah Watson-Jones, Jens H. Kuhn, Brian Greenwood, Laura Richert, Yazdan Yazdanpanah, Yves Lévy, Rodolphe Thiébaut, Mélanie Prague, PREVAC Study Team, Edouard Lhomme

**Affiliations:** 1 Bordeaux Population Health Research Centre, Université de Bordeaux, Inserm, and Inria, Bordeaux, France; 2 Vaccine Research Institute, Université Paris-Est Créteil, Faculté de Médicine, Créteil, France; 3 Inserm, Paris, France; 4 Centre National de Formation et de Recherche en Santé Rurale (CNFRSR) de Mafèrinyah, Ministère de la Santé et de l’Hygiène Publique, Mafèrinyah, Guinea; 5 University Clinical Research Center (UCRC), University of Sciences, Technique and Technology of Bamako (USTTB), Bamako, Mali; 6 Partnership for Research on Ebola Virus in Liberia (PREVAIL), Monrovia, Liberia; 7 College of Medicine and Allied Health Sciences (COMAHS), University of Sierra Leone, Tower Hill, Freetown, Sierra Leone; 8 Centre pour le Développement des Vaccins, Ministère de la Santé et du Développement Social du Mali, Bamako, Mali; 9 EUropean CLInical Trials Platform & Development (EUCLID), Centre Hospitalier Universitaire Bordeaux, Université de Bordeaux, and Inserm, Bordeaux, France; 10 Pôle de Santé Publique, Service d’Information Médicale, Centre Hospitalier Universitaire Bordeaux, Bordeaux, France; 11 UMS 54 Methods and Applied Research for Trials (UMS 54 MART), Université de Bordeaux, and Inserm, Bordeaux, France; 12 Integrated Research Facility at Fort Detrick, National Institute of Allergy and Infectious Diseases, National Institutes of Health (NIH), Fort Detrick, Frederick, Maryland, United States of America; 13 London School of Hygiene & Tropical Medicine (LSHTM), London, United Kingdom; 14 French Agency for Research on AIDS and Viral Hepatitis (ANRS), Emerging Infectious Diseases, PariSanté Campus, Paris, France; 15 Service de Maladies Infectieuses et Tropicales, Hôpital Bichat, Paris, France; 16 Service d’Immunologie Clinique et Maladies Infectieuses, Hôpital Henri-Mondor, Créteil, France; University of California, Los Angeles, UNITED STATES OF AMERICA

## Abstract

**Background:**

Emerging evidence suggests that the time in the day of vaccination may influence post-vaccination immunogenicity. The main objective of this study was to assess the association between the time of vaccination and the anti-EBOV GP_1,2_ IgG antibody response at 12 mo following vaccination against Ebola virus disease (EVD).

**Methodology/Principal Findings:**

This study utilized data from a randomized, double-blind, placebo-controlled international phase 2b clinical trial (PREVAC) evaluating the immunogenicity of three vaccination strategies against Ebola virus disease (rVSVΔG-ZEBOV-GP one and two doses, and Ad26.ZEBOV/MVA-BN-Filo) in 1,859 healthy Western Africans. In the overall population, we measured a statistically significant association between the time of day of first vaccination and anti-Ebola virus immunoglobulin G levels at 12 mo (*p* = 0.02). The magnitude of this association was small, participants vaccinated at 1600 h were estimated to have 1–7% lower antibody levels at 12 mo compared to those vaccinated at 1000 h.

**Conclusions/Significance:**

An effect of the time of first vaccination on the antibody responses was found but remains modest and unlikely to impact the EVD vaccine effectiveness.

**Clinical Trials Registration:**

ClinicalTrials.gov registration: NCT02876328.

## Author summary

Vaccines are one of the most effective ways to prevent severe infectious diseases, but many factors can influence how well they work. One question that has received little attention is whether the time of day when a vaccine is given affects the immune response. In our study, we explored this idea in the context of vaccines against Ebola virus disease (EVD). We analyzed data from a large international clinical trial carried out in Western Africa, where different EVD vaccines were tested. We compared people who received their first vaccine in the morning with those who were vaccinated later in the afternoon. We found that the timing of vaccination was linked to small differences in antibody levels one year later. Participants vaccinated in the afternoon had slightly lower levels of antibodies than those vaccinated in the morning. However, this difference was modest and unlikely to change how well the vaccines protect against EVD. Our results suggest that the time of day may have a minor effect on how the immune system responds to vaccines, but it does not reduce the overall effectiveness of EVD vaccines.

## Introduction

Since its discovery in 1976, Ebola virus (EBOV; species *Orthoebolavirus zairense*; family *Filoviridae*) has caused frequent but unpredictable outbreaks of Ebola virus disease (EVD), primarily affecting Equatorial Africa [1]. Two vaccines—rVSVΔG-ZEBOV-GP (based on a recombinant vesicular stomatitis Indiana virus with an EBOV glycoprotein[GP_1,2_]) and AD26.ZEBOV, MVA-BN-Filo (dose one based on a recombinant human adenovirus 26 encoding EBOV GP_1,2_, followed by dose two of based on modified vaccinia virus Ankara [MVA] Bavarian Nordic strain encoding EBOV GP_1,2_ and three other proteins from related viruses)—were prequalified by the World Health Organization (WHO) in 2019 and approved by the European Medicines Agency (EMA) in 2020 [2]. rVSVΔG-ZEBOV-GP was also licensed by the U.S. Food and Drug Administration (FDA) [3]. Both vaccine regimens are highly immunogenic and safe and have been widely used in recent outbreaks [4–7]. Although correlates of protection have not yet been established, levels of immunoglobulin G (IgG) antibodies targeting EBOV GP_1,2_ strongly associate with protective immune responses [8,9]. In clinical development, assessing the ability of a candidate vaccine to elicit such immune responses remains a critical objective.

The immune system is shaped by a complex interplay of extrinsic factors (e.g., environment and geographic location) and intrinsic factors (e.g., age, sex, genetics, and co-infections) [10–14]. Among all factors, the circadian rhythm—the approximately 24-h internal interval aligned with Earth’s rotation and the day–night cycle—plays a critical role in synchronizing physiological processes to adapt to environmental changes, such as light exposure and food intake [15,16]. Circadian rhythm influences the induction of adaptive immunity, as demonstrated by the time-of-day-dependent migration of dendritic cells into the draining lymph nodes, leading to a rhythmic adaptive immune response [17]. Functional molecular clocks have been identified within essential immune cells, including natural killer cells, B cells, dendritic cells, and macrophages, highlighting the intricate link between circadian rhythm and biological activities, particularly immune response regulation [18,19].

Emerging evidence suggests that the time in the day of vaccination can influence recipient immunogenicity and, thus, vaccine efficacy [13,14]. For instance, morning administration has been associated with enhanced immune responses for influenza, tuberculosis, and coronavirus disease 2019 vaccines [20–25]. However, improved responses to vaccinations administered later in the day have also been observed [26,27]. These conflicting findings highlight the complexity of the relationship between circadian rhythm and vaccine efficacy. Additionally, most studies were based on retrospective observational designs, which limit causal inference. Although various statistic models have been used, they often suffer from incomplete adjustment for confounding factors or lack the statistical power needed for more robust analyses [25,28–30].

Given the critical role of vaccine responses in providing protection against EVD, particularly during outbreaks, it is essential to identify and characterize the key determinants of immune responses to vaccination. Several factors, including age, sex, geographic location, and pre-vaccination antibody levels, are associated with post-vaccination immunogenicity for both rVSVΔG-ZEBOV-GP and Ad26.ZEBOV,MVA-BN-Filo [31–34]. However, no data are available on the potential influence of the time in the day of vaccination on the immune response to EVD vaccines. The primary objective of our study was to evaluate the association between the time in the day of vaccination and the level of antibody response (anti-EBOV GP_1,2_ IgG) at 12 mo induced by one dose of rVSVΔG-ZEBOV-GP alone (referred to throughout as rVSV arm), rVSVΔG-ZEBOV-GP plus a second dose of the same vaccine (referred to throughout as rVSV-booster arm), or the Ad26.ZEBOV, MVA-BN-Filo two-dose strategy (referred to throughout as Ad26-MVA arm), based on the data from the large Partnership for Research on Ebola VACcinations (PREVAC) randomized, double-blind, placebo-controlled international phase 2b clinical trial, which included adults and children.

## Methods

### Ethics statement

The study protocol as well as the informed consent and assent forms, including participants’ information materials, were approved by ethics committees of the sponsors (Inserm IRB 00003888, London School of Hygiene and Tropical Medicine [LSHTM], Office of Clinical Research Operations and Regulatory Compliance [OCRPRO], Division of Clinical Research [DCR], National Institute of Allergy and Infectious Diseases [NIAID], and National Institutes of Health [NIH]) and the implementing countries (Guinea, Liberia, Mali, and Sierra Leone) before each version of the protocol was implemented.

All adult participants provided written informed consent. Written informed consent of the parent/guardian was obtained for children of all age groups with written assent if the child was aged 7–17 years old.

### Study design

The PREVAC trial (NCT02876328) was a phase 2b randomized, double-blind, placebo-controlled, international, multicenter clinical trial that assessed vaccination of healthy adults and children older than 1 yr and across four Western African countries in a non-epidemic context [4,35]. The trial aimed to evaluate the immunogenicity and safety of three preventive vaccination strategies against EVD compared to a placebo: (1) one dose of rVSVΔG-ZEBOV-GP, followed by a placebo dose at 56 d (referred to as the rVSV arm); (2) one dose of rVSVΔG-ZEBOV-GP, with a second dose at 56 d (referred to as the rVSV-booster arm); and (3) a dose of Ad26.ZEBOV, followed by a dose of MVA-FN-Filo at 56 d (referred to as the Ad26-MVA arm). The primary endpoint of the trial was the anti-EBOV GP_1,2_ IgG antibody concentrations measured 12 mo after vaccination [4].

### Participants and locations

The participants were adult and pediatric volunteers (>1 yr) from the general population, recruited at six sites: two in Guinea (Landreah, an urban area in Conakry, and Mafèrinyah, a rural area in Forécariah Province); one in Liberia (Redemption Hospital, located in Monrovia, an urban area); two in Mali (the Center for Vaccine Development [CVD] and the University Clinical Research Center [UCRC], both in Bamako, an urban area); and one in Sierra Leone (Mambolo, a rural community in Kambia District, North West Province). The inclusion criteria were previously described [4].

Following randomization, participants received the initial dose of vaccine or placebo on Day 0 and were evaluated at multiple time points. Follow-up visits occurred at 7 (±3), 14 (±3), 28 (±7) d. A second dose of a vaccine or placebo was administered at 56 d window: 53–66 days (-3, + 10 days)). Subsequent follow-up visits were at 63 d (7 ± 3 d after the second dose), and at 3 mo (±14 d), 6 mo (±1 mo), and 12 mo (±1 mo).

Time of vaccination for first and second injections was recorded systematically for all participants in the electronic case report form.

### Antibody assay

At Day 0 prior to vaccination and at each follow-up visit, serum concentrations of IgG-binding antibodies against the EBOV GP_1,2_ glycoprotein were measured using a standardized FANG (Filovirus Animal Non-Clinical Group) ELISA. Results were expressed in ELISA units per milliliter (EU/mL), calculated by interpolating sample optical densities onto a calibrated reference standard curve. The FANG assay was developed and validated to provide harmonized and comparable measurements of anti-EBOV GP_1,2_ IgG responses across vaccine studies and is now widely used as the reference ELISA in filovirus disease vaccine trials [36]. Details and results from the formal validation of the FANG assay were previously described in supplementary section S3.6.1 of the PREVAC trial primary publication [4,35] and S4 Text. Analyses were performed in two laboratories: the Liberian Institute for Biomedical Research (LIBR) in Monrovia, Liberia, processed samples from Sierra Leone and Guinea; the National Institute of Allergy and Infectious Diseases (NIAID) Integrated Research Facility at Fort Detrick (IRF-Frederick) in Maryland, USA, processed samples from Mali and Liberia.

### Statistics

For this ancillary work, the study population consisted of participants who adhered to the treatment protocol. The placebo group was excluded, because its inclusion would not provide relevant contributions to addressing the research question. Briefly, multivariable linear regression models were used to investigate the association between anti-EBOV GP_1,2_ IgG levels and the time of vaccination. In the primary analysis, the dependent variable was the anti-EBOV GP_1,2_ IgG antibody response measured at the 12-mo visit, reflecting the durability of the antibody response post-vaccination. In secondary analyses, the dependent variable used the anti-EBOV GP_1,2_ IgG measured at 28 d (reflecting the peak response after the first vaccination) and at 3 mo (reflecting the peak response after second dose), respectively. These outcomes were log_10_-transformed to meet the normality assumptions required for statistical analysis. The primary explanatory variable was the time of vaccination, modeled as a continuous variable. Separate models were fitted for the time of the first vaccination and for the time of the second vaccination. The adjustment covariates were selected based on a directed acyclic graph (Fig A in S1 Text) informed by prior findings [34]. Factors included age, sex, geographic location, and pre-vaccination anti-EBOV GP_1,2_ IgG antibody levels.

Natural splines with knots at the quintiles were applied to model the quantitative variables, including the time of vaccination, age at data collection, and serum concentration of anti-EBOV GP_1,2_ IgG antibodies at baseline. The selection of five knots was based on the best minimization of the Akaike information criterion for the three nonlinear variables.

Due to the complexity of directly interpreting spline-estimated parameters, we calculated the ratio of antibody concentrations at 12 mo post-vaccination, as predicted by the model, for a given vaccination time *H* relative to a reference time (arbitrarily set at 1000 h) for each participant profile. This reference time was chosen solely for interpretability and does not correspond to a hypothesis-driven comparison or to a peak in the distribution of vaccination times. The primary inference regarding the effect of vaccination timing relied on the overall association captured by the spline model. Bootstrap methods were used to calculate 95% CIs for this ratio at each tested vaccination time point. A total of 1,000 simulated datasets were generated based on the original data. Then, graphs were created to visualize the ratio’s evolution over time, offering a clear representation of the temporal effect on antibody concentration. Analyses were conducted on available data, and missing values were considered missing at random and evenly distributed across the study arms. An effect was considered statistically significant if the *p*-value was less than 0.05 using the Wald test. Subsequently, *p*-values were adjusted for multiple comparisons at each vaccination time using the Benjamini-Hochberg correction method [37]. All analyses were performed with R, version 4.3.2 (R Foundation for Statistical Computing) [38].

## Results

### Study population

Data were from 1,859 participants recruited into the PREVAC trial: 739 to the rVSV arm, 371 to the rVSV-booster arm, and 749 randomized to the Ad26-MVA arm (Fig A in S2 Text). Participant characteristics are summarized in Table 1. The median age (interquartile range [IQR]) across all vaccination arms was 17 yr (8–27). The age, sex, study site, and anti-EBOV IgG levels of >200 Elisa units/mL (EU/mL) prior to vaccination were well-balanced among arms. Study sites were located in Guinea (36%), Liberia (18%), Mali (23%), and Sierra Leone (23%). In the Ad26–MVA and rVSV–booster arms, the peak anti-EBOV IgG response was observed at 3 mo (28 days after administration of the second dose). In contrast, in the rVSV single-dose arm, the peak response occurred earlier, at 28d (Table A in S2 Text).

**Table 1:** Sociodemographic and immunological characteristics at inclusion of the 1,859 participants, in the PREVAC randomized trial^A^.

Characteristic	Total, *n* = 1,859	Ad26-MVA, *n* = 749	rVSV, *n* = 739	rVSV-booster, *n* = 371
**Age**				
Median (IQR) – yr	17 (8–27)	17 (8–27)	16 (9–27)	17 (8–26)
Distribution – *n* (%)				
1–4 yr	310 (17%)	128 (17%)	115 (16%)	67 (18%)
5–11 yr	324 (17%)	121 (16%)	142 (19%)	61 (16%)
12–17 yr	321 (17%)	134 (18%)	125 (17%)	62 (17%)
Adults	904 (49%)	366 (49%)	357 (48%)	181 (49%)
**Sex** – ***n* (%)**				
Male	1,013 (54%)	413 (55%)	388 (53%)	212 (57%)
Female	846 (46%)	336 (45%)	351 (47%)	159 (43%)
**Country and study site** – ***n* (%)**				
Guinea				
*Landreah*	380 (20%)	149 (20%)	155 (21%)	76 (20%)
*Mafèrinyah*	292 (16%)	120 (16%)	116 (16%)	56 (15%)
Liberia				
*RH*	330 (18%)	130 (17%)	133 (18%)	67 (18%)
Mali				
*UCRC*	203 (11%)	82 (11%)	83 (11%)	38 (10%)
*CVD*	221 (12%)	89 (12%)	87 (12%)	45 (12%)
Sierra Leone				
*Mambolo*	433 (23%)	179 (24%)	165 (22%)	89 (24%)
**Baseline IgG anti-EBOV GP**_**1,2**_ concentration*				
Median (IQR) - EU/mL	79 (37–137)	80 (34–140)	78 (38–136)	83 (37–136)
< 66.96 EU/mL - *n* (%)	792 (43%)	323 (44%)	316 (44%)	153 (41%)
≥ 66,96 < 200 EU/mL - *n* (%)	814 (44%)	329 (44%)	317 (44%)	168 (46%)
≥ 200 EU/mL - *n* (%)	231 (13%)	90 (12%)	93 (13%)	48 (13%)

^A^CVD, Center for Vaccine Development; IQR, interquartile range; RH, Redemption Hospital; UCRC, University Clinical Research Center.

*An immunoglobulin G concentration against Ebola virus of at least 200 EU/mL was considered to indicate positivity. The assay developer’s lower limit of quantification was 66.96 EU/mL. The median concentration and geometric mean concentrations were assessed among participants with a result.

### Time of vaccination

**Fig 1:**
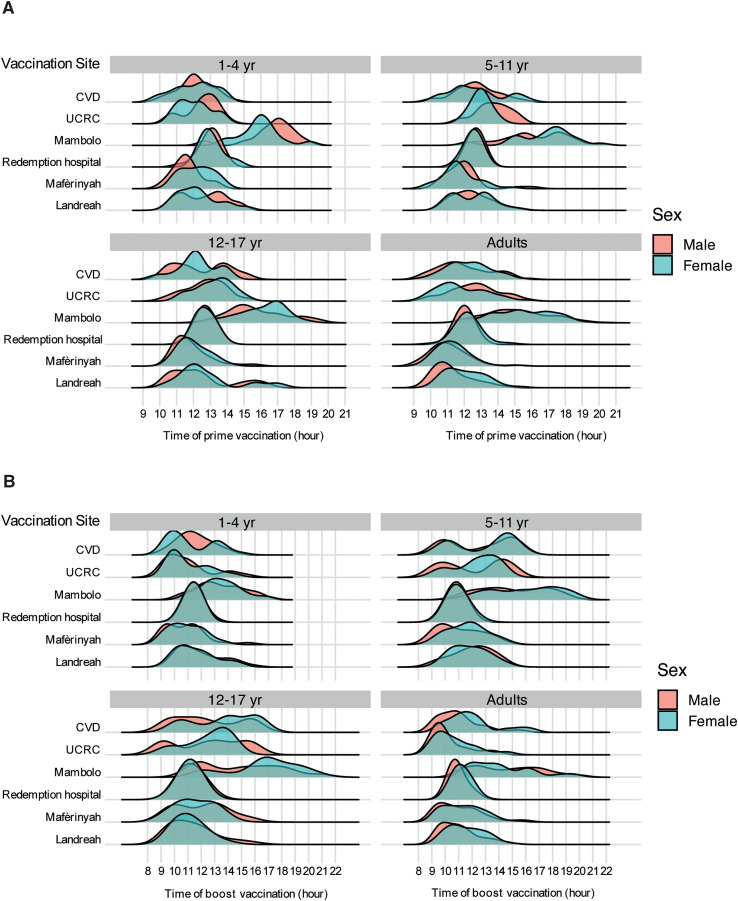
Distribution of vaccination times of first vaccination on Day 0 (A) and second vaccination at 56 d (B) by sex and age group across study sites in the PREVAC trial. CVD, Center for Vaccine Development (Mali); RH, Redemption Hospital; UCRC, University Clinical Research Center.

The times for first dose were 0918–2038 h and second dose were 0856–2113 h (Fig 1 and Table B in S2 Text). Most vaccinations were during midday hours (1100–1400 h), accounting for 70% of first dose (Day 0) and 50% of second dose (at 56 d). Vaccines administered before 1100 h represented 14% of first dose and 37% of second dose, whereas those administered after 1500 h accounted for 16% of first dose and 13% of second dose (Table B in S2 Text).

Men and women were vaccinated within similar time ranges on both Day 0 and at 56 d (Figs 1 and C in S2 Text). Vaccination times were broadly consistent across sites, with most participants receiving their doses during midday hours, except at the Mambolo site in Sierra Leone, where vaccinations occurred predominantly in the afternoon, with a median time [Interquartile range] of 1506 h [1404–1701] for first dose and 1406 h [1205–1609] for second dose (Fig 1 and Table C in S2 Text).

Although a general pattern in the timing of vaccination was observed across age groups, some differences emerged. On Day 0, adults tended to be vaccinated earlier in the day (1203 h [1103–1308]) compared to children 17 yr of age or younger across the age subgroups: 1–4 yr (1205 h [1106–1304]), 5–11 yr (1208 h [1109–1404]), and 12–17 yr (1209 h [1200–1408]) (Figs 1 and B and Table D in S2 Text). Similarly, at 56 d, children 1–4 yr of age were generally vaccinated earlier (1103 h [1004–1204]) than adults (1104 h [1004–1209]), while children 5–11 yr (1200 h [1006–1400]) and 12–17 yr (1201 h [1009–1404]) were vaccinated later than adults (Table D in S2 Text). The time of the first and second vaccination were moderately correlated (*r* = 0.5; *p*-value <0.001), regardless of the study site (Fig C in S2 Text).

### Association between time of vaccination and the durability of anti-EBOV GP_1,2_ IgG antibody response (12 mo)

In the primary analysis assessing the effect of vaccination timing on the durability of the antibody response at 12 mo post-vaccination in the overall population (all vaccinated participants, regardless of vaccination strategy), there was a statistically significant association between the time of the first vaccination and anti-EBOV GP_1,2_ IgG antibody levels at 12 mo, adjusted for age, sex, study site, and pre-vaccination antibody concentration (multiplicity-adjusted *p*-values = 0.02) (Tables E and F in S2 Text).

**Fig 2:**
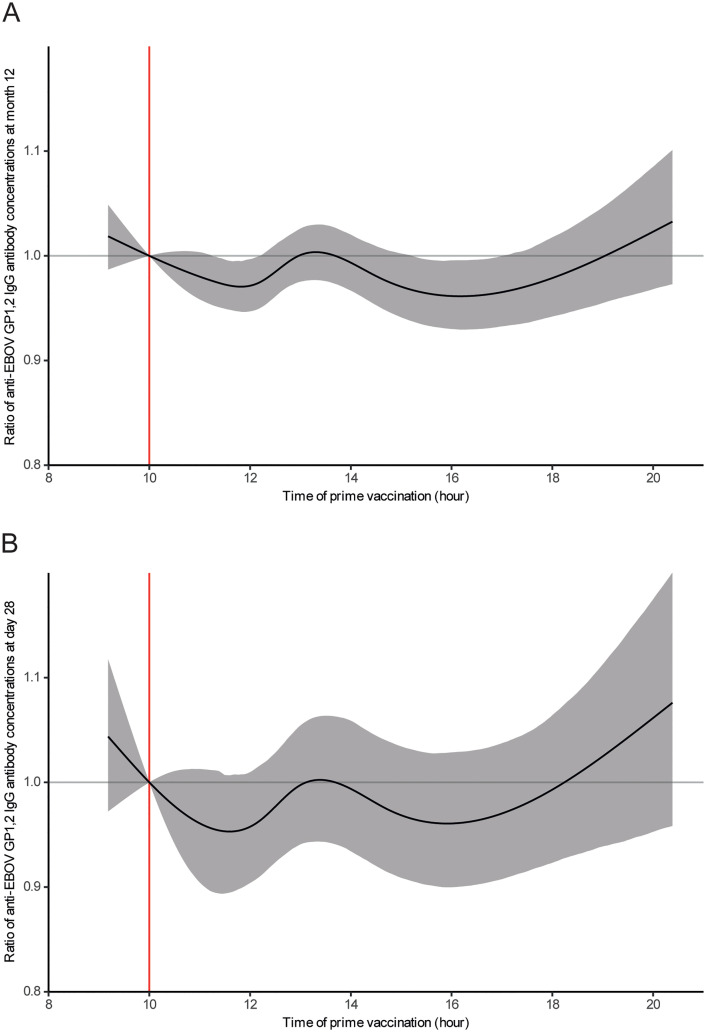
Effects of prime vaccination time on antibody response (A) measured at 12 mo in the overall population and (B) measured at 28 d in Ad26-MVA arm post-vaccination, in the PREVAC trial. The solid black line represents the median effect of vaccination time on the antibody response. The shaded area corresponds to the 95% confidence interval. The red line at 1000 h indicates the reference time against which comparisons were made with other times of the day. EBOV, Ebola virus; GP_1,2_, glycoprotein; IgG, immunoglobulin G.

Fig 2A illustrates this effect (reference time: 1000 h), showing an estimated increase in the antibody concentration ratio for participants vaccinated before 1000 h compared to those vaccinated at 1000 h and showing a gradual decline in the ratio, which reached a minimum around 1200 h. This ratio increased again, peaking near 1400 h, and declined after 1400 h until 1600 h. The maximum reduction in antibody levels at 12 mo was relatively small, estimated for participants vaccinated at 1600 h, with a decrease of 1–7% compared to those vaccinated at 1000 h (median [IQR]: 778 [556–1307] vs. 988 [716–1657] EU/mL; ratio [95% confidence interval [CI]: 0.96 [0.93–0.99]).

In the three analyses stratified by vaccination strategy—rVSV, rVSV-booster, and Ad26-MVA arms—there were no statistically significant associations between the time in the day of the first vaccination and participants’ anti-EBOV GP_1,2_ IgG antibody levels at 12 mo (multiplicity-adjusted *p*-values = 0.08, 0.11, and 0.14, respectively). Similarly, no statistically significant associations were observed between the time in the day of the second vaccination (at 56 d) and participants’ anti-EBOV GP_1,2_ IgG antibody levels at 12 mo, both in the overall population and in the analyses stratified by vaccination strategy (Tables E, G to L in S2 Text). Nevertheless, the same trend in the variation of the effect of vaccination time on the antibody response was observed for both first and second vaccination, except in the rVSV arm (Figs D to I in S2 Text).

### Association between vaccination time and the early anti-EBOV GP_1,2_ IgG antibody response (28 d, 3 mo)

At 28 d, there was no statistically significant association between the anti-EBOV GP_1,2_ IgG antibody response and the time in the day of the first vaccination in the overall population across all vaccination strategies (multiplicity-adjusted = 0.06) or in the pooled rVSV arms (multiplicity-adjusted = 0.69) (Table E and Figs J and K in S2 Text). However, in the Ad26-MVA arm, there was a statistically significant association (multiplicity-adjusted = 0.03) between the time in the day of the first vaccination (spline) and participants’ anti-EBOV GP_1,2_ IgG antibody levels at 28 d (Table E in S2 Text), adjusted for age, sex, study site, and pre-vaccination antibody concentration. Fig 2B illustrates that, similarly to 12 mo, at 28 d in the Ad26-MVA arm, antibody levels were estimated to be lower for participants vaccinated at 1600 h compared to those vaccinated at 1000 h (median [IQR], 378 [252–757] vs. 493 [290–1102] EU/mL; ratio [95% CI], 0.96 [0.89–1.02]) (Fig 2B).

At 3 mo, no statistically significant association was found between the time of the first or second vaccination and anti-EBOV GP_1,2_ IgG antibody levels (Table E and Figs L to P in S2 Text).

## Discussion

Using data from the large PREVAC randomized trial, which assessed EVD vaccination strategies in Western Africa, this study identified an association between the time in the day of first vaccination and the anti-EBOV GP_1,2_ IgG antibody levels at 12 mo, based on pooled data from the three vaccine arms. In contrast, no significant association was found for the time of second vaccination. Secondary analyses at 28 d and 3 mo revealed the absence of significant associations, except in the Ad26-MVA arm at 28 d. Overall, the findings suggest a potential influence from the circadian rhythm, with slightly stronger antibody responses estimated when vaccination occurred in the morning compared to the afternoon. However, the role of the circadian rhythm seems modest and likely to have minimal effect on antibody levels.

To our knowledge, this study is the first to investigate this association between the time of vaccination and the immune response in the context of EVD vaccines. To date, only a limited number of epidemiological studies have explored this relationship across different vaccines and pathogens. Several studies on vaccines against influenza, tuberculosis, and coronavirus disease 2019 reported stronger serological responses when administered in the morning compared to those given in the afternoon or evening, aligning with the trends observed in our results [20–25]. The underlying mechanisms for this phenomenon likely stem from the immune system’s dependence on time of day, exhibiting 24-h rhythmicity both at rest and during activation*.* While the circadian rhythmicity of the adaptive immune system has been less extensively studied than that of the innate immune system, several key mechanisms have been identified. Among other factors, these include the trafficking of B and T lymphocytes, the regulation of T-cell activation and proliferation, and the influence of clock genes on gene expression [39]. Notably, the circadian clock of CD8^+^ T cells plays a role in regulating the vaccine response. It shapes the gene expression program of these cells, making them more receptive to enhanced activation and proliferation, depending on the time of day [40,41]. Additionally, another underlying hypothesis suggests that low exposure to ambient light, via the retinohypothalamic projections to the suprachiasmatic nucleus in the brain, leads to a reduced amplitude of rest–activity cycles and possibly other circadian rhythms, which may, in turn, result in lower production of specific antibodies [42].

However, the findings from epidemiological studies are inconsistent, with several studies reporting negative results or even opposite trends, such as increased anti-spike-protein antibody responses to vaccination against coronavirus disease 2019 in individuals inoculated later in the day [26,27]. It is important to note that the evidence largely stems from observational studies with exploratory analyses, which are subject to several methodological limitations. These include a lack of statistical power due to reliance on convenience samples and the failure to account for multiple testing corrections. Statistical analyses often treat the time of vaccination as a qualitative variable, with significant heterogeneity in time cutoffs that are frequently arbitrary or unjustified. Moreover, many studies may suffer from residual confounding biases involving factors associated with both the time of vaccination and the immune response [25,28,30,43]. To date, only one large-scale clinical trial with a cluster-randomized design has been conducted, specifically in the context of influenza vaccination [44]. These limitations emphasize the need for robust methodological approaches to study this complex association.

Our study’s primary strength lies in the robust design of the PREVAC trial, which included a large Western African population, used the prospective recording of vaccination time, and measured antibody levels with the standardized Filovirus Animal Non-Clinical Group (FANG) assay. Analyses used a rigorous methodological approach, using multivariable linear models with natural splines to capture non-linear relationships and maximize statistical power. Adjustments for potential confounders and multiple testing further enhanced the robustness and reliability of our findings. The large sample size of the PREVAC trial provided high statistical power, enabling the detection of a statistically significant effect even if it was not biologically meaningful. The absence of a significant effect in the analyses conducted within each vaccine arm is likely due to the reduced statistical power in these subgroup analyses, resulting from smaller sample sizes.

Our study has some limitations. The effects of vaccination time were assessed in an observational approach, and residual confounding cannot be ruled out, particularly due to the lack of data on participants’ behaviors and lifestyle habits, such as rest–activity cycles and/or sleep patterns, which could influence the time of vaccination [45]. Future studies incorporating sleep-related data would help clarify the role of circadian rhythms in vaccine-induced immune responses.

Although vaccination times were accurately recorded in an electronic case report form, variations in site-specific practices led to heterogeneous time distributions. For example, while most sites vaccinated in the morning, the Mambolo site in Sierra Leone predominantly vaccinated in the afternoon; this was due to specific logistical constraints, because the vaccination team was based in Kambia, a 45-min drive away. Thus, there was sparse afternoon data and a non-uniform distribution, potentially limiting the ability to detect effects tied to circadian rhythms and reducing the generalizability of the findings to other vaccination times. Additionally, the apparent trend suggesting a potential increase in immunogenicity after 1800 h is not interpretable and is likely driven by the lack of data beyond this time point, as indicated by the wide confidence intervals, making these observations unreliable. The narrower time range observed for boost schedules may also explain the absence of an effect for this second injection.

A major challenge in vaccinology is understanding how to induce durable protective immunity. While some vaccines, such as the live attenuated yellow fever vaccine, are known to elicit long-lasting antibody responses that can protect recipients for a lifetime, waning antibody responses have been documented with other vaccine types, including those approved for use against EVD [4,34]. This waning protection highlights the importance of identifying factors that can influence the durability of the immune response. In our study, small and isolated statistically significant associations between vaccination time and antibody responses were observed in some analyses, but these findings were not consistent across vaccine regimens or timepoints, and the overall effect of vaccination time remained limited. The magnitude of this effect in overall population (with a range of 1–7%) is relatively modest compared to other key factors previously identified in the PREVAC study for both rVSVΔG-ZEBOV and Ad26.ZEBOV, MVA-BN-Filo [34]. Factors, including age, sex, geographic location, and pre-vaccination antibody levels, were shown to have a greater influence on vaccine-induced immune responses than the circadian rhythm effect observed in our study [34]. Notably, a previous modeling study based on data from EBOVAC trials found that geographic location had a pronounced impact, with stronger immune responses to Ad26.ZEBOV, MVA-BN-Filo observed in participants in Europe compared to those in Eastern Africa [46].

If circadian rhythm influences human vaccination responses, the time of vaccination may represent a simple, appealing, and cost-effective way to enhance vaccine efficacy. However, our findings suggest that this approach has a limited influence on immune responses to EVD vaccines. However, the effect may vary depending on the type of vaccine and pathogen. Therefore, it is crucial to evaluate this strategy using robust methodologies. If a significant effect were identified, it could be readily integrated into vaccination programs to improve outcomes.

This study highlights a modest association between vaccination timing and antibody response, suggesting a potential role for circadian rhythm in shaping immune responses after administration of EVD vaccines. However, the estimated effect is limited and likely too small to warrant recommendations regarding chronovaccination for EVD vaccines. Nevertheless, these results encourage further investigation of the role of chronovaccination for vaccines in other contexts, through high-quality studies with robust methodology. Such efforts could pave the way for the development of simple strategies that enhance global public health outcomes.

## Supporting information

S1 Text Fig A. Directed Acyclic Graph (DAG) developed using Dagitty software.
(DOCX)

S2 Text Table A. Distribution of antibody concentration (anti-EBOV GP_1,2_ IgG) at 28 d, 3 mo, and 12 mo post-vaccination. **Table B.** Description of vaccination times on Day 0 and Day 56 among the 1,859 participants, PREVAC trial. **Table C.** Description of vaccination times on Day 0 and Day 56 among the 1,859 participants per study sites, PREVAC trial. **Table D.** Description of vaccination times on Day 0 and Day 56 among the 1,859 participants per age, PREVAC trial. **Table E.** Multivariable linear regression analyzes for evaluating the association between time of day of vaccination (first and second doses, respectively) and anti-EBOV GP_1,2_ IgG antibody response measured at 28 d, 3 mo, and 12 mo based on the data from the PREVAC trial. **Table F.** Parameter estimates evaluating the association between the time of prime vaccination and the IgG anti-EBOV GP_1,2_ antibody immune response at 12 mo in all vaccinated participants. **Table G.** Parameter estimates evaluating the association between time of prime vaccination and the immune response of IgG anti-EBOV GP_1,2_ at 12 mo in participants vaccinated with rVSV (one dose). **Table H.** Parameter estimates evaluating the association between time of prime vaccination and the immune response of IgG anti-EBOV GP_1,2_ at 12 mo in participants vaccinated with rVSV (two doses, with dose 2 at Day 56). **Table I.** Parameter estimates evaluating the association between time of prime vaccination and the immune response of IgG anti-EBOV GP_1,2_ at 12 mo in participants vaccinated with Ad26-MVA. **Table J.** Parameter estimates evaluating the association between time of second vaccination and the immune response of IgG anti-EBOV GP_1,2_ at 12 mo in the participants receiving prime-boost strategy (rVSV- booster or Ad26-MVA arm) in the PREVAC trial. **Table K.** Parameter estimates evaluating the association between time of second vaccination and the immune response of IgG anti-EBOV GP_1,2_ at 12 mo in participants in the rVSV-booster arm. **Table L.** Parameter estimates evaluating t the association between time of second vaccination and the immune response of IgG anti-EBOV GP_1,2_ at 12 mo in participants in the Ad26-MVA arm of the PREVAC trial. **Fig A.** Flow chart of *post hoc* analysis, PREVAC trial. **Fig B.** Distribution of vaccination times of first vaccination Day 0 (A and B) and second vaccination 56 d (C and D) by sex and age group across study sites in the PREVAC trial. **Fig C.** Correlation between first vaccination times (Day 0) and second vaccination times (Day 56) by study site, PREVAC trial. **Fig D.** Effect of prime vaccination time on antibody response measured at 12 mo post-vaccination in the PREVAC trial in rVSV arm. **Fig E.** Effect of prime vaccination time on antibody response measured at 12 mo post-vaccination in the rVSV-booster arm of the PREVAC trial. **Fig F.** Effect of prime vaccination time on antibody response measured at 12 mo post-vaccination in the PREVAC trial in AD26-MVA arm. **Fig G.** Effect of second administration time on antibody response measured at 12 mo post-vaccination in the PREVAC trial in the overall population. **Fig H.** Effect of second administration time on antibody response measured at 12 mo post-vaccination in the PREVAC trial in rVSV-booster arm. **Fig I.** Effect of second administration time on antibody response measured at 12 mo post-vaccination in the PREVAC trial in AD26-MVA arm. **Fig J.** Effect of first vaccination time on antibody response measured at Day 28 post-vaccination in the PREVAC trial in the overall population. **Fig K.** Effect of first vaccination time on antibody response measured at Day 28 post-vaccination in the PREVAC trial in the pooled rVSV arm. **Fig L.** Effect of first vaccination time on antibody response measured at 3 mo post-vaccination in the PREVAC trial in the overall population. **Fig M.** Effect of first vaccination time on 3-month antibody response in the rVSV-booster arm. **Fig N.** Effect of first vaccination time on antibody response measured at 3 mo post-vaccination in the PREVAC trial in AD26-MVA arm. **Fig O.** Effect of second administration time on antibody response measured at 3 mo post-vaccination in the PREVAC trial in the overall population. **Fig P.** Effect of second administration time on antibody response measured at 3 mo post-vaccination in the PREVAC trial in rVSV-booster arm. **Fig Q.** Effect of second administration time on antibody response measured at 3 mo post-vaccination in the PREVAC trial in AD26-MVA arm.
(DOCX)

S3 Text FANG assay. (DOCX)

S4 Text PREVAC study team. (DOCX)
