# Supplementary material for: Effect of the time of day for vaccination on the immune response to Ebola Virus Disease vaccines: A modeling study from PREVAC randomized trial
Source: PLoS Negl Trop Dis. 2026 Jan 30;20(1):e0013950. doi: 10.1371/journal.pntd.0013950 (PMC12893655; doi:10.1371/journal.pntd.0013950)
Supplement: S1 Text — Directed Acyclic Graph (DAG) developed using Dagitty software. (DOCX) [file pntd.0013950.s001.docx]

# Supplementary 1: methods

*Models built to evaluate the effect of vaccination time of on the level of antibody concentrations*

To investigate the association between anti-EBOV GP_1,2_ IgG levels collected at a given time point $T$ and the hour of vaccination, we built two distinct multivariate spline-based linear regression models, one including the time of the prime vaccination (Model 1) and the other with time of the boost vaccination (Model 2) as explanatory variables.

The covariates included in the models as additional explanatory variables were selected based on a directed acyclic graph (Supplementary Figure 1), developed using Dagitty software (<https://www.dagitty.net/dags.html>) and constructed based on existing literature^1^. In particular, Valayer et al. demonstrated a significant association between the anti-EBOV GP_1,2_ IgG antibody response and age, sex, country, and pre-vaccination anti-EBOV GP_1,2_ IgG levels ^2^.

- **Age** (in years) was included as a continuous explanatory variable in the models. Age is considered an important factor, because immunity and vaccine response may vary over a lifetime, with a stronger response observed in children.
- **Sex** was included as a binary variable (male and female), with male designated as the group of reference. Vaccine responses differ between sexes^1^.
- **Study site** was included as a categorical variable to account for the six different locations: CVD, Landreah (used as reference), Mafèrinyah, Mambolo, Redemption Hospital, and UCRC. This factor accounted for local environmental conditions, vaccination practices, and other contextual variables that may influence the immune response. Additionally, it enabled for adjustment based on the laboratory that analyzed the anti-EBOV GP_1,2_ IgG levels.
- **Baseline anti-EBOV GP_1,2_ IgG antibody concentration**, measured before the prime vaccination, was also included. This measure helped control for initial variations in immune response among individuals for assessment of the specific impact of vaccination on antibody response. This variable was considered as a continuous variable in the model.

The three continuous covariates—vaccination time (using a 24-h clock), age at inclusion (in years), and baseline antibody concentration at Day 0 (in EU/mL)—were integrated in the models using the natural splines functions. The use of these smooth functions enabled flexibility in the model and consideration of nonlinear dependencies between antibody concentration and explanatory variables. Different combinations of number/positions of knots for each spline function were tested and compared using Akaike information criterion for selection (i.e., the lower the better). The optimal combination corresponds to a choice of natural spline functions with four internal knots (i.e., linear combination of *K*=5 spline bases), located at quintiles (20th, 40th, 60th, 80th, and maximum) corresponding to 1130 h, 1216 h, 1294 h, and 1455 h for vaccination times; 6, 13, 20, and 31 for ages; and 1.47, 1.77, 1.99, and 2.18 for antibody levels at Day 0.

The resulting distinct models describing the log_10_ anti-EBOV GP_1,2_ antibody concentrations collected at time $T$ in participants as a function of the first (Model 1) or second (Model 2) vaccination time are defined by the following equations:

| Model 1 | $Y_{i}^{T}=\beta_{0}+\sum_{k=1}^{K=5} \beta_{hk} f_{hk}\left( \boldsymbol{H}_{\boldsymbol{i}}^{\boldsymbol{1}} \right)+\sum_{k=1}^{K=5} \beta_{ak} f_{ak}\left( Age_{i} \right)+\sum_{k=1}^{K=5} \beta_{ank} f_{ank}({Ab}_{i}^{D0})+\beta_{\mathrm{Sex}}\mathbb{I}\left( \mathrm{Sex}_{i}=female \right)+ \sum_{j=1}^{J=5} \beta_{site_{j}}\mathbb{I(}Site_{i}=Site j)+\varepsilon_{i}$ |
| --- | --- |
| Model 2 | $Y_{i}^{T}=\beta_{0}+\sum_{k=1}^{K=5} \beta_{hk} f_{hk}\left( \boldsymbol{H}_{\boldsymbol{i}}^{\boldsymbol{2}} \right)+\sum_{k=1}^{K=5} \beta_{ak} f_{ak}\left( Age_{i} \right)+\sum_{k=1}^{K=5} \beta_{ank} f_{ank}({Ab}_{i}^{D0})+\beta_{\mathrm{Sex}}\mathbb{I}\left( \mathrm{Sex}_{i}=female \right)+ \sum_{j=1}^{J=5} \beta_{site_{j}}\mathbb{I(}Site_{i}=Site j)+\varepsilon_{i}$ |

where $Y_{i}^{T}$ represents the log_10_-transformed IgG antibody response against EBOV GP_1,2_ observed for participant *i* collected at $T$ corresponding either to 28 d (only for Model 1), 3 mo, or 12 mo, depending on the objective. The parameters $\beta_{hk}$, $\beta_{ak}$, and $\beta_{ank}$ are the regression coefficients associated with the spline functions $f_{hk}$, $f_{ak}$, and $f_{ank}$ for the time of vaccinations $H^{1}$ or $H^{2}$, age, and antibodies, respectively, and where $f_{hk}\left( H_{i}^{1} \right)$, $f_{hk}(H_{i}^{2})$, $f_{ak}\left( Age_{i} \right)$, and $f_{ank}(Ab_{i}^{D_{0}})$ represent the *k^th^* spline basis functions evaluated for the time of prime vaccination, time of second vaccination, age, and baseline antibody concentration of participant, respectively. In Model 2, the vaccination time $H^{2}$ corresponds to the second administered at 56 d in the two-dose strategies. The parameter $\beta_{Sex}$ is the regression coefficient associated with the binary variable Sex, with male as the reference, while the parameters ${\beta_{site}}_{j}$ correspond to the regression coefficients associated with the effect of the different sites on the antibody response at time$T$ relative to the reference site (*j*=1 for Maférinyah, *j*=2 for Redemption Hospital, *j*=3 for Mambolo, *j*=4 for UCRC, and *j*=5 for CVD), with Landreah as the reference. Finally, the variable $\varepsilon_{i}$ is the constant random error term for observation *i*, assumed to follow a normal distribution of mean 0 and standard deviation $\sigma$, $\varepsilon_{i}\approx n\left( 0,\sigma^{2} \right)$. The models were evaluated independently for each vaccination arm.

*Visualization of the effect of vaccination time on immune response*

Because the effect of vaccination time on the IgG antibody response against EBOV GP_1,2_ collected at time $T$ was modeled using a spline function, a simple analysis of the estimated parameters was not sufficient for interpretation. To evaluate the effect, the ratio of predicted antibody concentrations at time $T$ post-vaccination between a given vaccination time $H$ and a fixed reference vaccination time $H_{ref}$ (set at 1000 here) was calculated for each participant profile and for each model.

In particular, this ratio was considered for average participant profiles within the overall population, meaning average age at inclusion ($\bar{age}$), baseline antibody levels at Day 0 ($\bar{Ab D0}$), and for fixed sex, and study site. Accordingly, the ratio of antibody concentrations predicted at time $T$ by the model $m$ (i.e., evaluating the effect of the time of the $m^{th}$ vaccination) for a profile of Sex = sex and Site = site is labelled $\mathcal{R}_{sex,site}^{m,T}$ and is given by:

$$\mathcal{R}_{sex, site}^{m, T}(H)=\frac{\hat{Y}^{T}(\boldsymbol{H}^{\boldsymbol{m}}\boldsymbol{=H}, Age= \bar{age}, Ab^{D_{0}}=\bar{Ab D0}, Sex=sex, Site=site)}{\hat{Y}^{T}(\boldsymbol{H}^{\boldsymbol{m}}\boldsymbol{=}\boldsymbol{H}_{\boldsymbol{ref}}, Age= \bar{age}, Ab^{D_{0}}=\bar{Ab D0}, Sex=sex, Site=site)}$$

where $\hat{Y}^{T}$ corresponds to the log_10_-transformed antibody concentration at time $T$ predicted by model $m$. Thus, for the profile corresponding to men (i.e., reference sex) at Landreah (i.e., reference site), the ratio at 12 mo for a given vaccination time *H* of the $m^{th}$ vaccination was evaluated as:

$$\mathcal{R}_{male,landreah}^{m, M12}(H)=\frac{\hat{\beta}_{0}+ \sum_{k=1}^{5} \hat{\beta}_{hk}f_{hk}\left( H^{m}=\boldsymbol{H} \right)+ \sum_{k=1}^{5} \hat{\beta}_{ak}f_{ak}\left( \bar{age} \right)+ \sum_{k=1}^{5} \hat{\beta}_{ank}f_{ank}(\bar{Ab D0})}{\hat{\beta}_{0}+ \sum_{k=1}^{5} \hat{\beta}_{hk}f_{hk}\left( H^{m}=\boldsymbol{H}_{\boldsymbol{ref}} \right)+ \sum_{k=1}^{5} \hat{\beta}_{ak}f_{ak}\left( \bar{age} \right)+ \sum_{k=1}^{5} \hat{\beta}_{ank}f_{ank}(\bar{Ab D0})}$$

The distribution of this ratio (mean value and its 95% CI) for each tested vaccination time was calculated by simulation. Its calculation enabled the identification of specific times of vaccination associated with antibody concentrations significantly different from the concentration predicted at the reference time of vaccination $H_{ref}.$ To do this, a classic bootstrap approach was applied:

1. We randomly sampled with replacement *n* individuals from the original dataset, where *n* is the number of participants in the original dataset.
2. We estimated the regression model of interest (1 or 2) on the simulated dataset and computed antibody concentration ratios for each participant profile and all vaccination times belonging to the regular grid of 200 values in the range of vaccination time observed in the original dataset.

We repeated steps a and b 1,000 times, enabling the calculation of the mean and 95% CI of the ratio.

To facilitate the visualization of these results, graphs were generated illustrating the evolution of the ratio as a function of the tested vaccination time *H*. This method was applied for all vaccination arms and all time of antibody concentration of interest $T$.


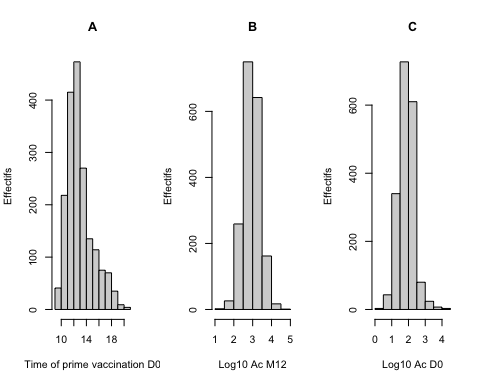

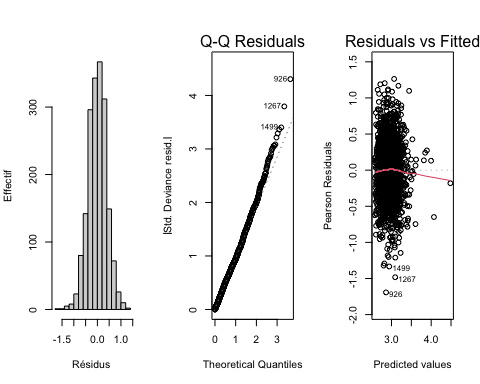


Shown above are distribution of time of prime vaccination (A), log_10_ anti-EBOV GP_1,2_ antibody concentrations on month 12 and Day 0 respectively (B, C), distribution and q-q plots of the residuals and random effects. Finally predicted values between residuals and fitted estimated on log_10_ anti-EBOV GP_1,2_ antibody concentrations in all participants group (1,859 participants). EBOV = Ebola virus. GP_1,2_ = glycoprotein. Similar results were obtained for models estimated on the distinct vaccination groups.


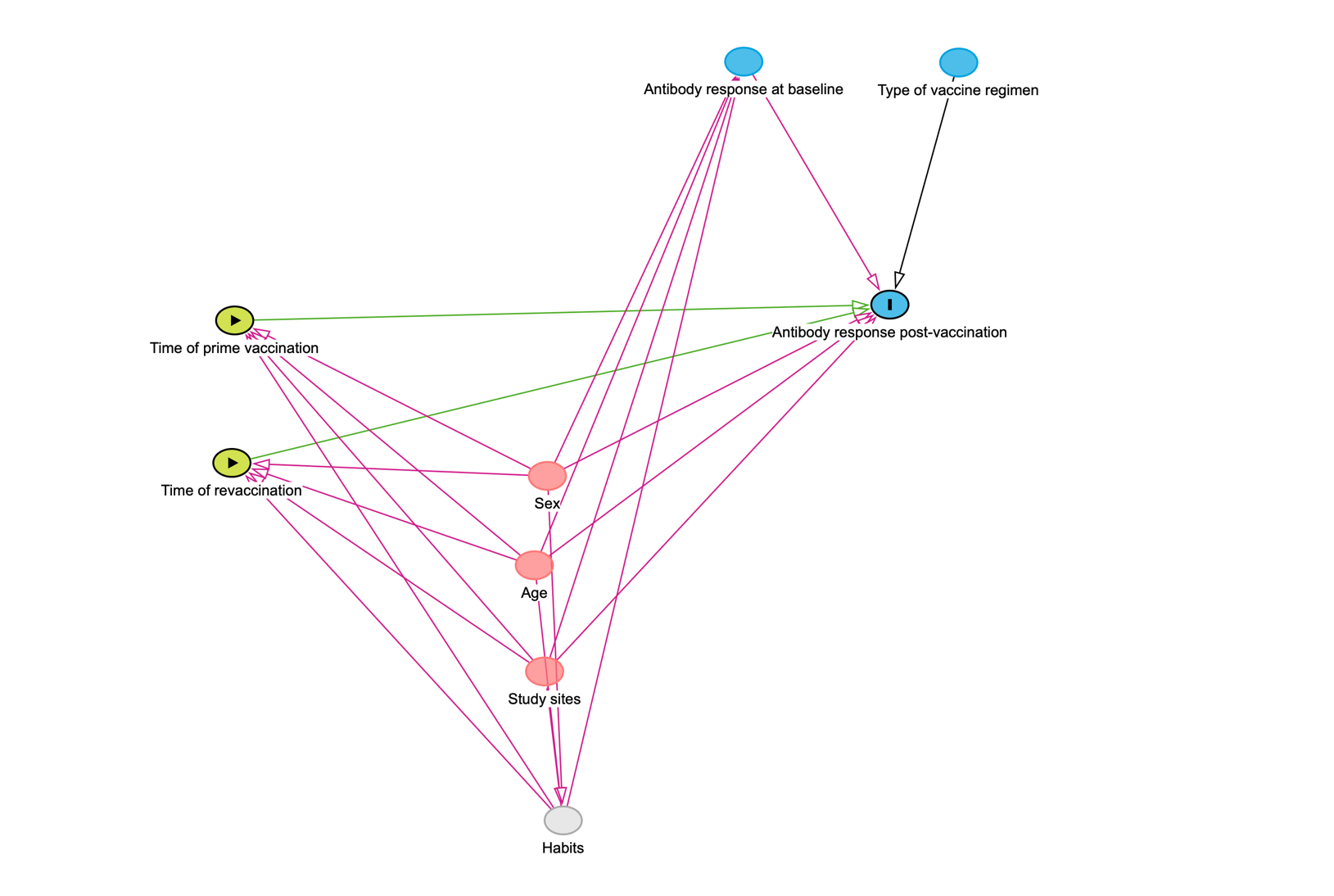


## **S1 Fig A.** Directed Acyclic Graph (DAG) developed using Dagitty software.

Antecedent factors of exposure and outcome (sex, age, and study sites) are shown in pink; events (antibody response at baseline, vaccine strategy, and antibody response post-vaccination) are shown in blue; exposure, or time of first and second vaccinations, are shown in green; unobservable variables (habits) are shown in gray.
