# Supplementary material for: Effect of the time of day for vaccination on the immune response to Ebola Virus Disease vaccines: A modeling study from PREVAC randomized trial
Source: PLoS Negl Trop Dis. 2026 Jan 30;20(1):e0013950. doi: 10.1371/journal.pntd.0013950 (PMC12893655; doi:10.1371/journal.pntd.0013950)
Supplement: S2 Text — Distribution of antibody concentration (anti-EBOV GP1,2 IgG) at 28 d, 3 mo, and 12 mo post-vaccination. Table B. Description of vaccination times on Day 0 and Day 56 among the 1,859 participants, PREVAC trial. Table C. Description of vaccination times on Day 0 and Day 56 among the 1,859 participants per study sites, PREVAC trial. Table D. Description of vaccination times on Day 0 and Day 56 among the 1,859 participants per age, PREVAC trial. Table E. Multivariable linear regression analyzes for evaluating the association between time of day of vaccination (first and second doses, respectively) and anti-EBOV GP1,2 IgG antibody response measured at 28 d, 3 mo, and 12 mo based on the data from the PREVAC trial. Table F. Parameter estimates evaluating the association between the time of prime vaccination and the IgG anti-EBOV GP1,2 antibody immune response at 12 mo in all vaccinated participants. Table G. Parameter estimates evaluating the association between time of prime vaccination and the immune response of IgG anti-EBOV GP1,2 at 12 mo in participants vaccinated with rVSV (one dose). Table H. Parameter estimates evaluating the association between time of prime vaccination and the immune response of IgG anti-EBOV GP1,2 at 12 mo in participants vaccinated with rVSV (two doses, with dose 2 at Day 56). Table I. Parameter estimates evaluating the association between time of prime vaccination and the immune response of IgG anti-EBOV GP1,2 at 12 mo in participants vaccinated with Ad26-MVA. Table J. Parameter estimates evaluating the association between time of second vaccination and the immune response of IgG anti-EBOV GP1,2 at 12 mo in the participants receiving prime-boost strategy (rVSV- booster or Ad26-MVA arm) in the PREVAC trial. Table K. Parameter estimates evaluating the association between time of second vaccination and the immune response of IgG anti-EBOV GP1,2 at 12 mo in participants in the rVSV-booster arm. Table L. Parameter estimates evaluating t the associa [file pntd.0013950.s002.docx]

**Supplementary 2 : Results**

# Description of the selection of participants from the PREVAC trial

- 19 missed visits

- 2 pregnant women

- 1 incorrect boost strategy received

- 1 other

(*n*=23)

Adults and children included in the PREVAC trial **(population intention to treat)**

*n*=2,801

Ad26-MVA

*n*=799

rVSV

*n*=802

rVSV-booster

*n*=399

Placebo

*n*=801

Ad26-MVA

*n*=776

rVSV

*n*=774

rVSV-booster

*n*=385

Placebo

*n*=773

- 36 missed visits

- 2 pregnant women

- 1 refusal

- 1 dropout

- 1 incorrect boost strategy received

- 1 other

(*n*=42)

14 missed boost visits

- 22 missed visits

- 3 pregnant women

- 1 dropout

- 1 death

- 1 incorrect boost strategy received

- 1 other

(*n*=29)

Randomisation 2:2:1:2

Final study population

*n*=1,859

71 missing data points on antibody measurement at 12 mo and 5 on vaccination time at 56 d

Ad26-MVA

*n*=749

rVSV

*n*=739

rVSV-booster

*n*=371

## **S2 Fig A.** Flow chart of *post hoc* analysis, PREVAC trial.

The Partnership for Research on Ebola VACcinations (PREVAC) trial (version 4.0) included 2,801 participants (1,400 adults and 1,401 children), with 799 randomized to the Ad26-MVA arm, 802 to the rVSV arm, 399 to the rVSV-booster arm, and 801 to the placebo arm. This diagram illustrates the selection process of the study. In accordance with the protocol, population analysis consisted of 1,859 participants, including 749 in the Ad26-MVA arm, 739 in the rVSV arm, and 371 in the rVSV-booster arm.

# Description of antibody concentration and vaccination time

## **S2 Table A.** Distribution of antibody concentration (anti-EBOV GP_1,2_ IgG) at 28 d, 3 mo, and 12 mo post-vaccination.

| **Characteristic** | **Total**  ***n* = 1,859** | **Ad26–MVA**  ***n* = 749** | **rVSV**  ***n* = 739** | **rVSV–booster**  ***n* = 371** |
| --- | --- | --- | --- | --- |
| **Antibody concentration at 28 d post-vaccination (EU/mL)** |  |  |  |  |
| *Median (IQR)* | 860.8 (396.5–1,897.7) | 416.0 (227.8–754.3) | 1,451.7 (740.8–2,828.2) | 1,404.2 (769.0–2,530.8) |
| *Minimum; maximum* | 7.9; 70,522.3 | 20.8; 70,522.3 | 7.9; 25,868.2 | 29.9; 62,739.5 |
| **Antibody concentration at 3 mo post-vaccination (EU/mL)** |  |  |  |  |
| *Median (IQR)* | 2,742.9 (1,298.5–6,355.6) | 5,754.7 (2,714.4–12,862.7) | 1,258.6 (730.8–2,081.8) | 3,882.7 (2,642.5–6,338.0) |
| *Minimum; maximum* | 18.2; 117,866.0 | 69.5; 117,866.0 | 24.8; 26,193.0 | 18.2; 48,940.8 |
| **Antibody concentration at 12 mo post-vaccination (EU/mL)** |  |  |  |  |
| *Median (IQR)* | 884.8 (459.2–1,707.8) | 532.0 (291.6–989.0) | 1,169.5 (656.1–2,050.5) | 1,235.6 (745.5–2,371.7) |
| *Minimum; maximum* | 14.8; 34,792.8 | 14.8; 34,792.8 | 40.8; 21,959.5 | 52.6; 20,074.9 |

**EBOV, Ebola virus; GP_1,2_, glycoprotein; IgG, immunoglobulin G; IQR, Interquartile range.**

## **S2 Table B.** Description of vaccination times on Day 0 and Day 56 among the 1,859 participants, PREVAC trial.

|  | **Total**  ***n* = 1,859** | **Ad26-MVA**  ***n* = 749** | **rVSV**  ***n* = 739** | **rVSV-booster**  ***n* = 371** |
| --- | --- | --- | --- | --- |
| **Dose 1 (Day 0) time of vaccination min–max** | 0918–2038 |  |  |  |
| Time of vaccination median (IQR) | 1250 (1152–1389) | 1250 (1152–1395) | 1252 (1152–1377) | 1257 (1151–1401) |
| Distribution - *n* (%) |  |  |  |  |
| <11 h | 252 (14%) | 100 (14%) | 100 (14%) | 52 (14%) |
| 11–14 h | 1,298 (70%) | 513 (68%) | 525 (71%) | 260 (70%) |
| ≥15 h | 309 (16%) | 136 (18%) | 114 (15%) | 59 (16%) |
| **Dose 2 (Day 56) time of vaccination min–max** | 0856–2113 |  |  |  |
| Time of vaccination median (IQR) | 1158 (1050–1326) | 1155 (1052–1325) | 1152 (1043–1333) | 1177 (1058–1320) |
| Distribution - *n* (%) |  |  |  |  |
| <11 h | 691 (37%) | 274 (37%) | 284 (38%) | 133 (36%) |
| 11–14 h | 925 (50%) | 375 (50%) | 357 (48%) | 193 (52%) |
| ≥15 h | 243 (13%) | 100 (13%) | 98 (14%) | 45 (12%) |

**IQR, Interquartile range.**

## **S2 Table C.** Description of vaccination times on Day 0 and Day 56 among the 1,859 participants per study sites, PREVAC trial.

| **Characteristic** | **Total**  ***n* =1,859** | **Landreah**  ***n* = 380** | **Mafèrinyah**  ***n* = 292** | **Redemption hopital**  ***n* = 330** | **Mambolo**  ***n* = 433** | **UCRC**  ***n* = 203** | **CVD**  ***n* = 221** |
| --- | --- | --- | --- | --- | --- | --- | --- |
| **Time of first vaccination median (IQR)** | 1205 (1105–1309) | 1109 (1100–1300) | 1105 (1009–1202) | 1204 (1200–1209) | 1506 (1404–1701) | 1206 (1105–1304) | 1202 (1103–1302) |
| **Time of second vaccination median (IQR)** | 1106 (1005–1303) | 1102 (1003–1204) | 1102 (0908–1204) | 1100 (1006–1106) | 1406 (1205–1609) | 1004 (0906–1204) | 114 (1003–1303) |

**IQR, Interquartile range.**

## **S2 Table D.** Description of vaccination times on Day 0 and Day 56 among the 1,859 participants per age, PREVAC trial.

| **Characteristic** | **Total**  ***n* = 1,859** | **1-4 yr**  ***n* = 310** | **5-11 yr**  ***n* = 324** | **12-17 yr**  ***n* = 321** | **Adults**  ***n* = 904** |
| --- | --- | --- | --- | --- | --- |
| **Time of first vaccination median (IQR)** | 1205 (1105–1309) | 1205 (1106–1304) | 1208 (1109–1404) | 1209 (1200–1408) | 1203 (1103–1308) |
| **Time of second vaccination median (IQR)** | 1106 (1005–1303) | 1103 (1004–1204) | 1200 (1006–1400) | 1201 (1009–1404) | 1104 (1004–1209) |

**IQR, Interquartile range.**

## **S2 Fig B.** Distribution of vaccination times of first vaccination Day 0 (A and B) and second vaccination 56 d (C and D) by sex and age group across study sites in the PREVAC trial.

Boxplots showing the median hour of vaccination in a day by category of sex or age. The lower and upper hinges on the boxes correspond to the first and third quartiles.

CVD, Center for vaccine development (Mali); UCRC, University Clinical Research Center; RH, Redemption Hospital; MFY, Mafèrinyah.

**A** Time of prime vaccination by sex per study site; **B** Time of prime vaccination by age per study site; **C** Time of second dose by sex per study site; **D** Time of second dose by age per study site.

|  |
| --- |

## **S2 Fig C.** Correlation between first vaccination times (Day 0) and second vaccination times (Day 56) by study site, PREVAC trial.

Correlation, Pearson correlation coefficient. CVD, Center for vaccine development (Mali); UCRC, University Clinical Research Center.

# Association between the time of vaccination and antibody response

## **S2 Table E.** Multivariable linear regression analyzes for evaluating the association between time of day of vaccination (first and second doses, respectively) and anti-EBOV GP_1,2_ IgG antibody response measured at 28 d, 3 mo, and 12 mo based on the data from the PREVAC trial.

| **Linear regression models^A^** | | ***n*** | **Effect of time in the day of vaccination on antibody response** | |
| --- | --- | --- | --- | --- |
|  |  |  | ***p*-value**  **(unadjusted)** | ***p*-value**  **(adjusted for test multiplicity) ^B^** |
| **Dependent variable: Anti-EBOV GP_1,2_ IgG at 12 mo** | | | | |
| **First vaccination (Day 0)** | |  |  |  |
| All vaccinated participants | | 1,837 | 0.006 | 0.02 |
| **Model stratified by vaccine strategy** | |  |  |  |
|  | rVSV arm | 726 | 0.08 | 0.08 |
|  | rVSV-booster arm | 369 | 0.04 | 0.11 |
|  | Ad26-MVA arm | 742 | 0.14 | 0.14 |
| **Second vaccination (56 d)** | |  |  |  |
| All vaccinated participants | | 1,111 | 0.03 | 0.09 |
| Model stratified by vaccine strategy | |  |  |  |
|  | rVSV-booster arm | 369 | 0.32 | 0.48 |
|  | Ad26-MVA arm | 742 | 0.49 | 0.49 |
| **Dependent variable: Anti-EBOV GP_1,2_ IgG at 28 d** | | | | |
| All vaccinated participants | | 1,798 | 0.06 | 0.09 |
| Models stratified by vaccine strategy | |  |  |  |
|  | rVSV arms | 1,072 | 0.69 | 0.69 |
|  | Ad26-MVA arm | 726 | 0.01 | 0.03 |
| **Dependent variable: Anti-EBOV GP_1,2_ IgG (log_10_-transformed) at 3 mo (28 d post-boost)** | | | | |
| **First vaccination (Day 0)** | |  |  |  |
| All vaccinated participants | | 1,074 | 0.42 | 0.63 |
| Models stratified by vaccine strategy | |  |  |  |
|  | rVSV-booster arm | 363 | 0.69 | 0.69 |
|  | Ad26-MVA arm | 711 | 0.42 | 0.63 |
| **Second vaccination (56 d)** | |  |  |  |
| All vaccinated participants | | 1,074 | 0.29 | 0.44 |
| Models stratified by vaccine strategy | |  |  |  |
|  | rVSV-booster arm | 363 | 0.70 | 0.70 |
|  | Ad26-MVA arm | 711 | 0.26 | 0.44 |

^A^multivariable linear regression models adjusted for age, sex, study site, and baseline antibody concentration. Likelihood ratio test *p*-value assessing the association between vaccination time splines (betas) and antibody levels in the multivariable linear regression model.

^B^*p*-value adjusted for alpha risk using the Benjamini-Hochberg correction method across all tests conducted at each time point for each vaccination.

## **S2 Table F.** Parameter estimates evaluating the association between the time of prime vaccination and the IgG anti-EBOV GP_1,2_ antibody immune response at 12 mo in all vaccinated participants.

| **Characteristic** | **Beta** | **95% CI** | ***p*-value** |
| --- | --- | --- | --- |
| **Population: all vaccinated participants** |  |  |  |
| Natural splines (time of prime vaccination) |  |  | 0.006 |
| $\beta_{hk}(1)$ | -0.16 | -0.32; 0.00 |  |
| $\beta_{hk}(2)$ | 0.01 | -0.18; 0.20 |  |
| $\beta_{hk}(3)$ | -0.25 | -0.41; 0.08 |  |
| $\beta_{hk}(4)$ | -0.21 | -0.61; 0.19 |  |
| $\beta_{hk}(5)$ | 0.10 | -0.11; 0.31 |  |
| Natural splines (age) |  |  | <0.001 |
| $\beta_{ak}(1)$ | -0.06 | -0.15; 0.03 |  |
| $\beta_{ak}(2)$ | -0.47 | -0.58; 0.37 |  |
| $\beta_{ak}(3)$ | -0.31 | -0.43; 0.19 |  |
| $\beta_{ak}(4)$ | -0.59 | -0.79; 0.39 |  |
| $\beta_{ak}(5)$ | -0.32 | -0.50; 0.13 |  |
| Natural splines (pre-vaccination antibody level) |  |  | <0.001 |
| $\beta_{ank}(1)$ | 0.23 | -0.02; 0.48 |  |
| $\beta_{ank}(2)$ | 0.25 | -0.03; 0.53 |  |
| $\beta_{ank}(3)$ | 0.18 | -0.01; 0.37 |  |
| $\beta_{ank}(4)$ | 1.1 | 0.49; 1.7 |  |
| $\beta_{ank}(5)$ | 1.5 | 1.1; 1.8 |  |
| Study site |  |  | <0.001 |
| Landreah | — | — |  |
| Mafèrinyah | -0.01 | -0.07; 0.05 |  |
| Redemption Hospital | 0.03 | -0.04; 0.10 |  |
| Mambolo | -0.05 | -0.13; 0.03 |  |
| UCRC | 0.20 | 0.11; 0.28 |  |
| CVD | 0.13 | 0.05; 0.22 |  |
| Sex |  |  | <0.001 |
| Male | — | — |  |
| Female | 0.10 | 0.06; 0.14 |  |
| CI, confidence interval; CVD, Center for vaccine development (Mali); UCRC, University Clinical Research Center. | | | |

## **S2 Table G.** Parameter estimates evaluating the association between time of prime vaccination and the immune response of IgG anti-EBOV GP_1,2_ at 12 mo in participants vaccinated with rVSV (one dose).

| **Characteristic** | **Beta** | **95% CI** | ***p*-value** |
| --- | --- | --- | --- |
| **Population: rVSV one dose** |  |  |  |
| Natural splines (time of prime vaccination) |  |  | 0.081 |
| $\beta_{hk}(1)$ | -0.10 | -0.36; 0.16 |  |
| $\beta_{hk}(2)$ | 0.14 | -0.17; 0.45 |  |
| $\beta_{hk}(3)$ | -0.19 | -0.42; 0.04 |  |
| $\beta_{hk}(4)$ | 0.13 | -0.50; 0.76 |  |
| $\beta_{hk}(5)$ | -0.01 | -0.27; 0.25 |  |
| Natural splines (age) |  |  | <0.001 |
| $\beta_{ak}(1)$ | 0.09 | -0.04; 0.23 |  |
| $\beta_{ak}(2)$ | -0.32 | -0.48; -0.16 |  |
| $\beta_{ak}(3)$ | -0.09 | -0.27; 0.09 |  |
| $\beta_{ak}(4)$ | -0.27 | -0.56; 0.03 |  |
| $\beta_{ak}(5)$ | -0.24 | -0.48; -0.01 |  |
| Natural splines (pre-vaccination antibody level) |  |  | <0.001 |
| $\beta_{ank}(1)$ | 0.25 | -0.13; 0.62 |  |
| $\beta_{ank}(2)$ | 0.33 | -0.08; 0.74 |  |
| $\beta_{ank}(3)$ | 0.02 | -0.26; 0.30 |  |
| $\beta_{ank}(4)$ | 1.1 | 0.20; 1.9 |  |
| $\beta_{ank}(5)$ | 1.1 | 0.74; 1.5 |  |
| Study site |  |  | <0.001 |
| Landreah | — | — |  |
| Mafèrinyah | -0.04 | -0.13; 0.05 |  |
| Redemption Hospital | 0.13 | 0.03; 0.23 |  |
| Mambolo | -0.05 | -0.16; 0.07 |  |
| UCRC | 0.24 | 0.12; 0.36 |  |
| CVD | 0.11 | -0.01; 0.23 |  |
| Sex |  |  | <0.001 |
| Male | — | — |  |
| Female | 0.13 | 0.08; 0.19 |  |
| CI, confidence interval; CVD, Center for vaccine development (Mali); UCRC, University Clinical Research Center. | | | |

## **S2 Table H.** Parameter estimates evaluating the association between time of prime vaccination and the immune response of IgG anti-EBOV GP_1,2_ at 12 mo in participants vaccinated with rVSV (two doses, with dose 2 at Day 56).

| **Characteristic** | **Beta** | **95% CI** | ***p*-value** |
| --- | --- | --- | --- |
| Population: rVSVΔG-ZEBOV-GP-booster arm |  |  |  |
| Natural splines (time of prime vaccination) |  |  | 0.043 |
| $\beta_{hk}(1)$ | -0.13 | -0.40; 0.14 |  |
| $\beta_{hk}(2)$ | 0.09 | -0.23; 0.42 |  |
| $\beta_{hk}(3)$ | -0.27 | -0.58; 0.03 |  |
| $\beta_{hk}(4)$ | -0.32 | -0.98; 0.34 |  |
| $\beta_{hk}(5)$ | 0.07 | -0.28; 0.42 |  |
| Natural splines (age) |  |  | <0.001 |
| $\beta_{ak}(1)$ | -0.05 | -0.23; 0.12 |  |
| $\beta_{ak}(2)$ | -0.31 | -0.49; -0.12 |  |
| $\beta_{ak}(3)$ | -0.38 | -0.61; -0.14 |  |
| $\beta_{ak}(4)$ | -0.43 | -0.79; -0.07 |  |
| $\beta_{ak}(5)$ | -0.29 | -0.61; 0.03 |  |
| Natural splines (pre-vaccination antibody level) |  |  | 0.001 |
| $\beta_{ank}(1)$ | 0.05 | -0.23; 0.33 |  |
| $\beta_{ank}(2)$ | 0.15 | -0.16; 0.47 |  |
| $\beta_{ank}(3)$ | 0.08 | -0.22; 0.39 |  |
| $\beta_{ank}(4)$ | 0.75 | 0.06; 1.4 |  |
| $\beta_{ank}(5)$ | 1.1 | 0.48; 1.6 |  |
| Study site |  |  | 0.018 |
| Landreah | — | — |  |
| Mafèrinyah | 0.09 | -0.03; 0.21 |  |
| Redemption Hospital | 0.06 | -0.08; 0.19 |  |
| Mambolo | 0.04 | -0.12; 0.19 |  |
| UCRC | 0.30 | 0.13; 0.46 |  |
| CVD | 0.12 | -0.04; 0.29 |  |
| Sex |  |  | <0.001 |
| Male | — | — |  |
| Female | 0.17 | 0.09; 0.24 |  |
| CI, confidence interval; CVD, Center for vaccine development (Mali); UCRC, University Clinical Research Center. | | | |

## **S2 Table I.** Parameter estimates evaluating the association between time of prime vaccination and the immune response of IgG anti-EBOV GP_1,2_ at 12 mo in participants vaccinated with Ad26-MVA.

| **Characteristic** | **Beta** | **95% CI** | ***p*-value** |
| --- | --- | --- | --- |
| Population: Ad26-MVA arm |  |  |  |
| Natural splines (time of prime vaccination) |  |  | 0.14 |
| $\beta_{hk}(1)$ | -0.16 | -0.38; 0.05 |  |
| $\beta_{hk}(2)$ | -0.17 | -0.43; 0.08 |  |
| $\beta_{hk}(3)$ | -0.15 | -0.38; 0.08 |  |
| $\beta_{hk}(4)$ | -0.44 | -0.96; 0.07 |  |
| $\beta_{hk}(5)$ | 0.15 | -0.13; 0.43 |  |
| Natural splines (age) |  |  | <0.001 |
| $\beta_{ak}(1)$ | -0.23 | -0.36; -0.10 |  |
| $\beta_{ak}(2)$ | -0.69 | -0.83; -0.54 |  |
| $\beta_{ak}(3)$ | -0.41 | -0.59; -0.24 |  |
| $\beta_{ak}(4)$ | -0.98 | -1.3; -0.69 |  |
| $\beta_{ak}(5)$ | -0.53 | -0.82; -0.24 |  |
| Natural splines (pre-vaccination antibody level) |  |  | <0.001 |
| $\beta_{ank}(1)$ | 0.08 | -0.19; 0.35 |  |
| $\beta_{ank}(2)$ | 0.02 | -0.28; 0.33 |  |
| $\beta_{ank}(3)$ | 0.19 | -0.03; 0.41 |  |
| $\beta_{ank}(4)$ | 0.70 | 0.05; 1.4 |  |
| $\beta_{ank}(5)$ | 1.2 | 0.82; 1.5 |  |
| Study site |  |  | <0.001 |
| Landreah | — | — |  |
| Mafèrinyah | -0.04 | -0.12; 0.05 |  |
| Redemption Hospital | -0.15 | -0.24; -0.05 |  |
| Mambolo | -0.09 | -0.21; 0.02 |  |
| UCRC | 0.07 | -0.04; 0.18 |  |
| CVD | 0.11 | 0.00; 0.23 |  |
| Sex |  |  | 0.4 |
| Male | — | — |  |
| Female | 0.02 | -0.03; 0.07 |  |
| CI, confidence interval; CVD, Center for vaccine development (Mali); UCRC, University Clinical Research Center. | | | |

## **S2 Table J.** Parameter estimates evaluating the association between time of second vaccination and the immune response of IgG anti-EBOV GP_1,2_ at 12 mo in the participants receiving prime-boost strategy (rVSVbooster or Ad26-MVA arm) in the PREVAC trial.

| **Characteristic** | **Beta** | **95% CI** | ***p*-value** |
| --- | --- | --- | --- |
| Population: rVSV-booster and Ad26-MVA arms |  |  |  |
| Natural splines (time of second vaccination) |  |  | 0.031 |
| $\beta_{hk}(1)$ | -0.19 | -0.38; -0.01 |  |
| $\beta_{hk}(2)$ | 0.03 | -0.19; 0.25 |  |
| $\beta_{hk}(3)$ | -0.15 | -0.32; 0.02 |  |
| $\beta_{hk}(4)$ | -0.19 | -0.63; 0.25 |  |
| $\beta_{hk}(5)$ | -0.05 | -0.29; 0.18 |  |
| Natural splines (age) |  |  | <0.001 |
| $\beta_{ak}(1)$ | -0.18 | -0.30; -0.06 |  |
| $\beta_{ak}(2)$ | -0.56 | -0.69; -0.43 |  |
| $\beta_{ak}(3)$ | -0.42 | -0.58; -0.27 |  |
| $\beta_{ak}(4)$ | -0.86 | -1.1; -0.60 |  |
| $\beta_{ak}(5)$ | -0.47 | -0.73; -0.22 |  |
| Natural splines (pre-vaccination antibody level) |  |  | <0.001 |
| $\beta_{ank}(1)$ | 0.09 | -0.16; 0.34 |  |
| $\beta_{ank}(2)$ | 0.11 | -0.18; 0.39 |  |
| $\beta_{ank}(3)$ | 0.21 | -0.02; 0.43 |  |
| $\beta_{ank}(4)$ | 0.96 | 0.32; 1.6 |  |
| $\beta_{ank}(5)$ | 1.7 | 1.2; 2.1 |  |
| Study site |  |  | <0.001 |
| Landreah | — | — |  |
| Mafèrinyah | 0.00 | -0.08; 0.08 |  |
| Redemption Hospital | -0.01 | -0.09; 0.08 |  |
| Mambolo | -0.05 | -0.13; 0.04 |  |
| UCRC | 0.17 | 0.06; 0.28 |  |
| CVD | 0.17 | 0.07; 0.28 |  |
| Sex |  |  | 0.008 |
| Male | — | — |  |
| Female | 0.06 | 0.02; 0.11 |  |
| CI, confidence interval; CVD, Center for vaccine development (Mali); UCRC, University Clinical Research Center. | | | |

## **S2 Table K.** Parameter estimates evaluating the association between time of second vaccination and the immune response of IgG anti-EBOV GP_1,2_ at 12 mo in participants in the rVSV-booster arm.

| **Characteristic** | **Beta** | **95% CI** | ***p*-value** |
| --- | --- | --- | --- |
| Population: rVSV-booster arm |  |  |  |
| Natural splines (time of second vaccination) |  |  | 0.3 |
| $\beta_{hk}(1)$ | -0.08 | -0.33; 0.17 |  |
| $\beta_{hk}(2)$ | 0.04 | -0.24; 0.32 |  |
| $\beta_{hk}(3)$ | -0.11 | -0.36; 0.14 |  |
| $\beta_{hk}(4)$ | -0.26 | -0.85; 0.32 |  |
| $\beta_{hk}(5)$ | -0.16 | -0.54; 0.21 |  |
| Natural splines (age) |  |  | <0.001 |
| $\beta_{ak}(1)$ | -0.02 | -0.20; 0.15 |  |
| $\beta_{ak}(2)$ | -0.34 | -0.53; -0.16 |  |
| $\beta_{ak}(3)$ | -0.33 | -0.56; -0.10 |  |
| $\beta_{ak}(4)$ | -0.43 | -0.80; -0.07 |  |
| $\beta_{ak}(5)$ | -0.30 | -0.62; 0.02 |  |
| Natural splines (pre-vaccination antibody level) |  |  | <0.001 |
| $\beta_{ank}(1)$ | -0.01 | -0.29; 0.27 |  |
| $\beta_{ank}(2)$ | 0.12 | -0.20; 0.43 |  |
| $\beta_{ank}(3)$ | 0.07 | -0.24; 0.38 |  |
| $\beta_{ank}(4)$ | 0.67 | -0.02; 1.4 |  |
| $\beta_{ank}(5)$ | 1.1 | 0.52; 1.7 |  |
| Study site |  |  | 0.019 |
| Landreah | — | — |  |
| Mafèrinyah | 0.08 | -0.04; 0.21 |  |
| Redemption Hospital | 0.09 | -0.04; 0.22 |  |
| Mambolo | 0.02 | -0.10; 0.14 |  |
| UCRC | 0.31 | 0.14; 0.48 |  |
| CVD | 0.16 | 0.00; 0.33 |  |
| Sex |  |  | <0.001 |
| Male | — | — |  |
| Female | 0.15 | 0.08; 0.22 |  |
| CI, confidence interval; CVD, Center for vaccine development (Mali); UCRC, University Clinical Research Center. | | | |

## **S2 Table L.** Parameter estimates evaluating t the association between time of second vaccination and the immune response of IgG anti-EBOV GP_1,2_ at 12 mo in participants in the Ad26-MVA arm of the PREVAC trial.

| **Characteristic** | **Beta** | **95% CI** | ***p*-value** |
| --- | --- | --- | --- |
| Bras Ad26-MVA |  |  |  |
| Natural splines (time of second vaccination) |  |  | 0.5 |
| $\beta_{hk}(1)$ | -0.14 | -0.34; 0.06 |  |
| $\beta_{hk}(2)$ | -0.01 | -0.25; 0.22 |  |
| $\beta_{hk}(3)$ | -0.08 | -0.27; 0.11 |  |
| $\beta_{hk}(4)$ | -0.08 | -0.55; 0.39 |  |
| $\beta_{hk}(5)$ | 0.09 | -0.16; 0.33 |  |
| Natural splines (age) |  |  | <0.001 |
| $\beta_{ak}(1)$ | -0.24 | -0.37; -0.11 |  |
| $\beta_{ak}(2)$ | -0.69 | -0.84; -0.54 |  |
| $\beta_{ak}(3)$ | -0.42 | -0.60; -0.25 |  |
| $\beta_{ak}(4)$ | -0.98 | -1.3; -0.69 |  |
| $\beta_{ak}(5)$ | -0.52 | -0.82; -0.23 |  |
| Natural splines (pre-vaccination antibody level) |  |  | <0.001 |
| $\beta_{ank}(1)$ | 0.08 | -0.19; 0.35 |  |
| $\beta_{ank}(2)$ | 0.04 | -0.27; 0.35 |  |
| $\beta_{ank}(3)$ | 0.17 | -0.05; 0.39 |  |
| $\beta_{ank}(4)$ | 0.73 | 0.07; 1.4 |  |
| $\beta_{ank}(5)$ | 1.2 | 0.85; 1.5 |  |
| Study site |  |  | <0.001 |
| Landreah | — | — |  |
| Mafèrinyah | -0.05 | -0.14; 0.04 |  |
| Redemption Hospital | -0.11 | -0.20; -0.01 |  |
| Mambolo | -0.10 | -0.19; 0.00 |  |
| UCRC | 0.06 | -0.05; 0.18 |  |
| CVD | 0.12 | 0.01; 0.23 |  |
| Sex |  |  | 0.3 |
| Male | — | — |  |
| Female | 0.03 | -0.03; 0.08 |  |
| CI, confidence interval; CVD, Center for vaccine development (Mali); UCRC, University Clinical Research Center. | | | |


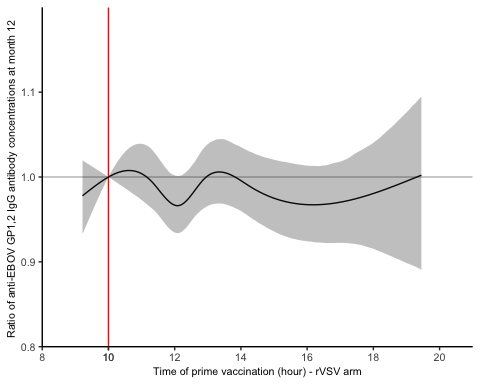


## **S2 Fig D.** Effect of prime vaccination time on antibody response measured at 12 mo post-vaccination in the PREVAC trial in rVSV arm.

The solid line represents the effect of vaccination time on the antibody response. The shaded area corresponds to the 95% confidence interval. The red line at 1000 indicates the reference time against which comparisons are made with other times of the day.


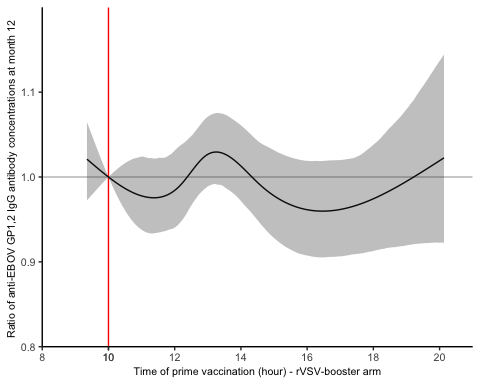


## **S2 Fig E.** Effect of prime vaccination time on antibody response measured at 12 mo post-vaccination in the rVSV-booster arm of the PREVAC trial.

The solid line represents the effect of vaccination time on the antibody response. The shaded area corresponds to the 95% confidence interval. The red line at 1000 indicates the reference time against which comparisons are made with other times of the day.


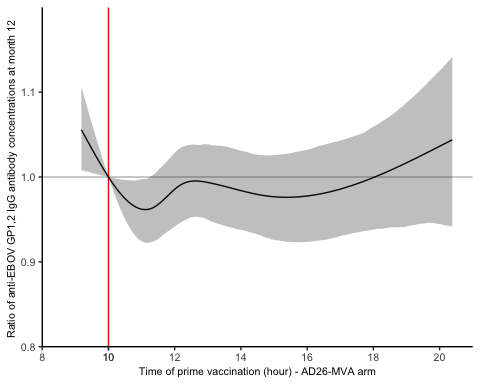


## **S2 Fig F.** Effect of prime vaccination time on antibody response measured at 12 mo post-vaccination in the PREVAC trial in AD26-MVA arm.

The solid line represents the effect of vaccination time on the antibody response. The shaded area corresponds to the 95% confidence interval. The red line at 1000 indicates the reference time against which comparisons are made with other times of the day.


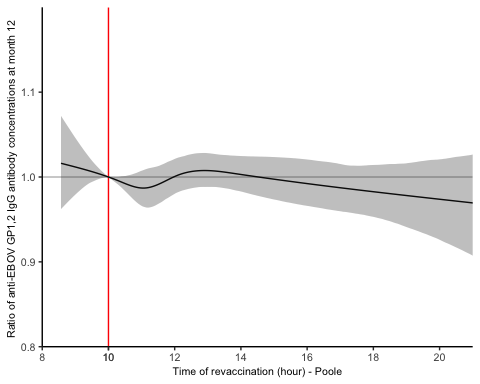


## **S2 Fig G.** Effect of second administration time on antibody response measured at 12 mo post-vaccination in the PREVAC trial in the overall population.

The solid line represents the effect of vaccination time on the antibody response. The shaded area corresponds to the 95% confidence interval. The red line at 1000 indicates the reference time against which comparisons are made with other times of the day.


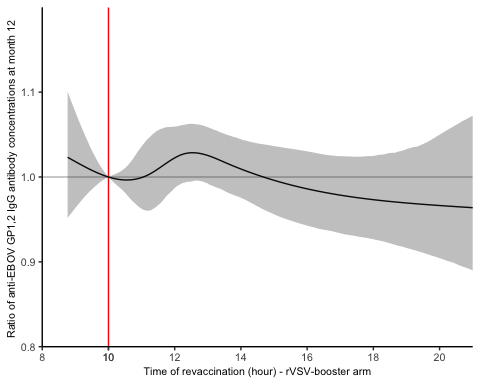


## **S2 Fig H.** Effects of second administration time on antibody response measured at 12 mo post-vaccination in the PREVAC trial in rVSV-booster arm.

The solid line represents the effect of vaccination time on the antibody response. The shaded area corresponds to the 95% confidence interval. The red line at 1000 indicates the reference time against which comparisons are made with other times of the day.


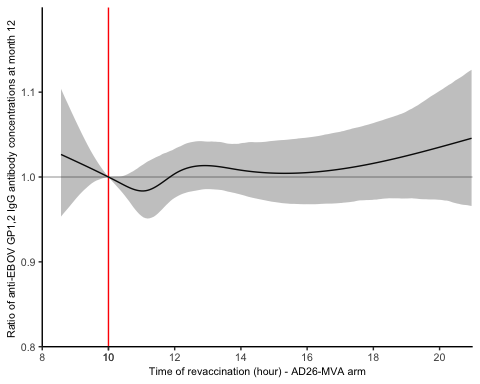


## **S2 Fig I.** Effect of second administration time on antibody response measured at 12 mo post-vaccination in the PREVAC trial in AD26-MVA arm.

The solid line represents the effect of vaccination time on the antibody response. The shaded area corresponds to the 95% confidence interval. The red line at 1000 indicates the reference time against which comparisons are made with other times of the day.


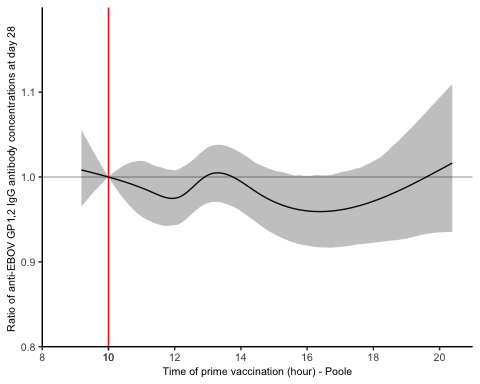


## **S2 Fig J.** Effect of first vaccination time on antibody response measured at day 28 post-vaccination in the PREVAC trial in the overall population.

The solid line represents the effect of vaccination time on the antibody response. The shaded area corresponds to the 95% confidence interval. The red line at 1000 indicates the reference time against which comparisons are made with other times of the day.


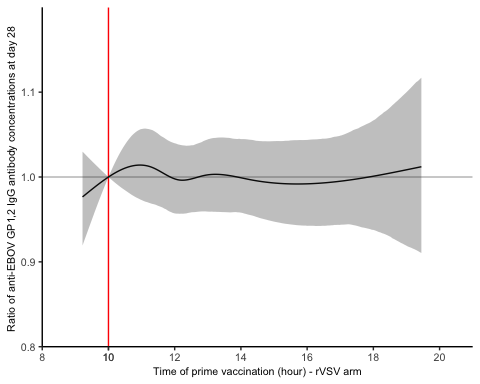


## **S2 Fig K.** Effect of first vaccination time on antibody response measured at day 28 post-vaccination in the PREVAC trial in the pooled rVSV arm.

The solid line represents the effect of vaccination time on the antibody response. The shaded area corresponds to the 95% confidence interval. The red line at 1000 indicates the reference time against which comparisons are made with other times of the day.


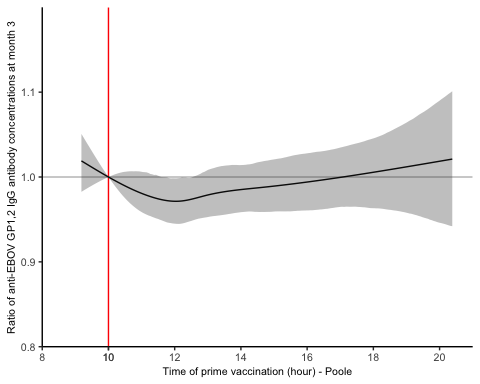


## **S2 Fig L.** Effect of first vaccination time on antibody response measured at 3 mo post-vaccination in the PREVAC trial in the overall population.

The solid line represents the effect of vaccination time on the antibody response. The shaded area corresponds to the 95% confidence interval. The red line at 1000 indicates the reference time against which comparisons are made with other times of the day.


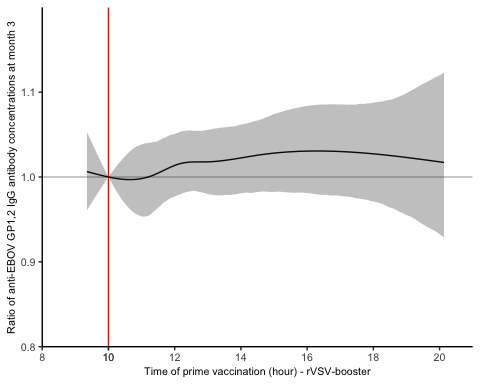


## **S2 Fig M.** Effects of first vaccination time on antibody response measured at 3 mo post-vaccination in the PREVAC trial in rVSV-booster arm.

The solid line represents the effect of vaccination time on the antibody response. The shaded area corresponds to the 95% confidence interval. The red line at 1000 indicates the reference time against which comparisons are made with other times of the day.


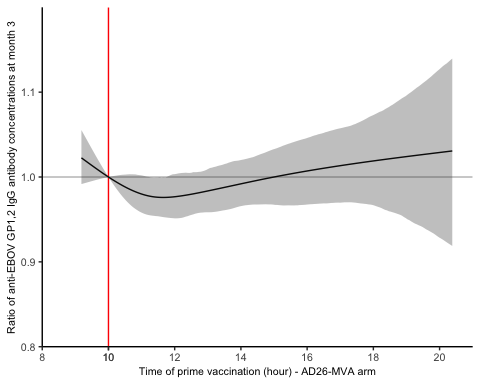


## **S2 Fig N.** Effect of first vaccination time on antibody response measured at 3 mo post-vaccination in the PREVAC trial in AD26-MVA arm.

The solid line represents the effect of vaccination time on the antibody response. The shaded area corresponds to the 95% confidence interval. The red line at 1000 indicates the reference time against which comparisons are made with other times of the day.


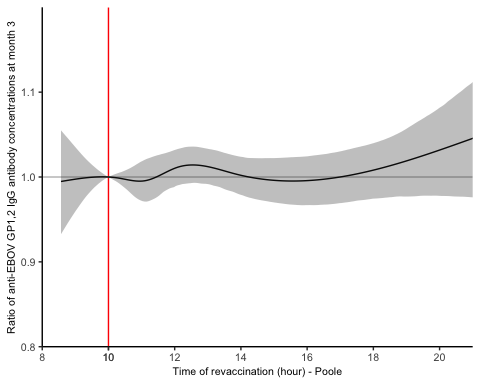


## **S2 Fig O.** Effect of second administration time on antibody response measured at 3 mo post-vaccination in the PREVAC trial in the overall population.

The solid line represents the effect of vaccination time on the antibody response. The shaded area corresponds to the 95% confidence interval. The red line at 1000 indicates the reference time against which comparisons are made with other times of the day.


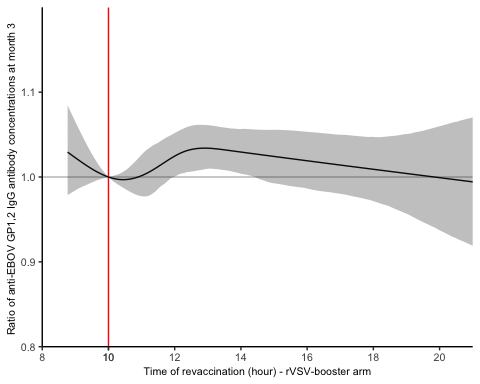


## **S2 Fig P.** Effect of second administration time on antibody response measured at 3 mo post-vaccination in the PREVAC trial in rVSV-booster arm.

The solid line represents the effect of vaccination time on the antibody response. The shaded area corresponds to the 95% confidence interval. The red line at 1000 indicates the reference time against which comparisons are made with other times of the day.


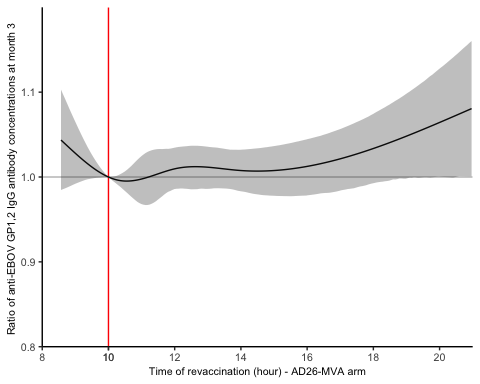


## **S2 Fig Q.** Effect of second administration time on antibody response measured at 3 mo post-vaccination in the PREVAC trial in AD26-MVA arm.

The solid line represents the effect of vaccination time on the antibody response. The shaded area corresponds to the 95% confidence interval. The red line at 1000 indicates the reference time against which comparisons are made with other times of the day.
