# Supplementary material for: Effect of the time of day for vaccination on the immune response to Ebola Virus Disease vaccines: A modeling study from PREVAC randomized trial
Source: PLoS Negl Trop Dis. 2026 Jan 30;20(1):e0013950. doi: 10.1371/journal.pntd.0013950 (PMC12893655; doi:10.1371/journal.pntd.0013950)
Supplement: S3 Text — (DOCX) [file pntd.0013950.s003.docx]

# Supplementary 3: FANG assay


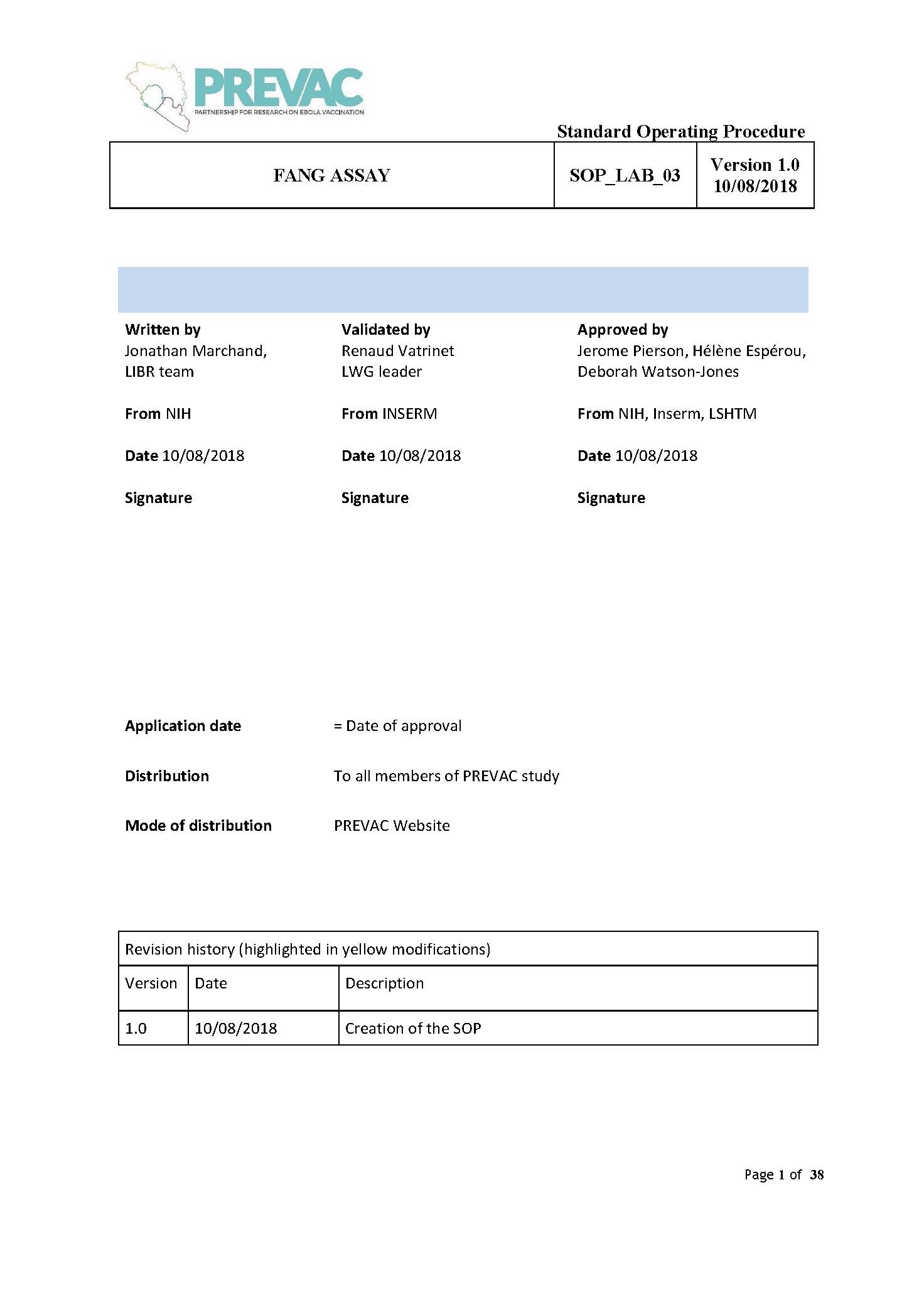


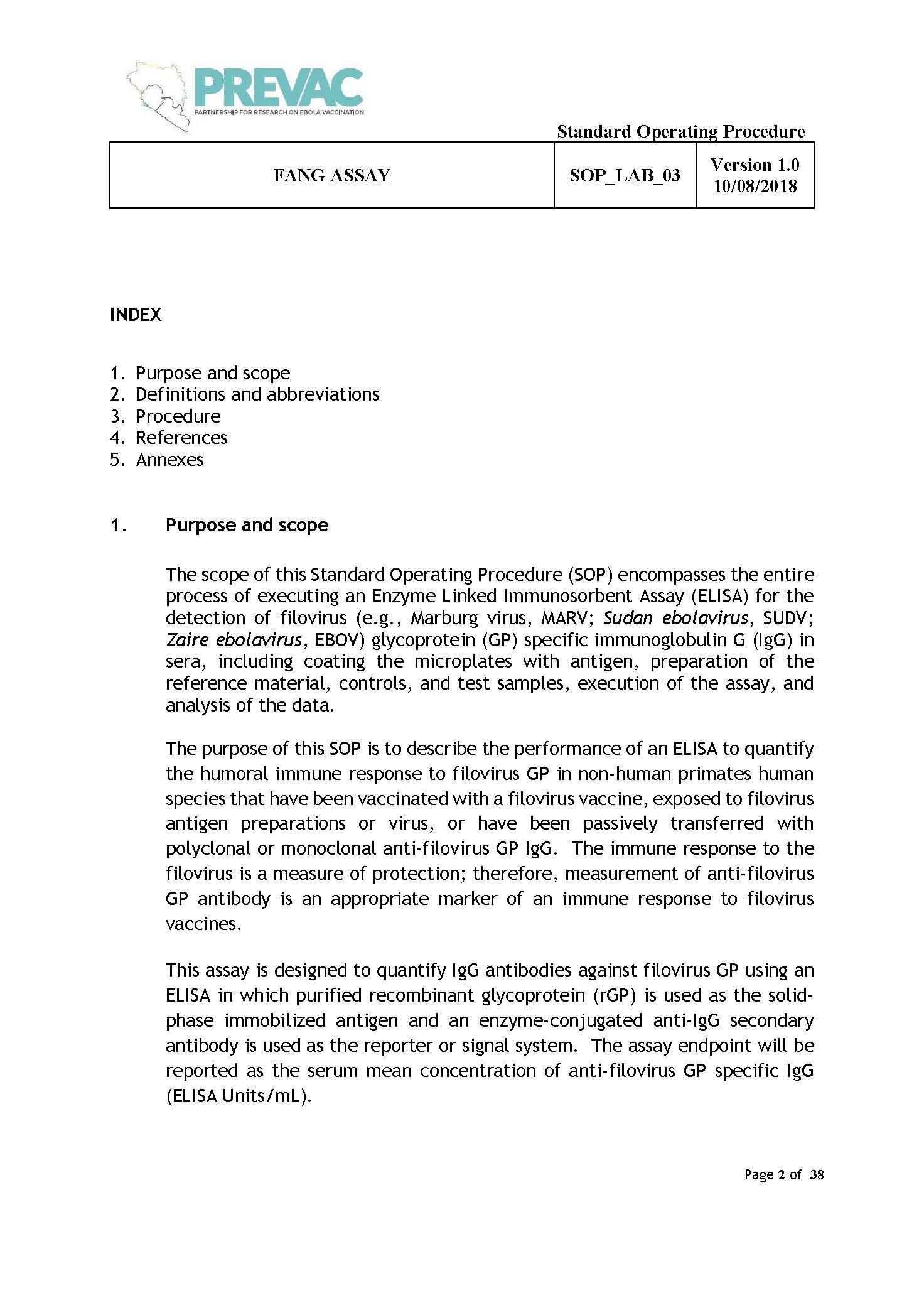

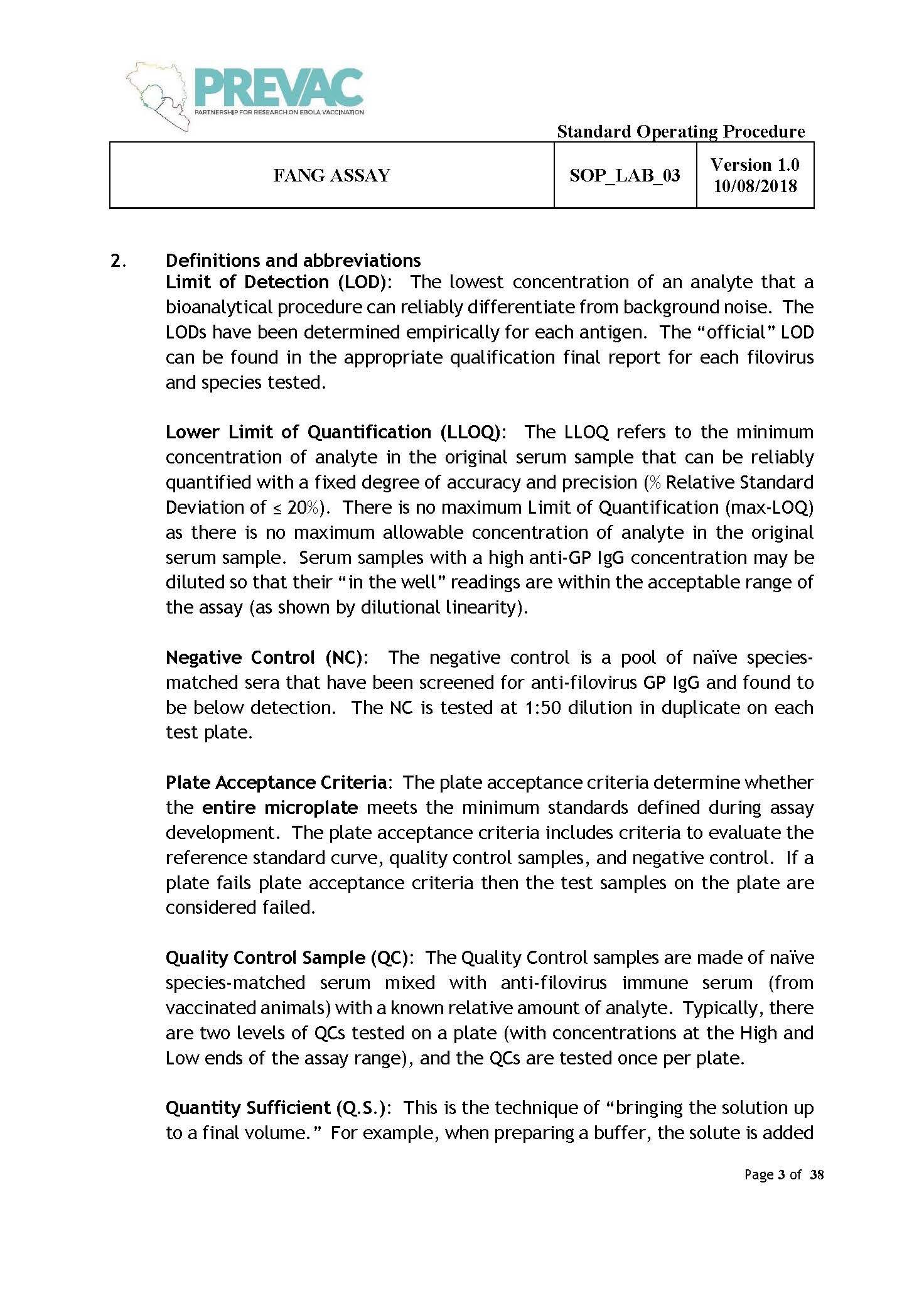

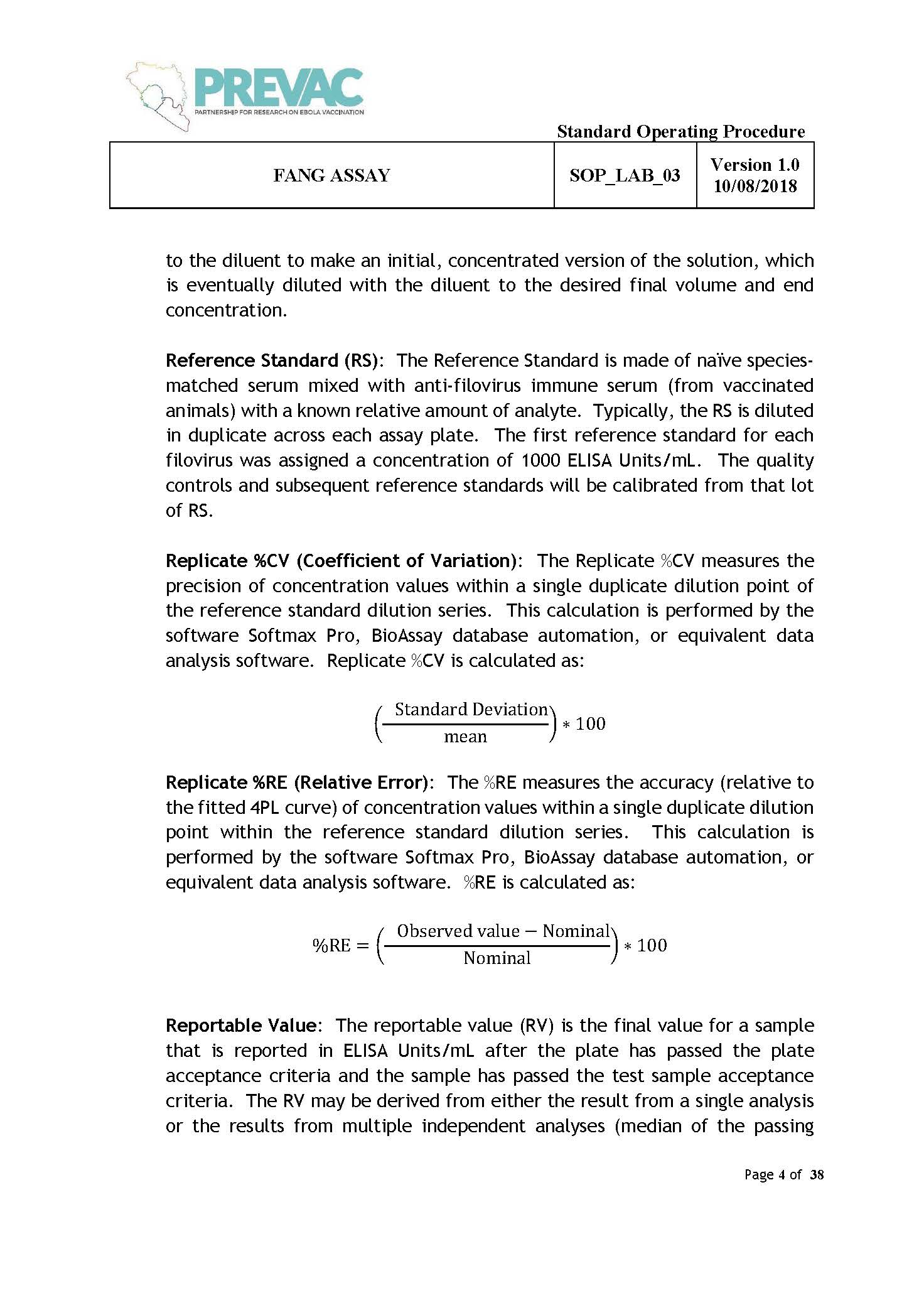

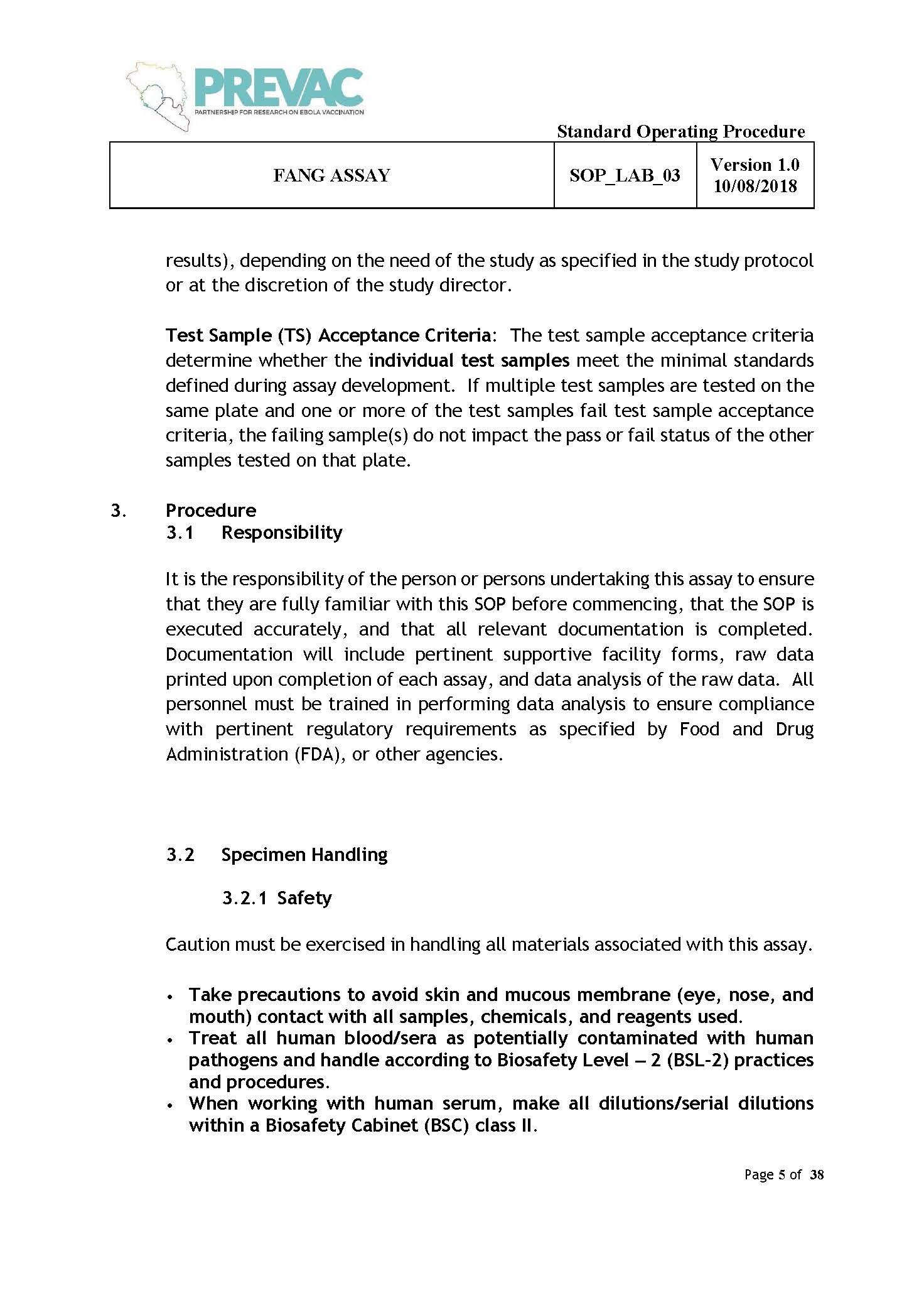

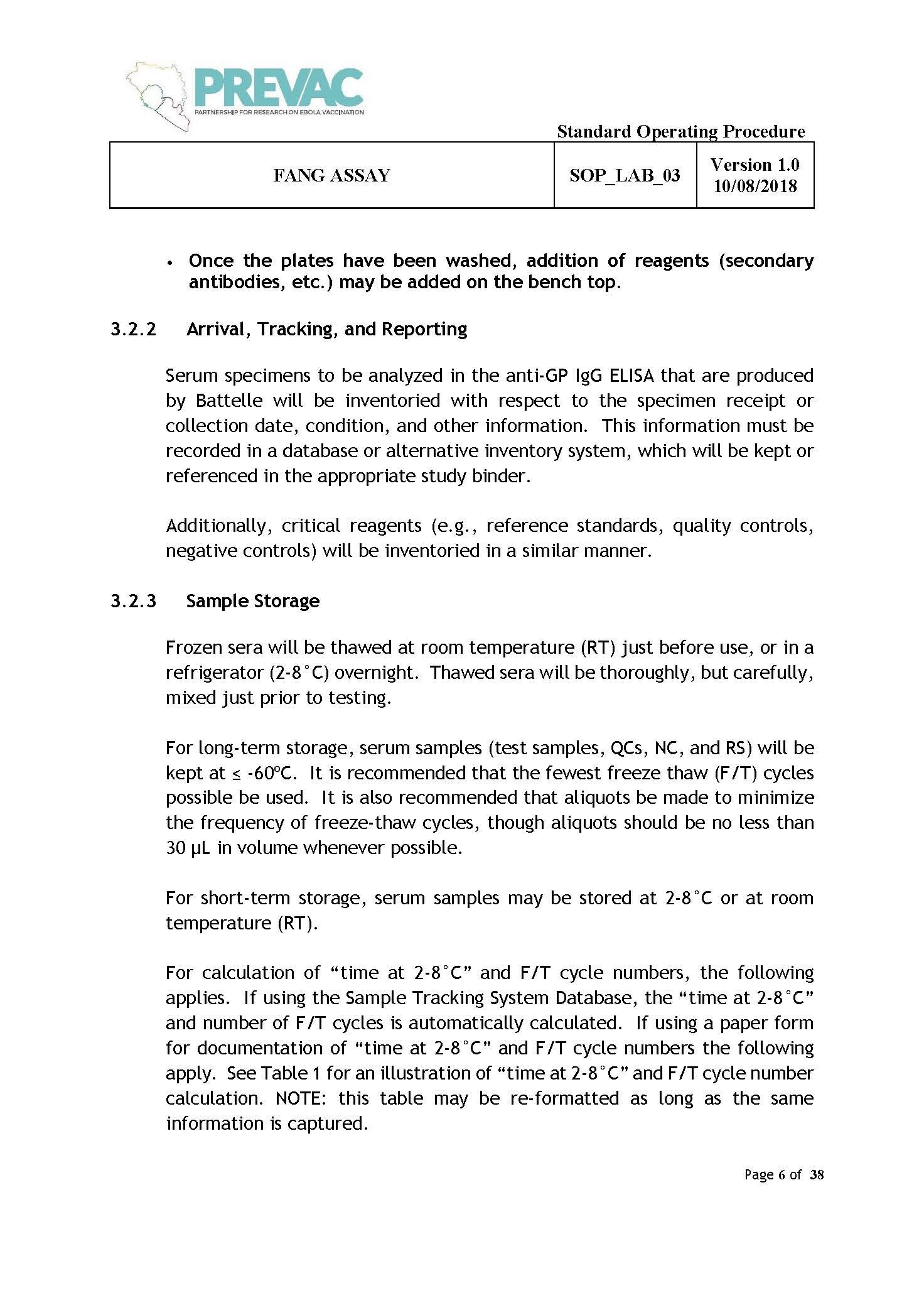

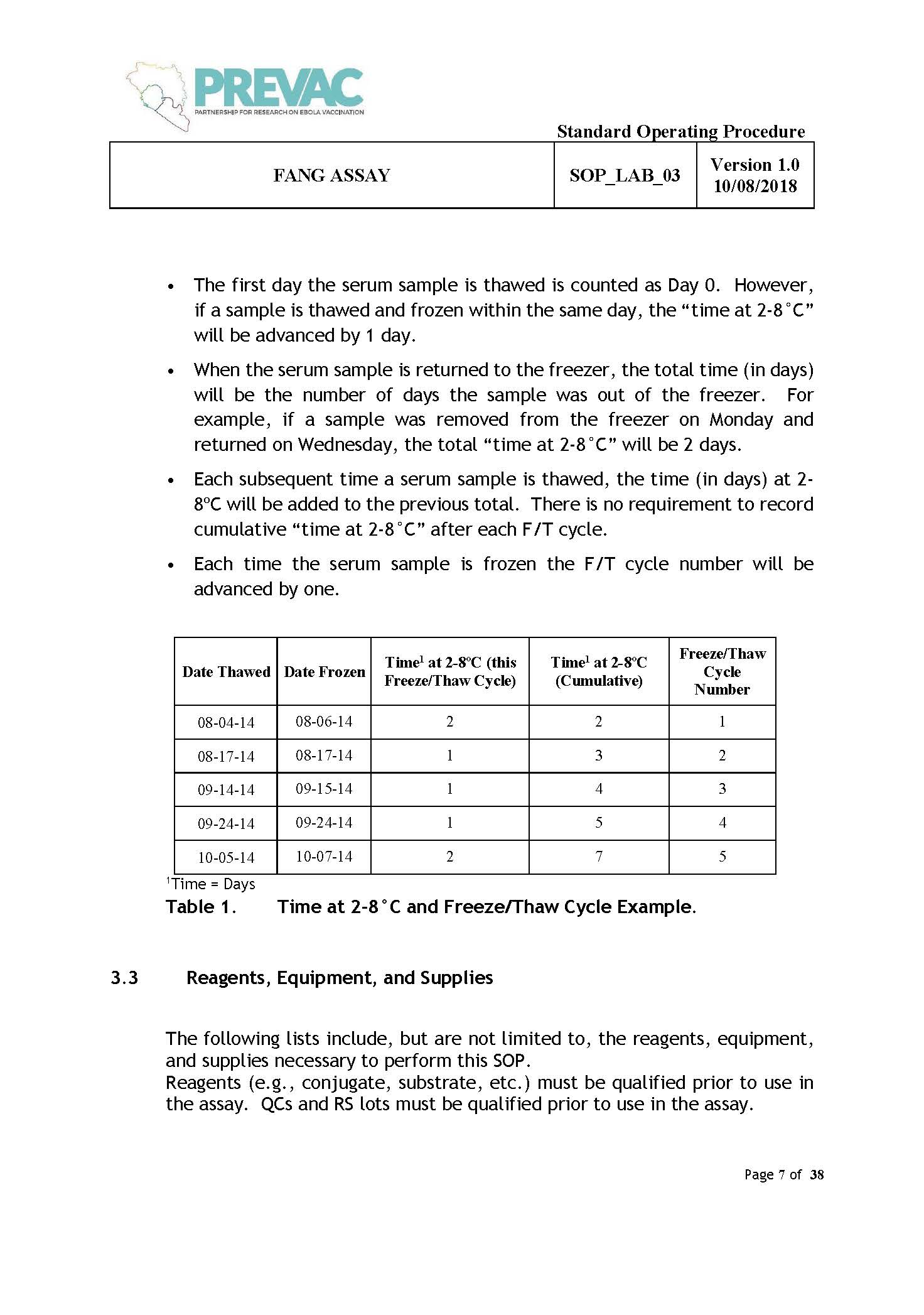

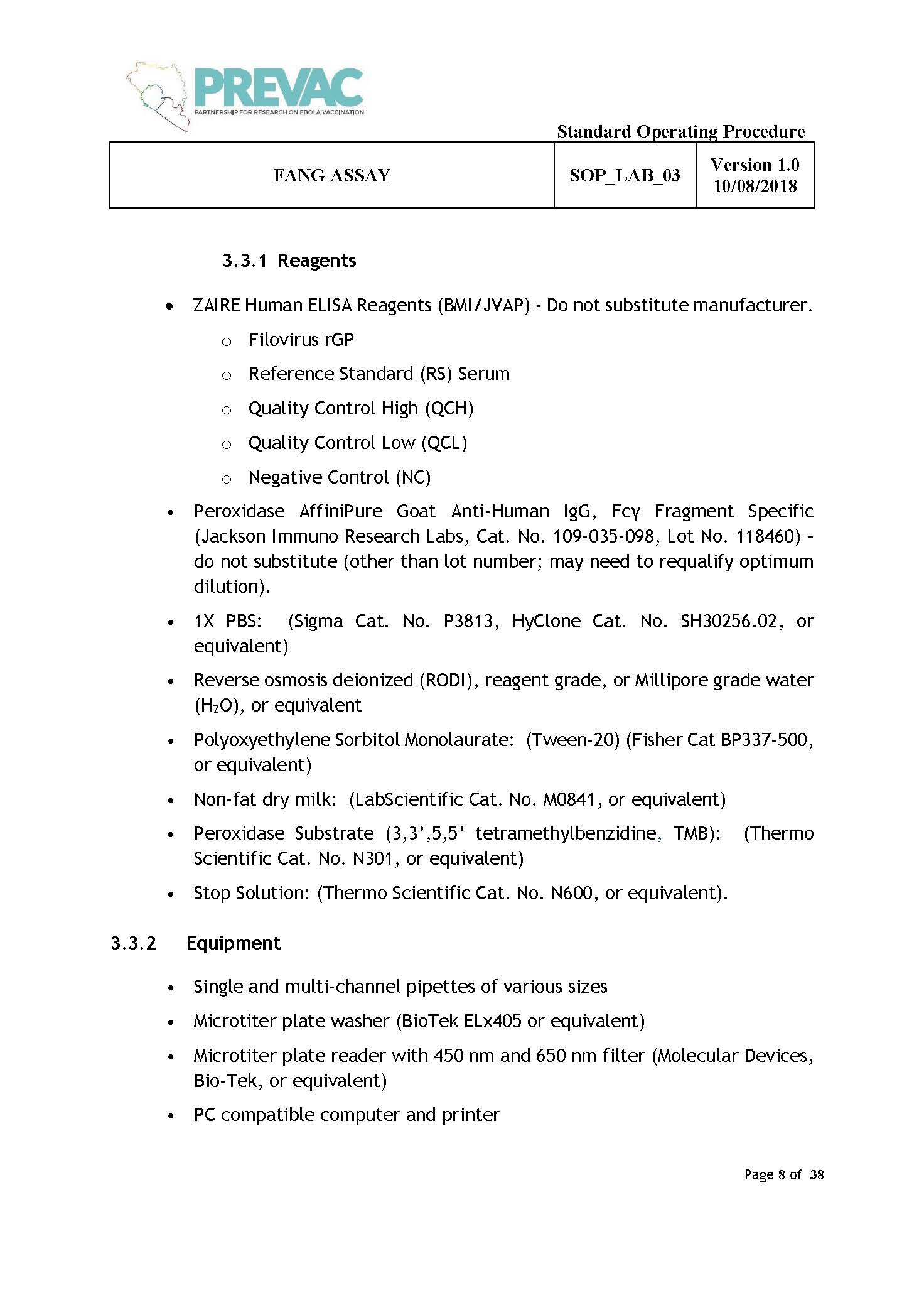

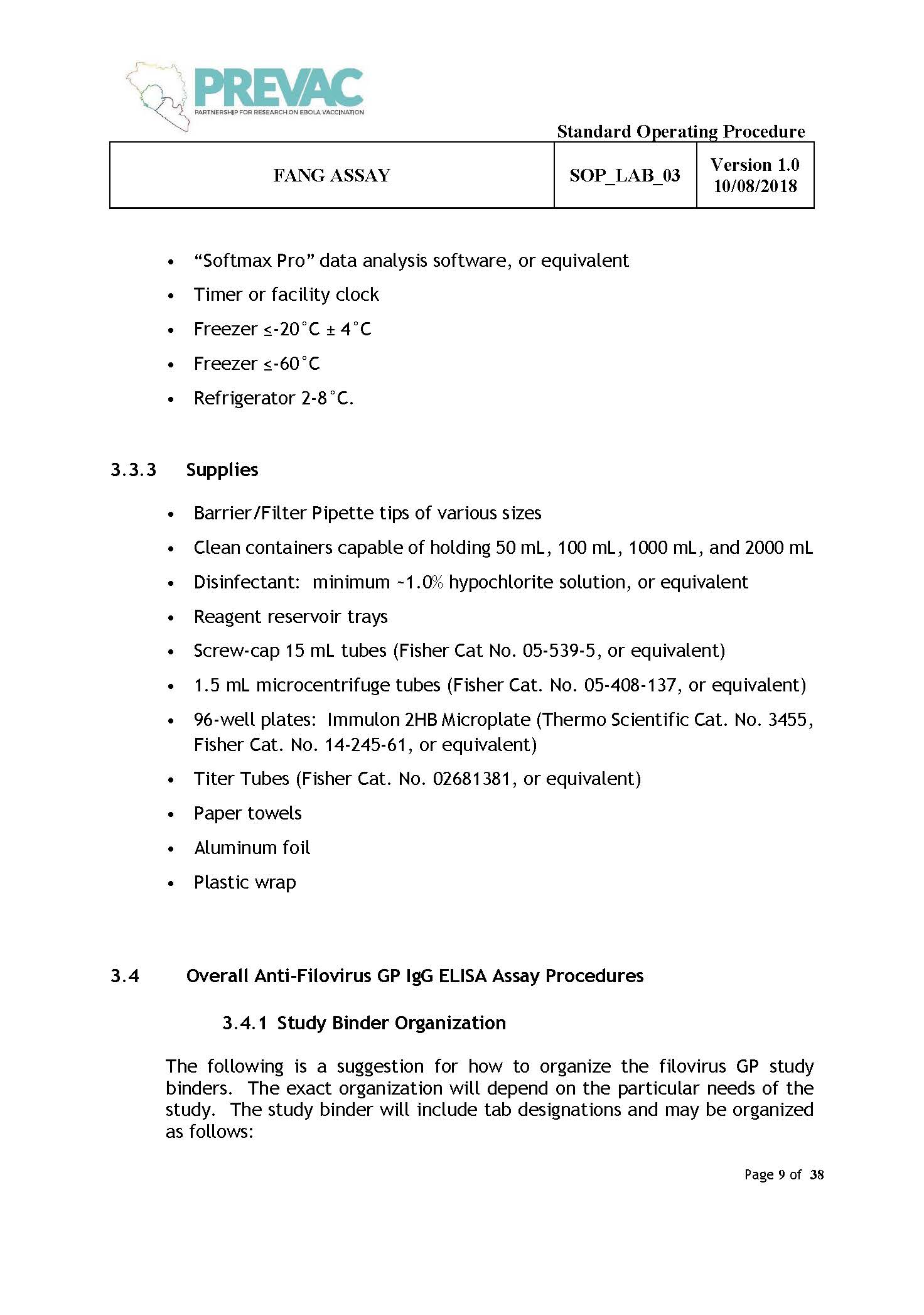

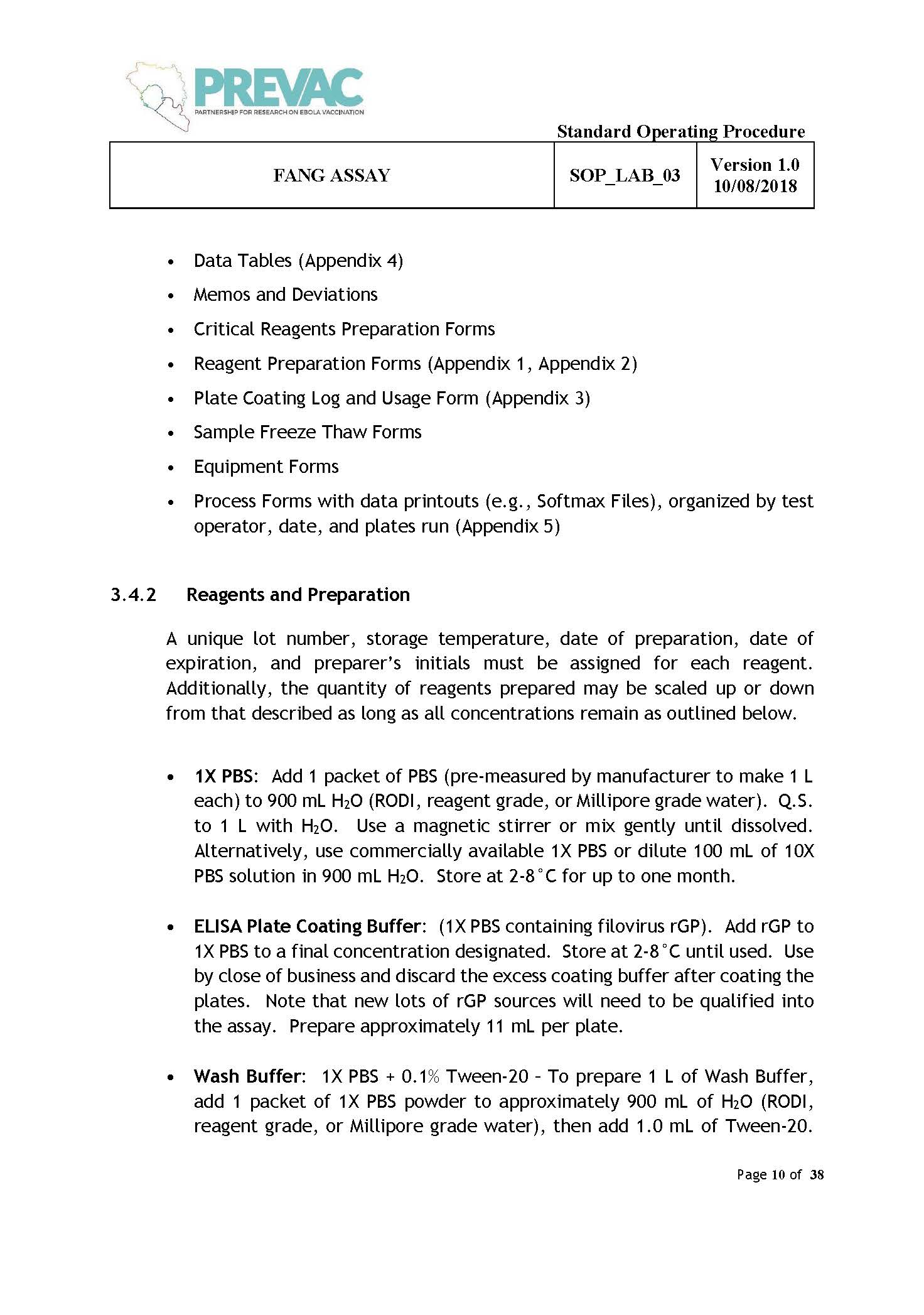

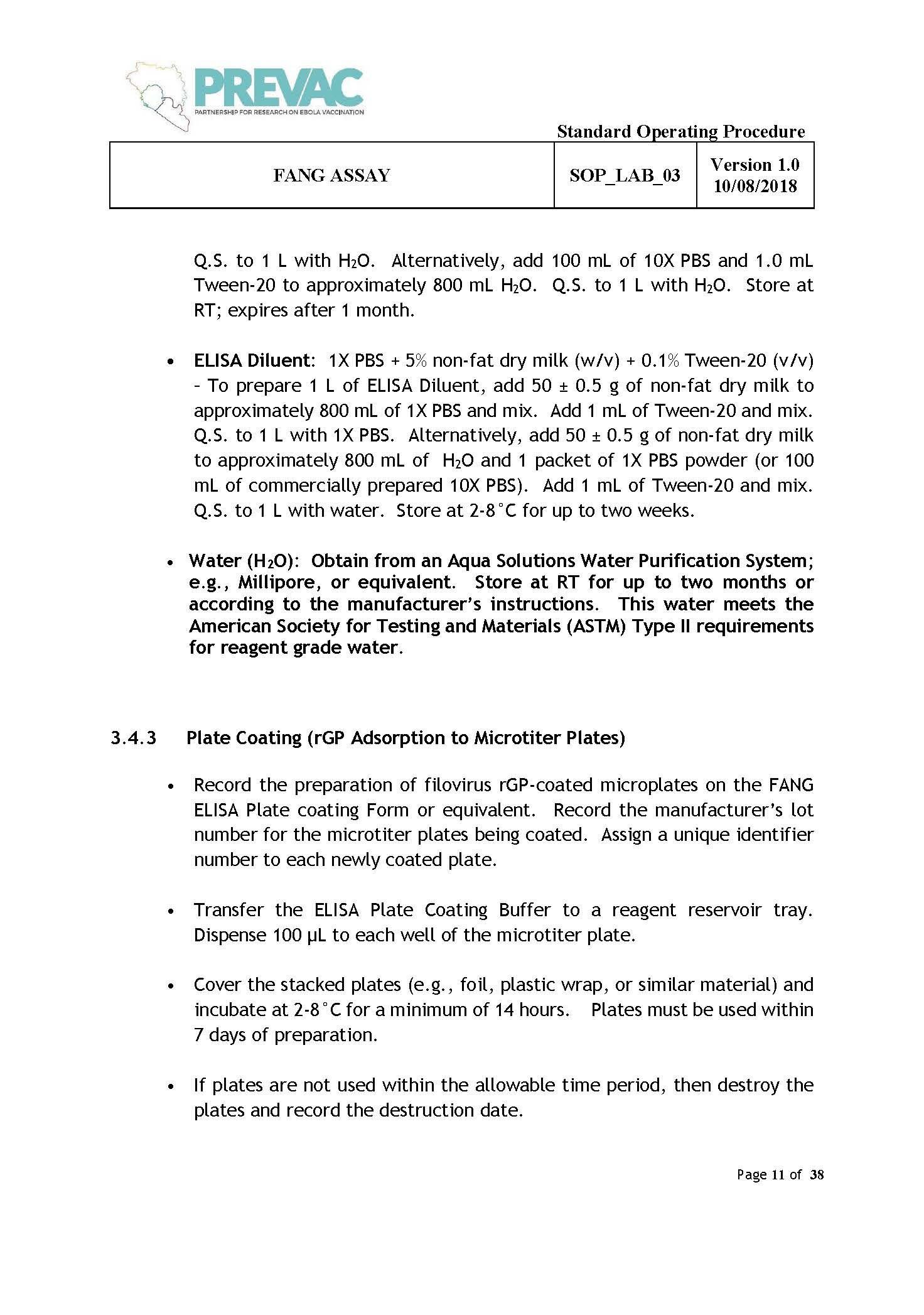

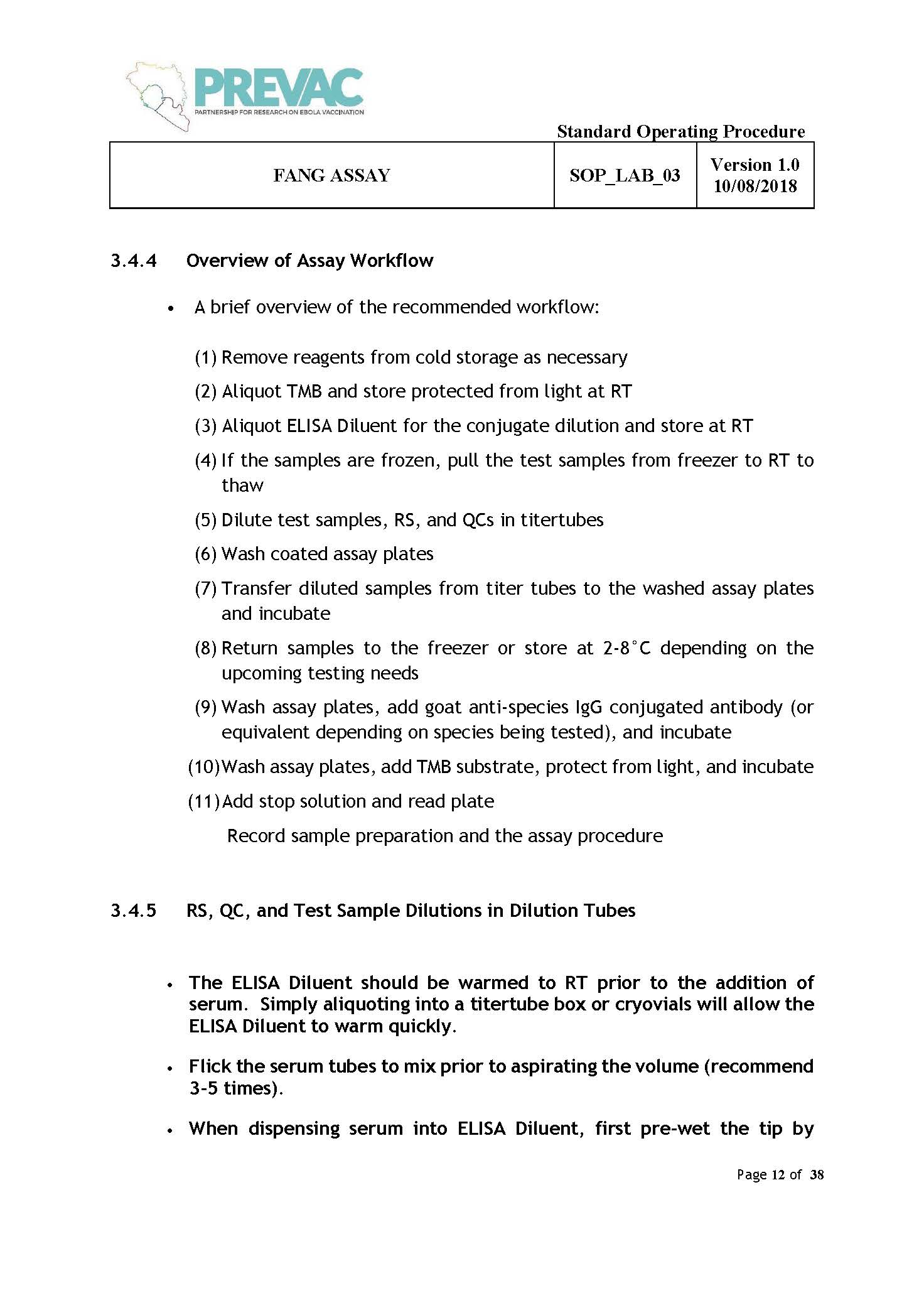

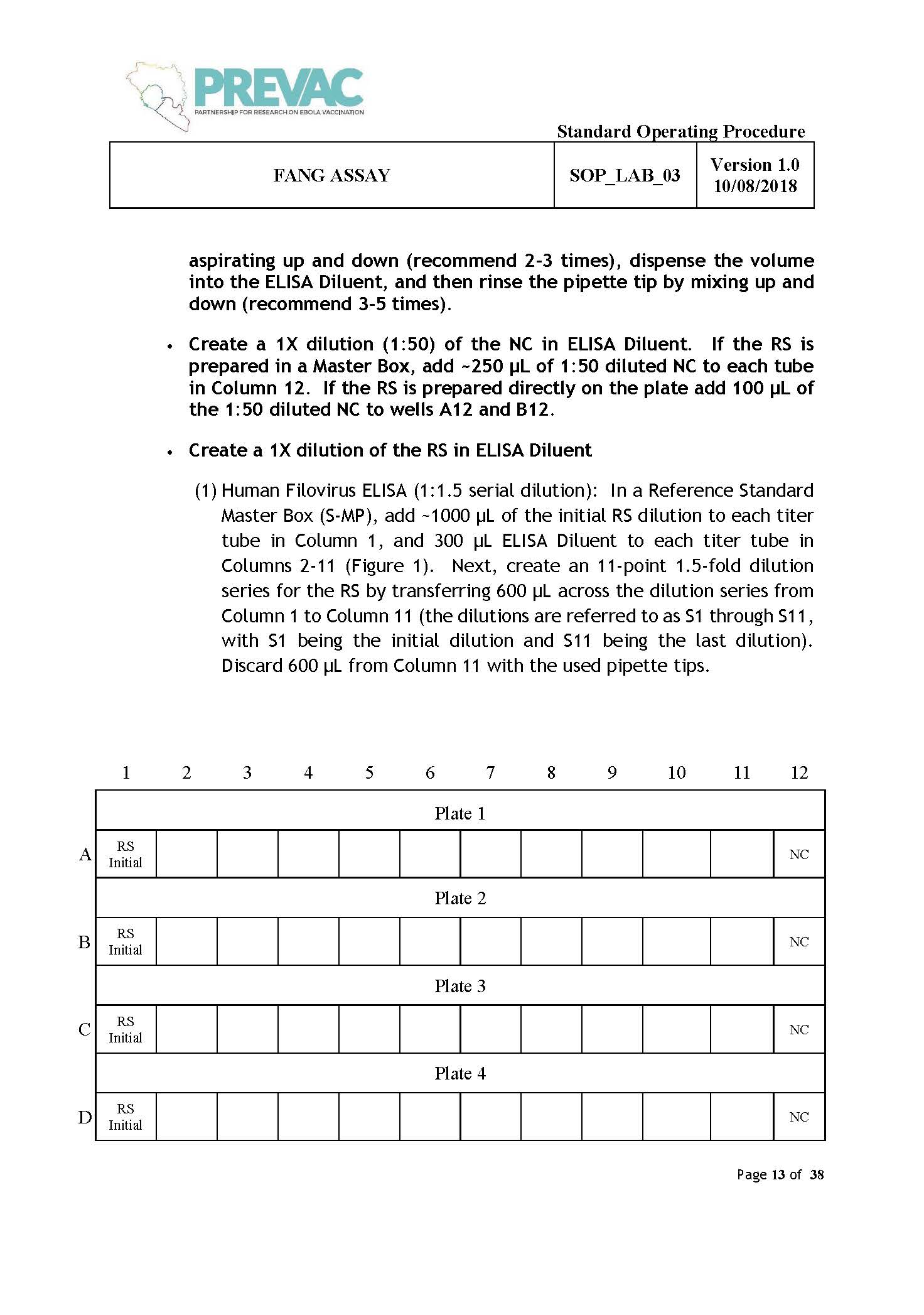

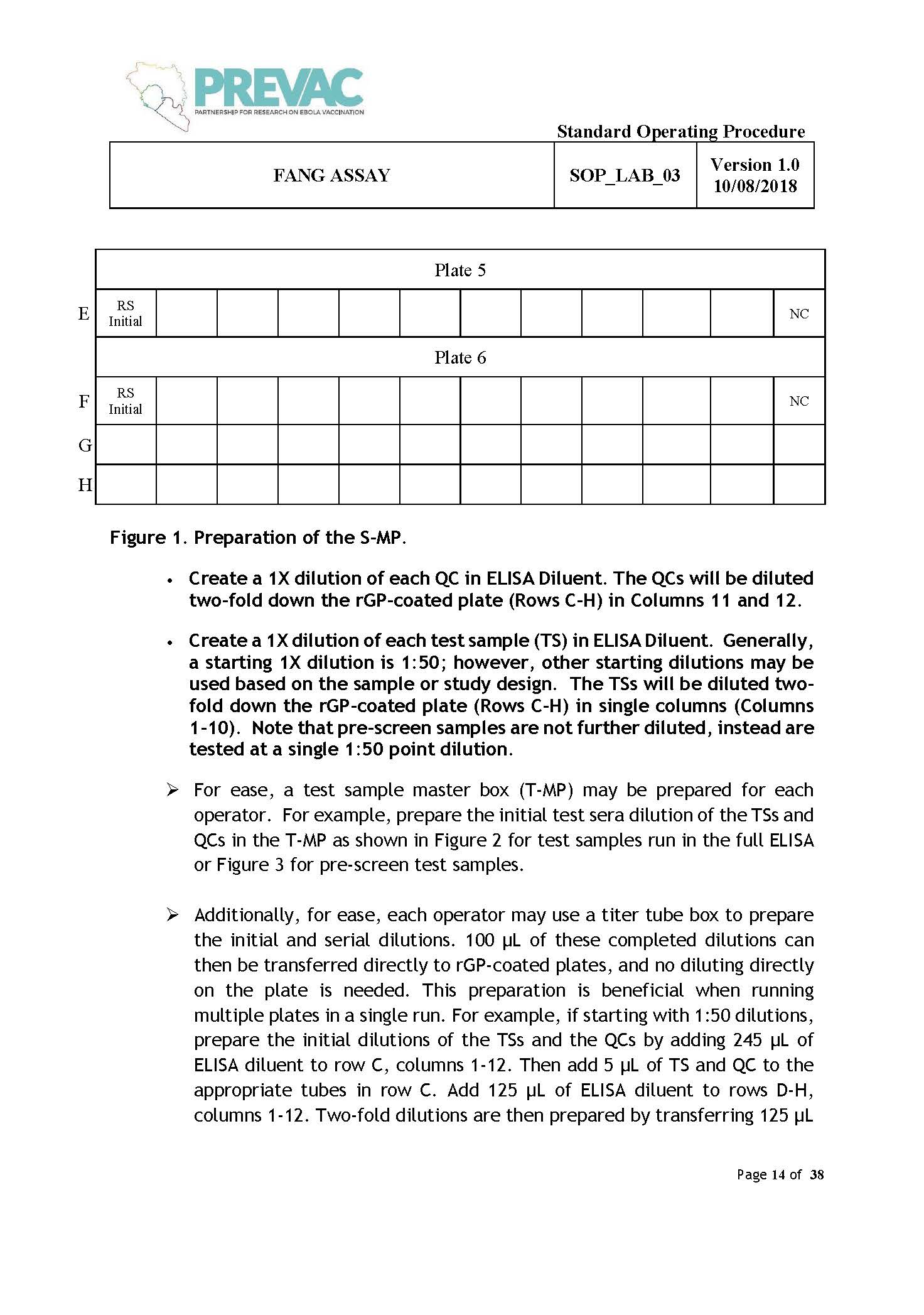

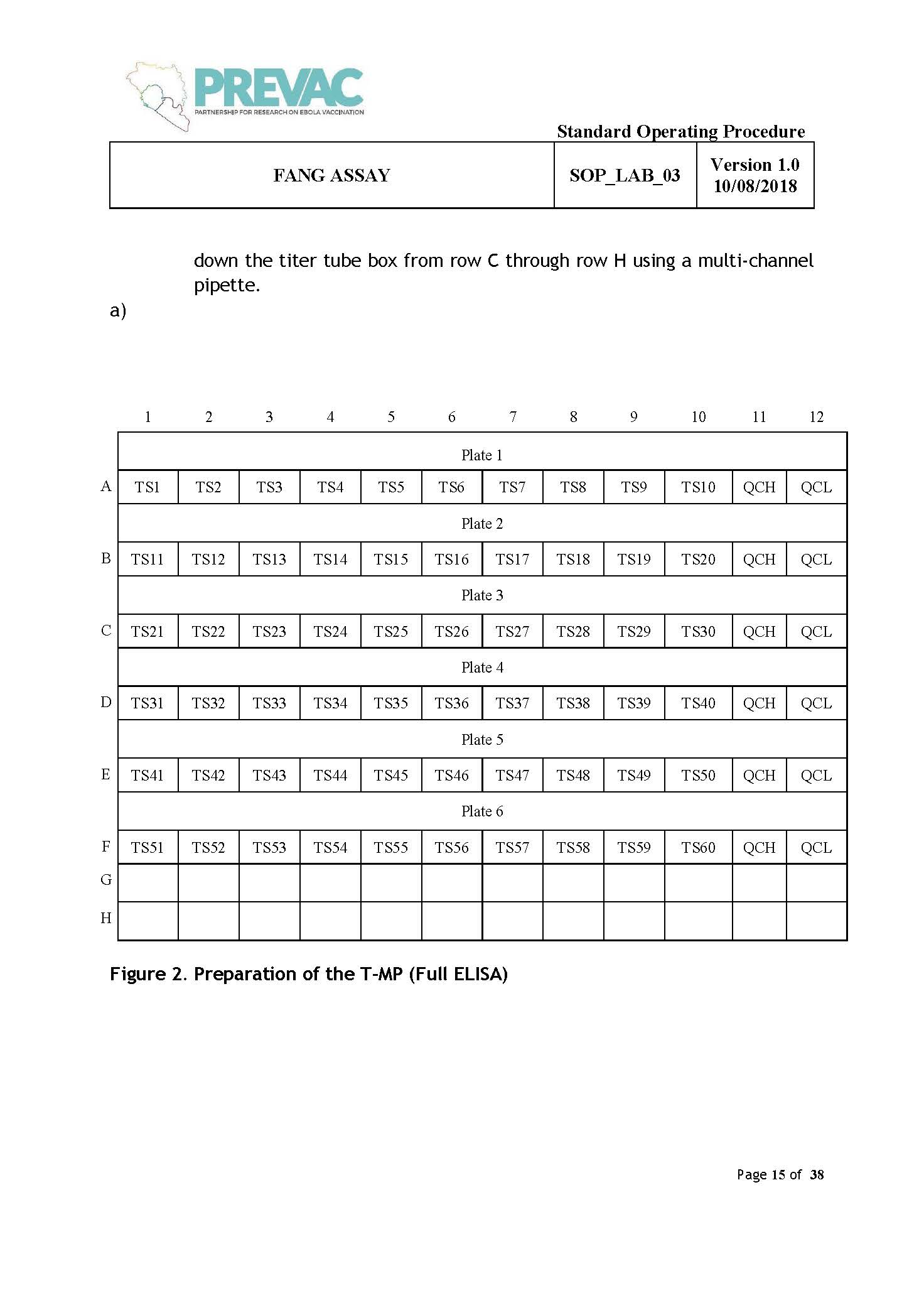

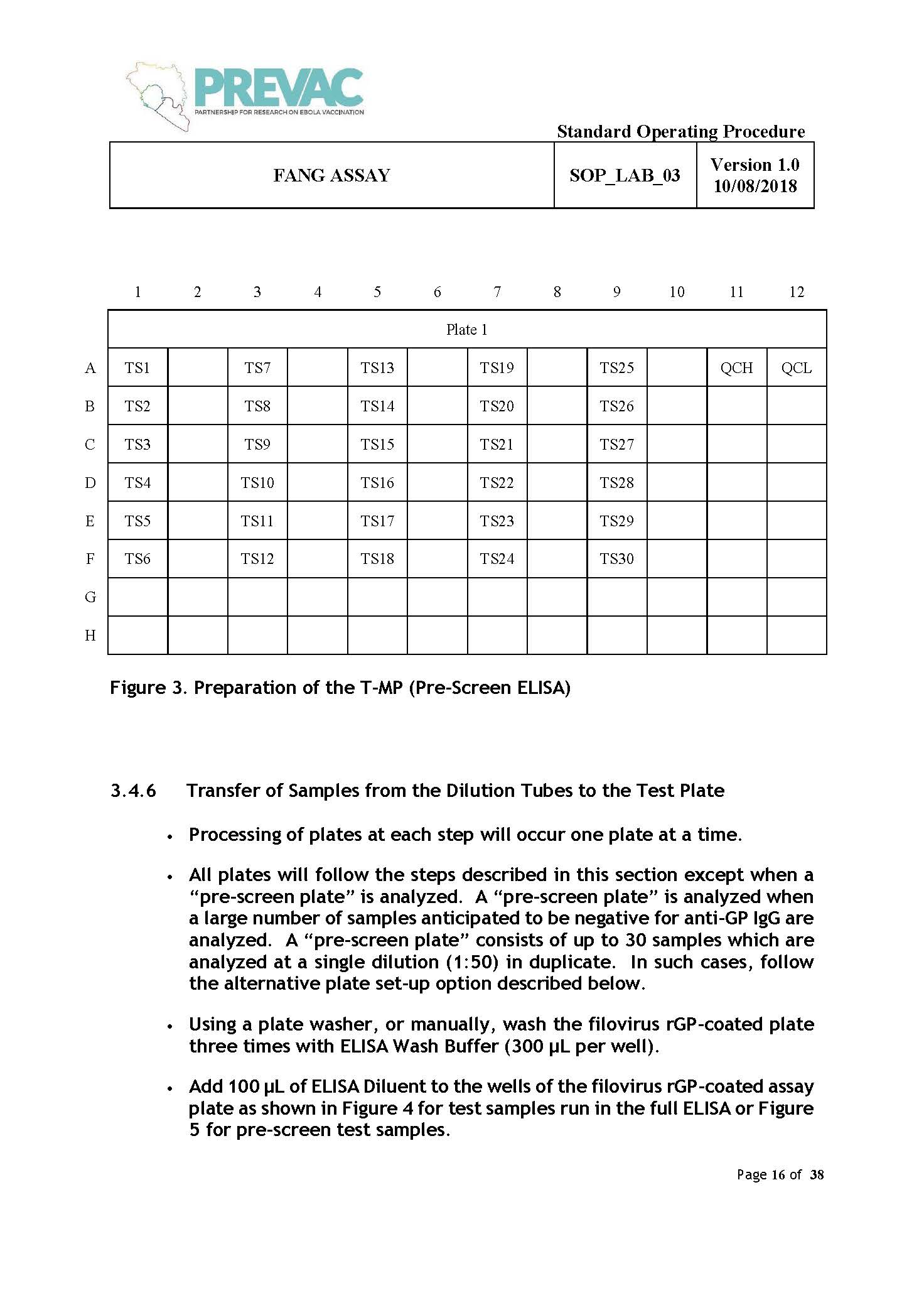

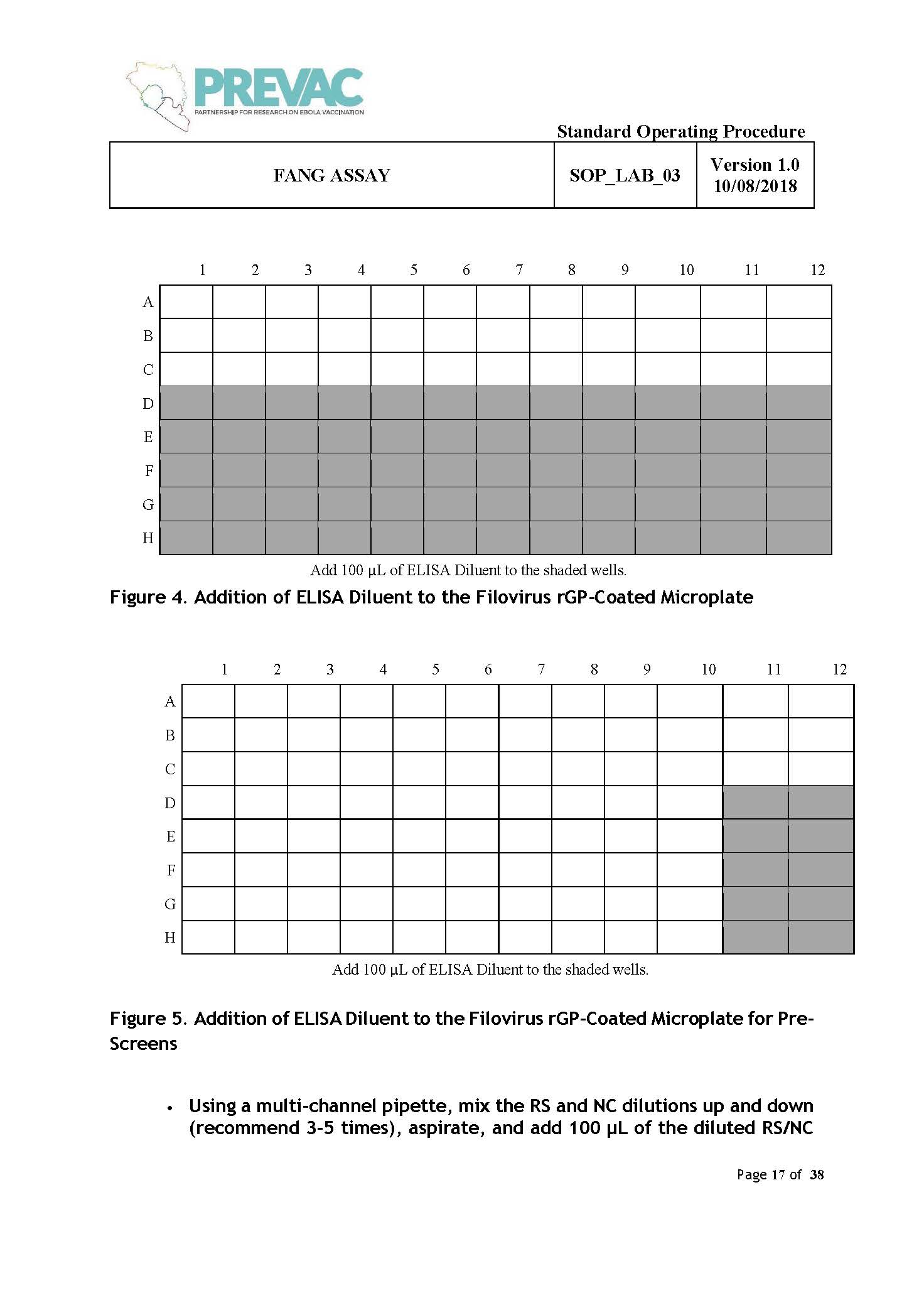

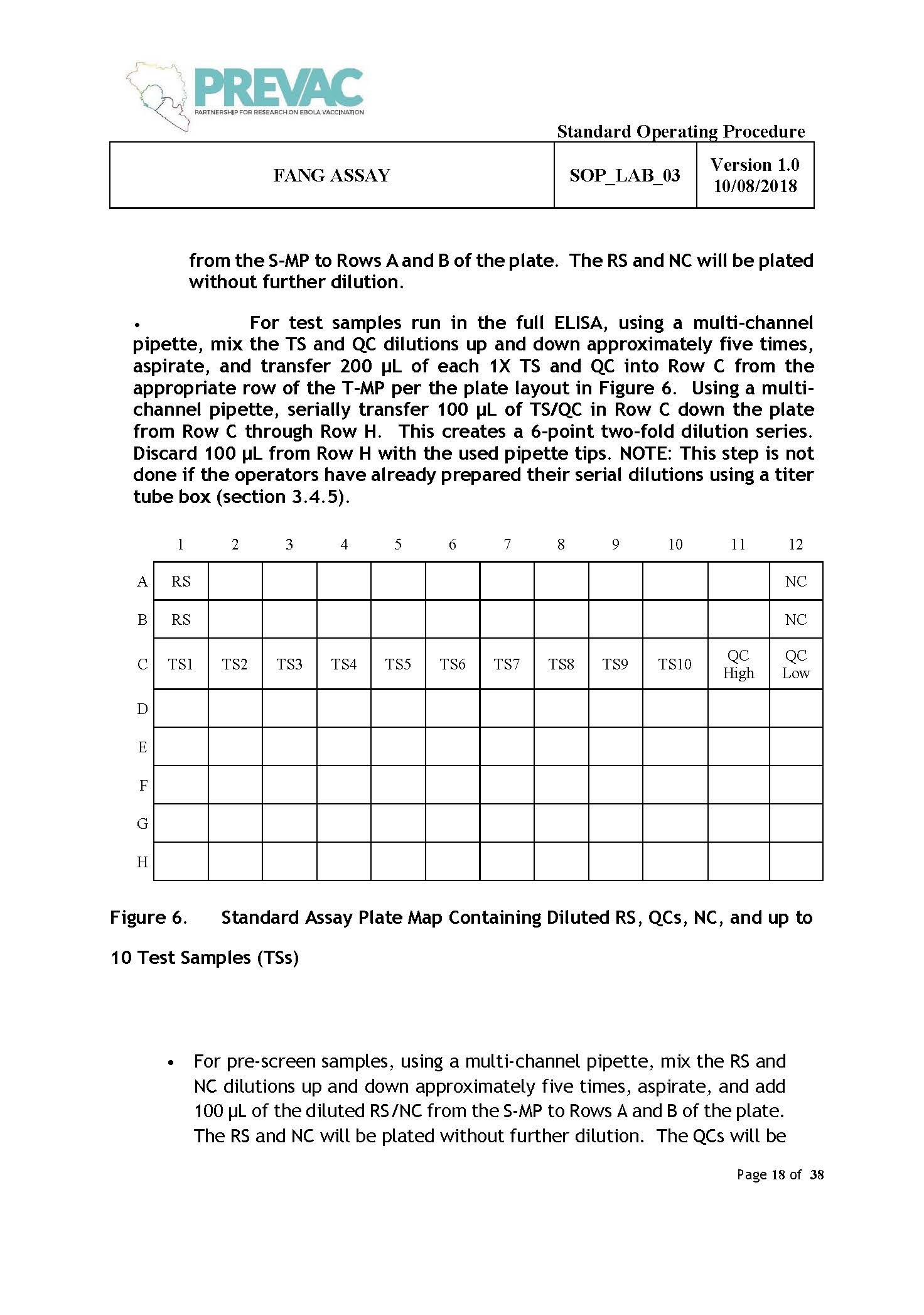

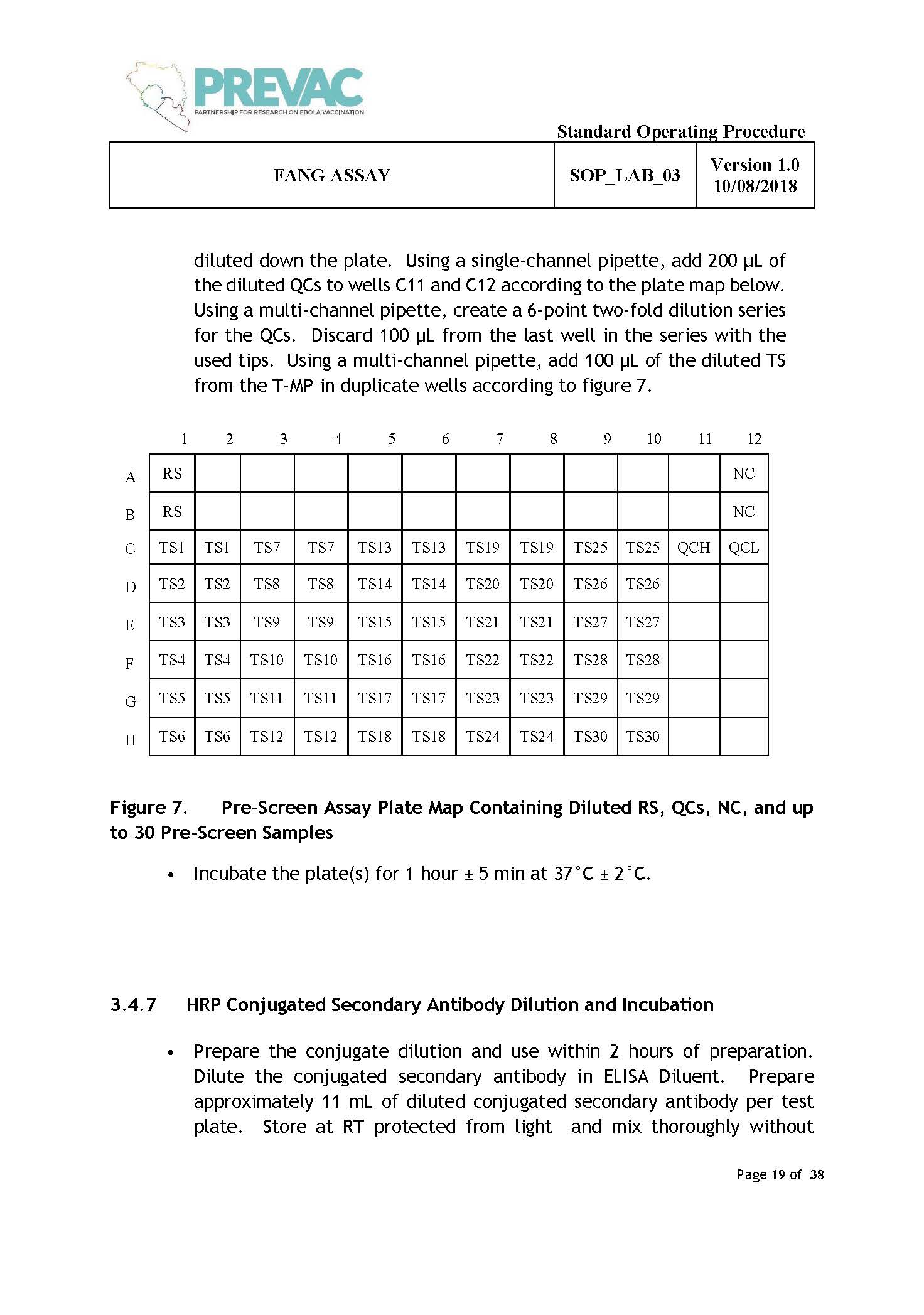

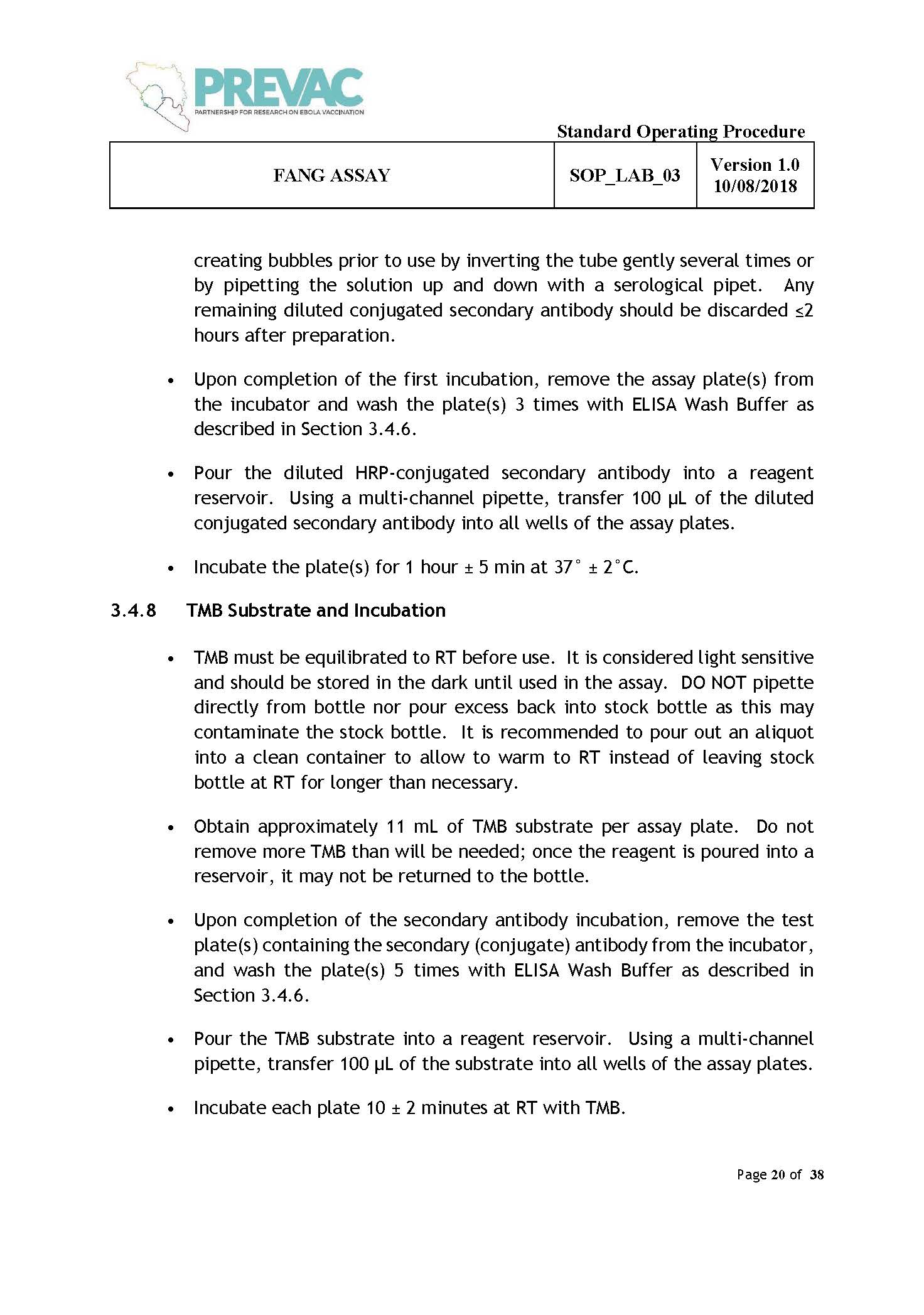

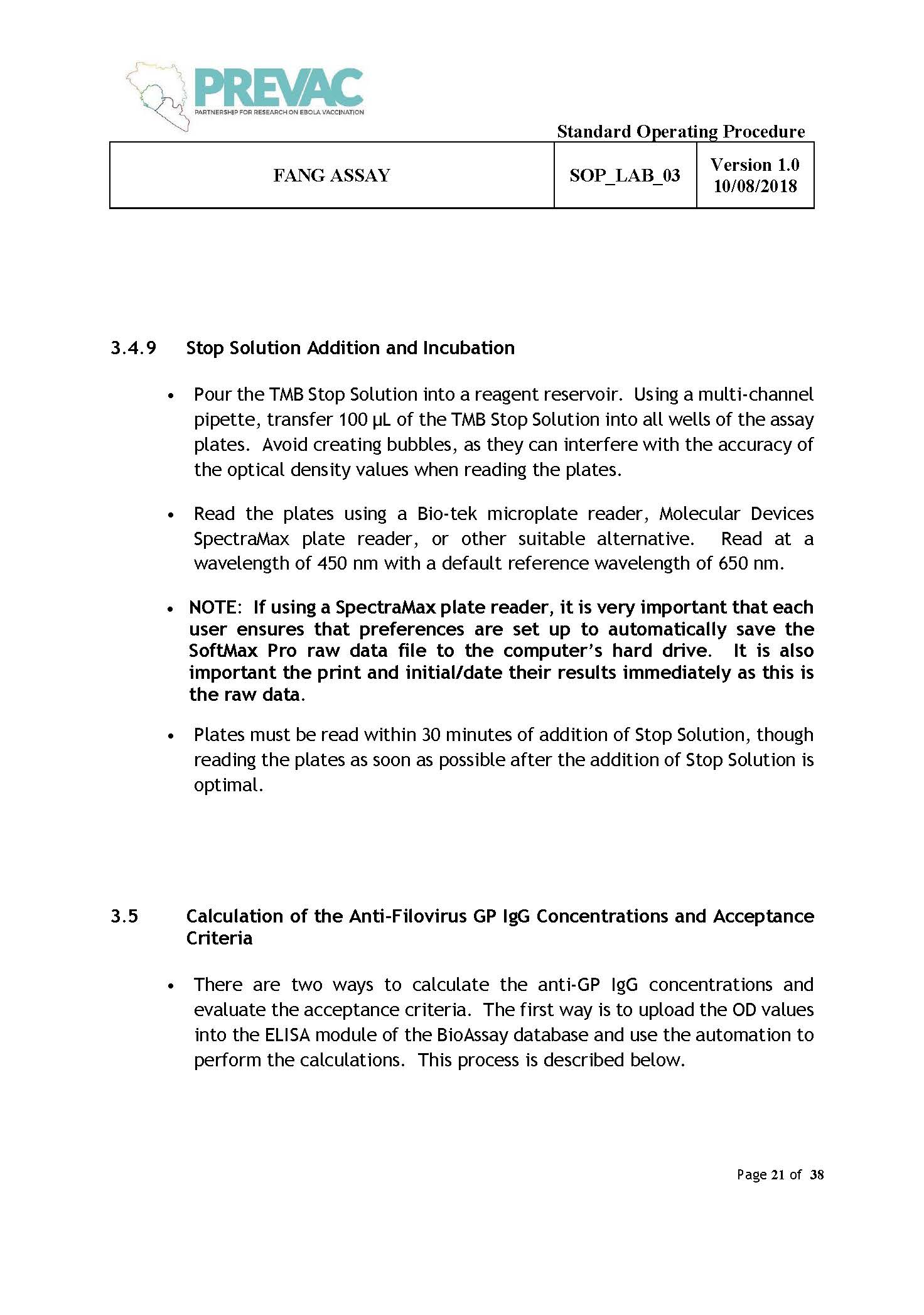

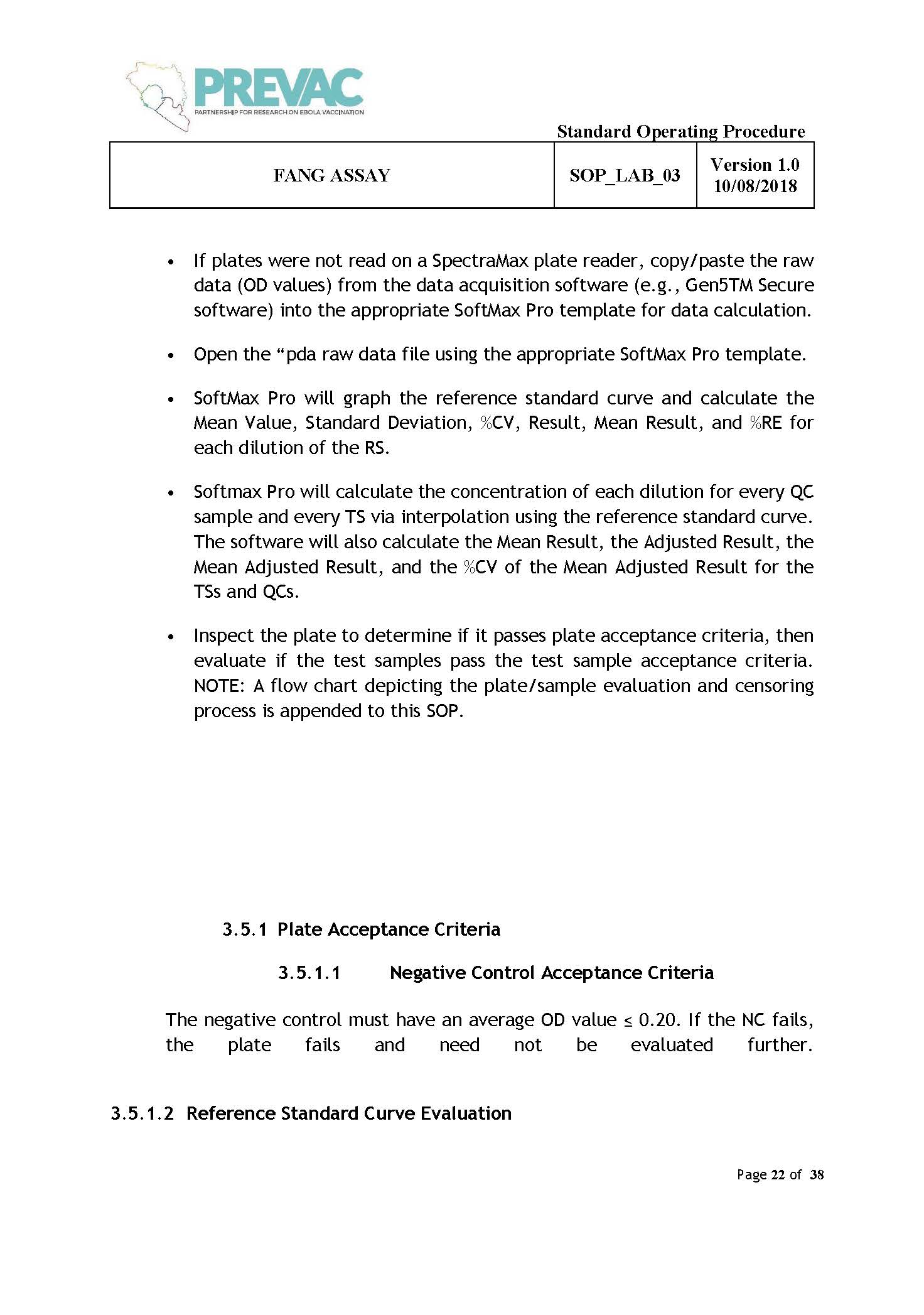

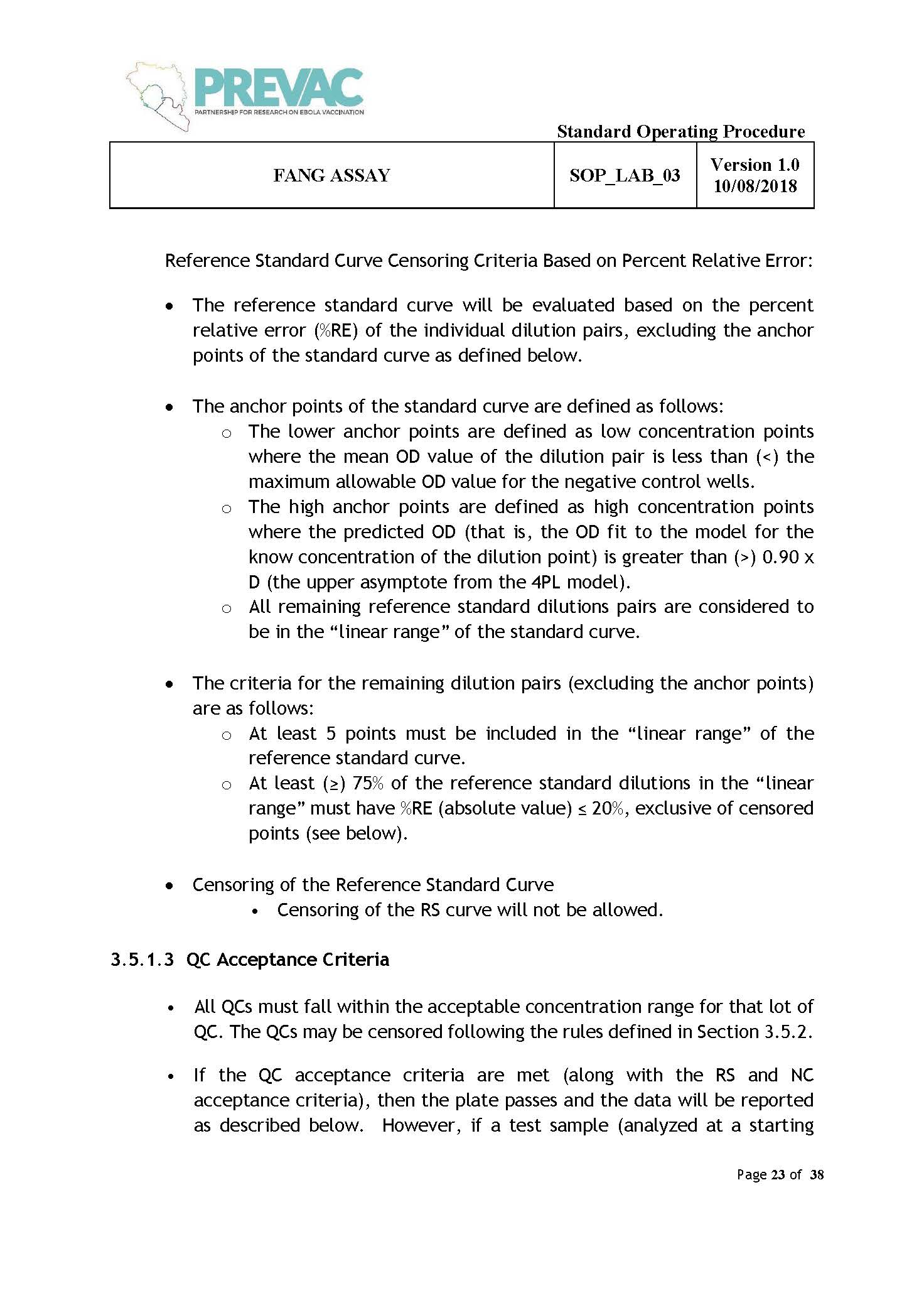

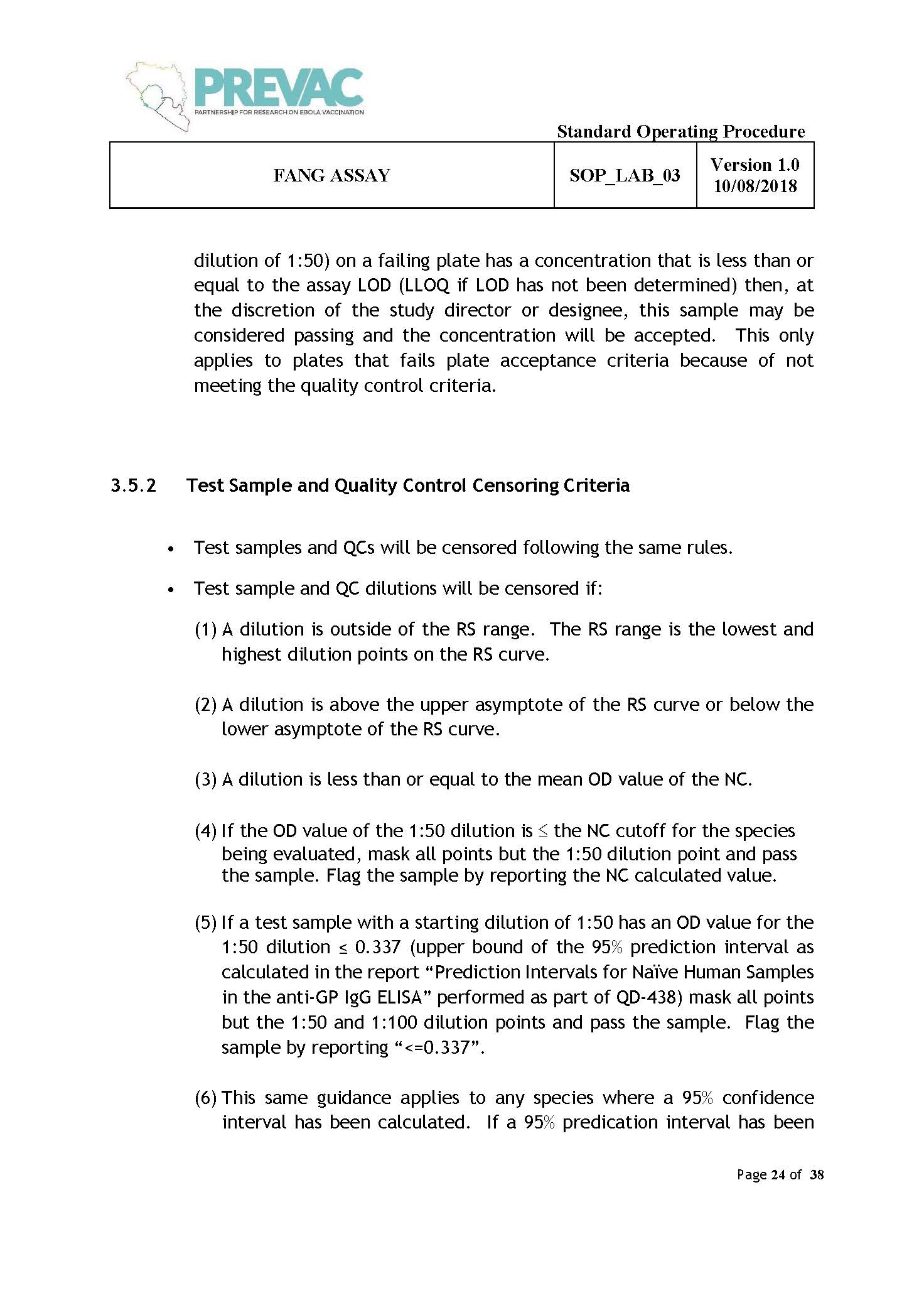

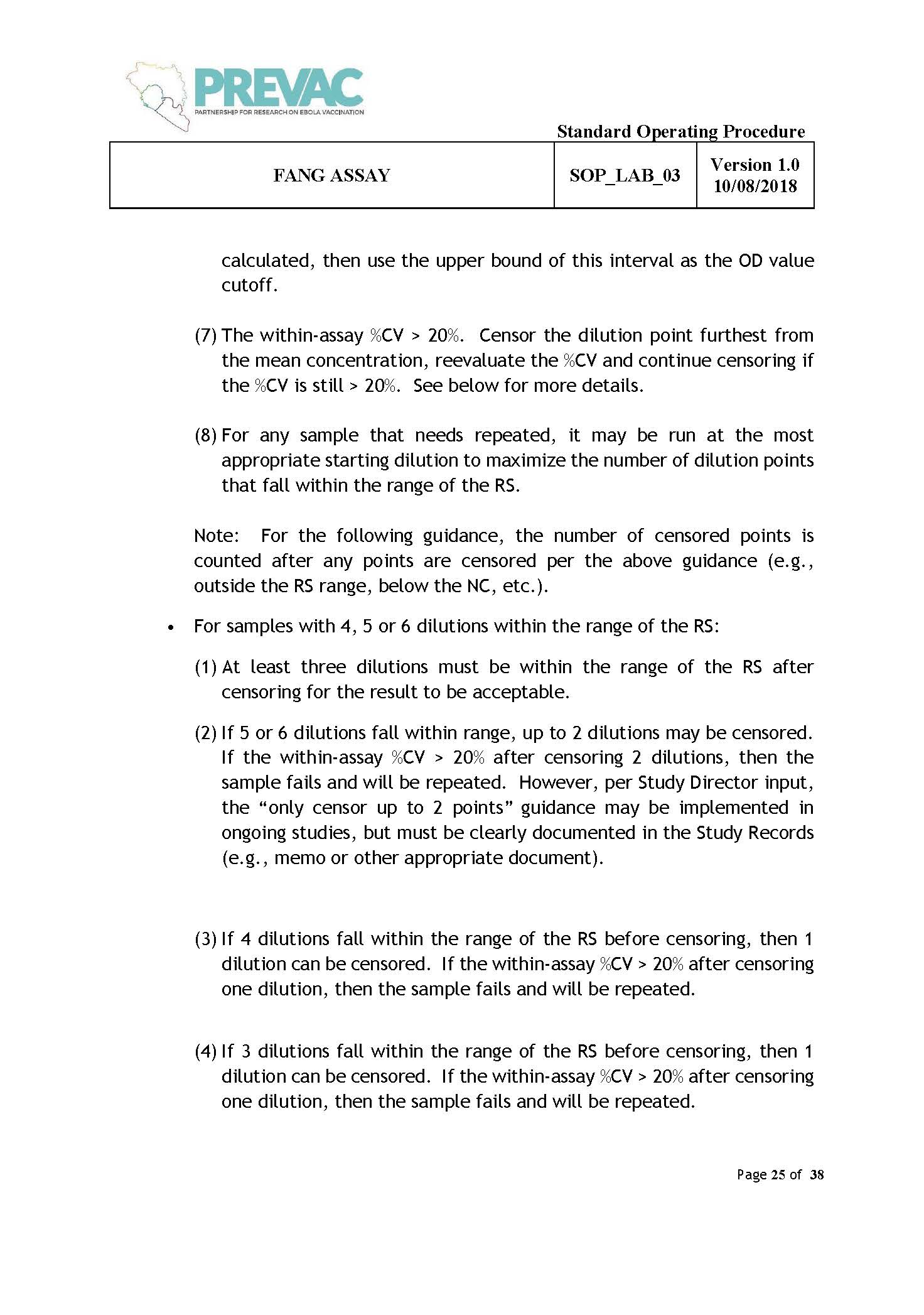

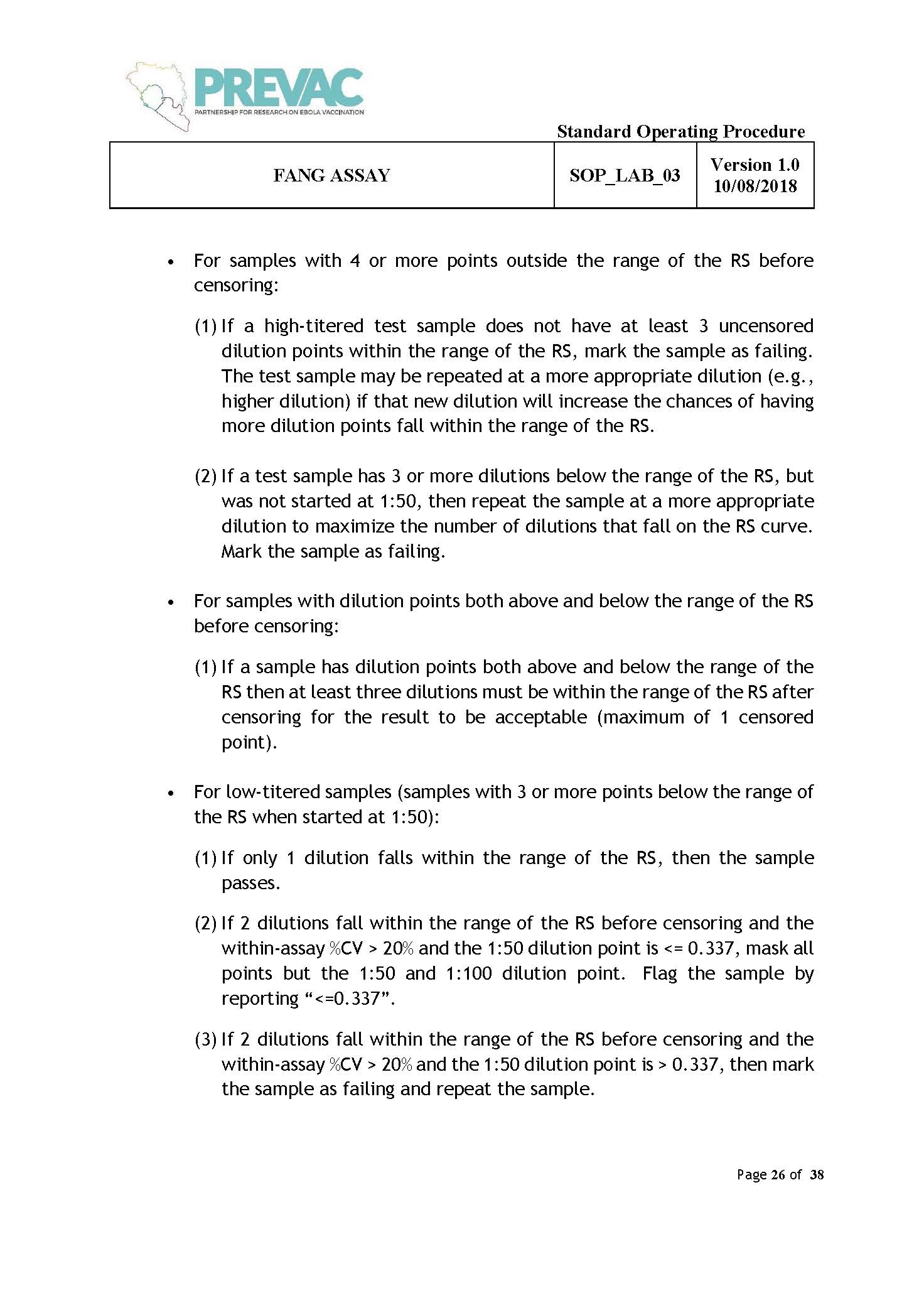

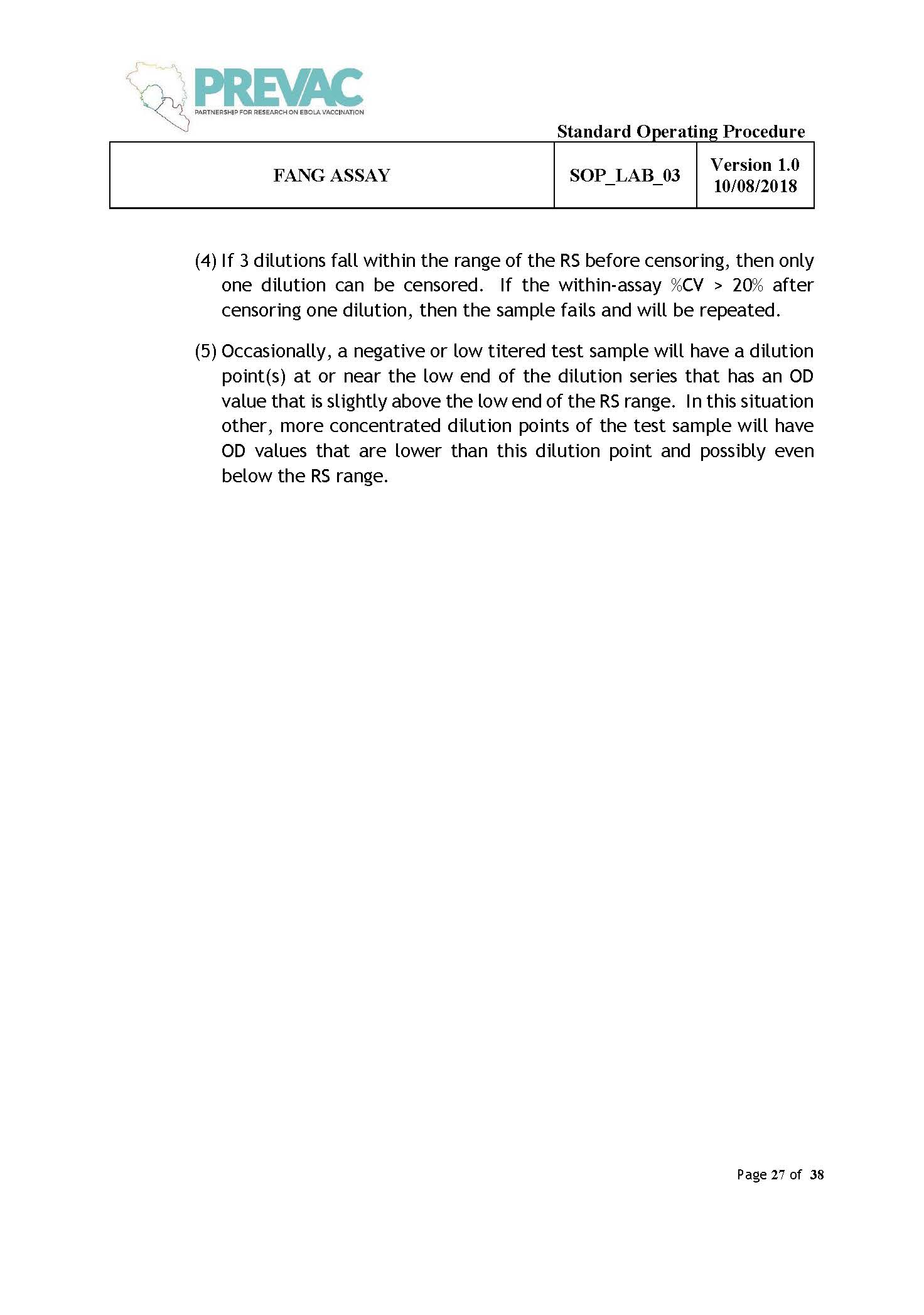

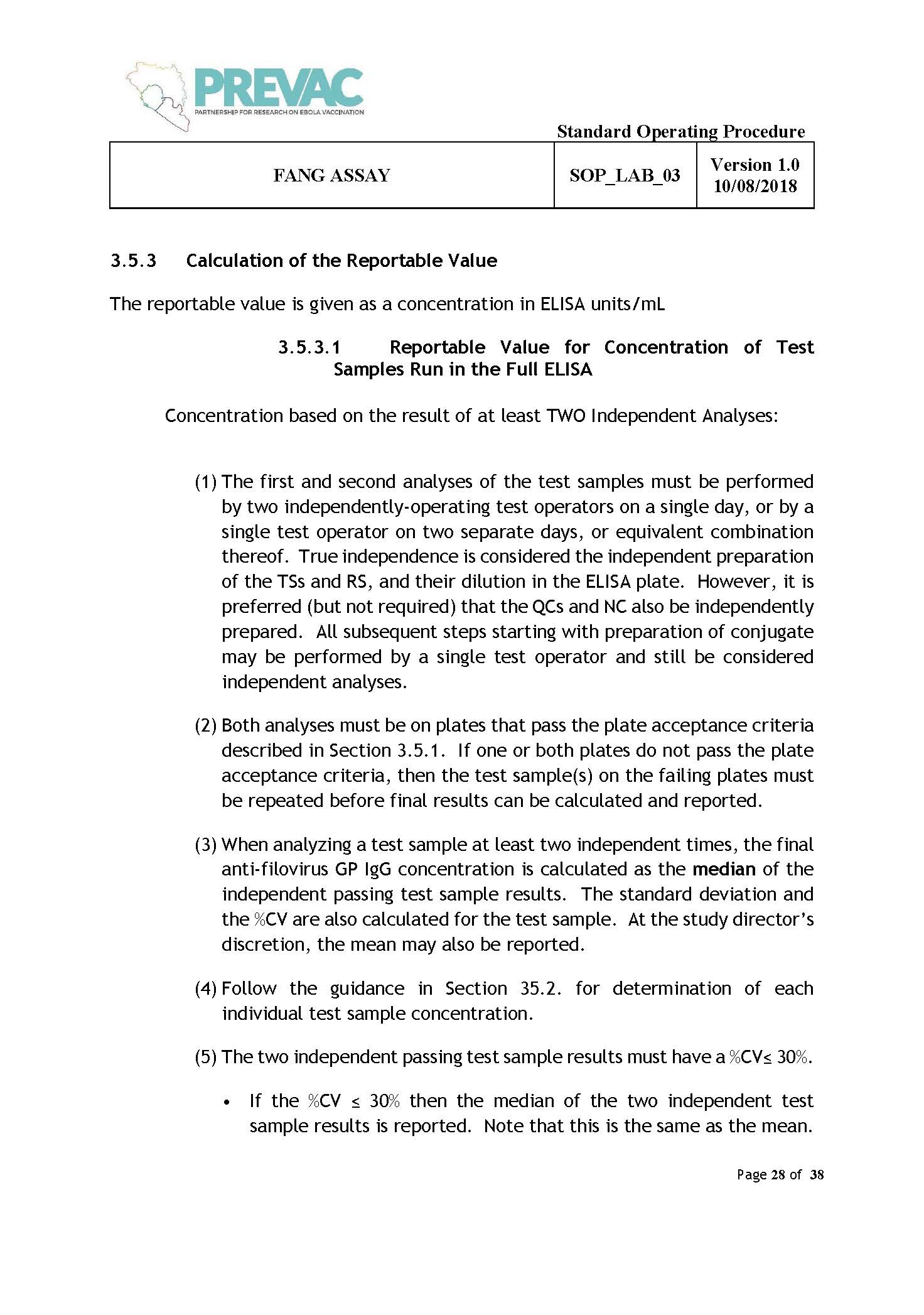

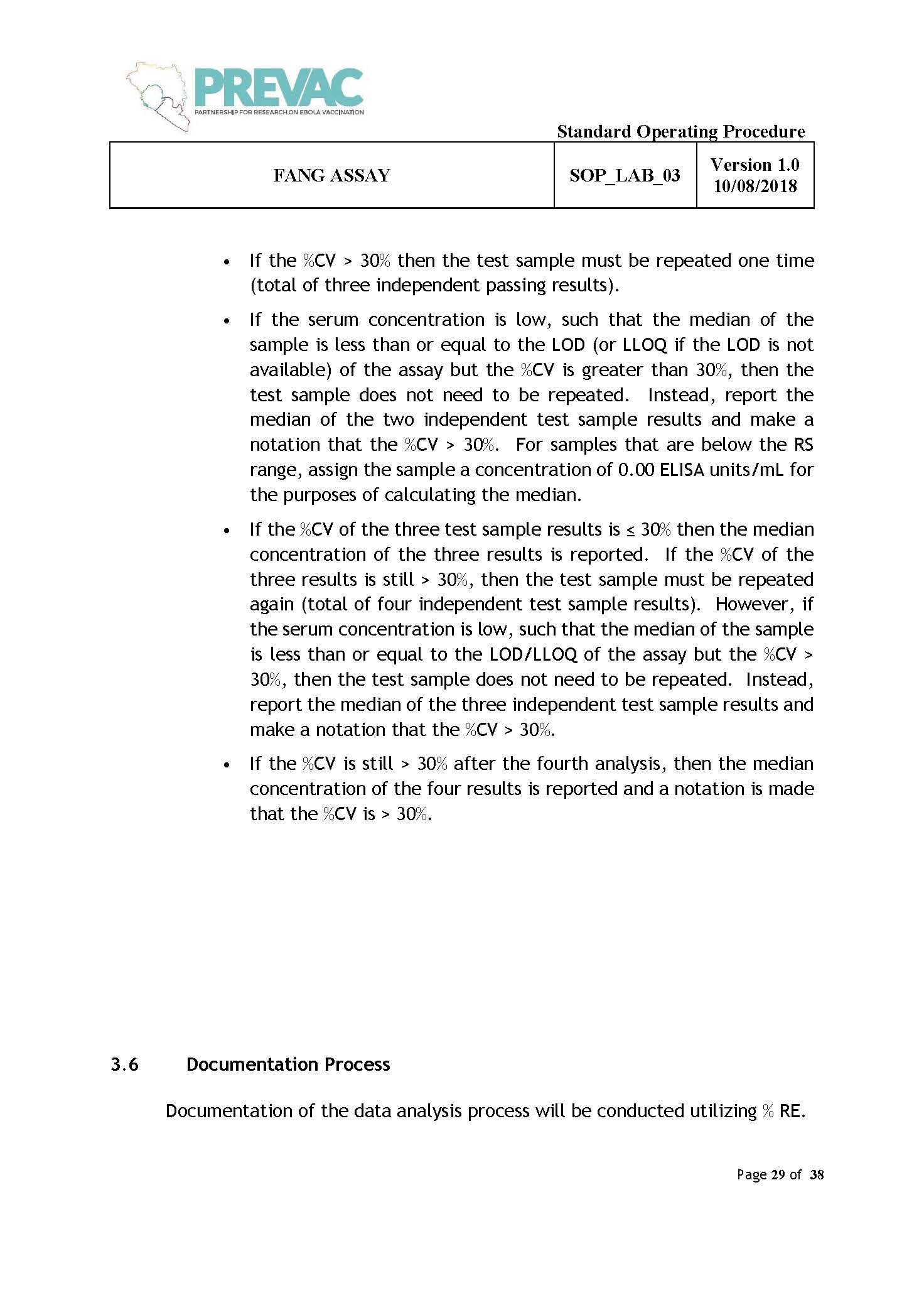

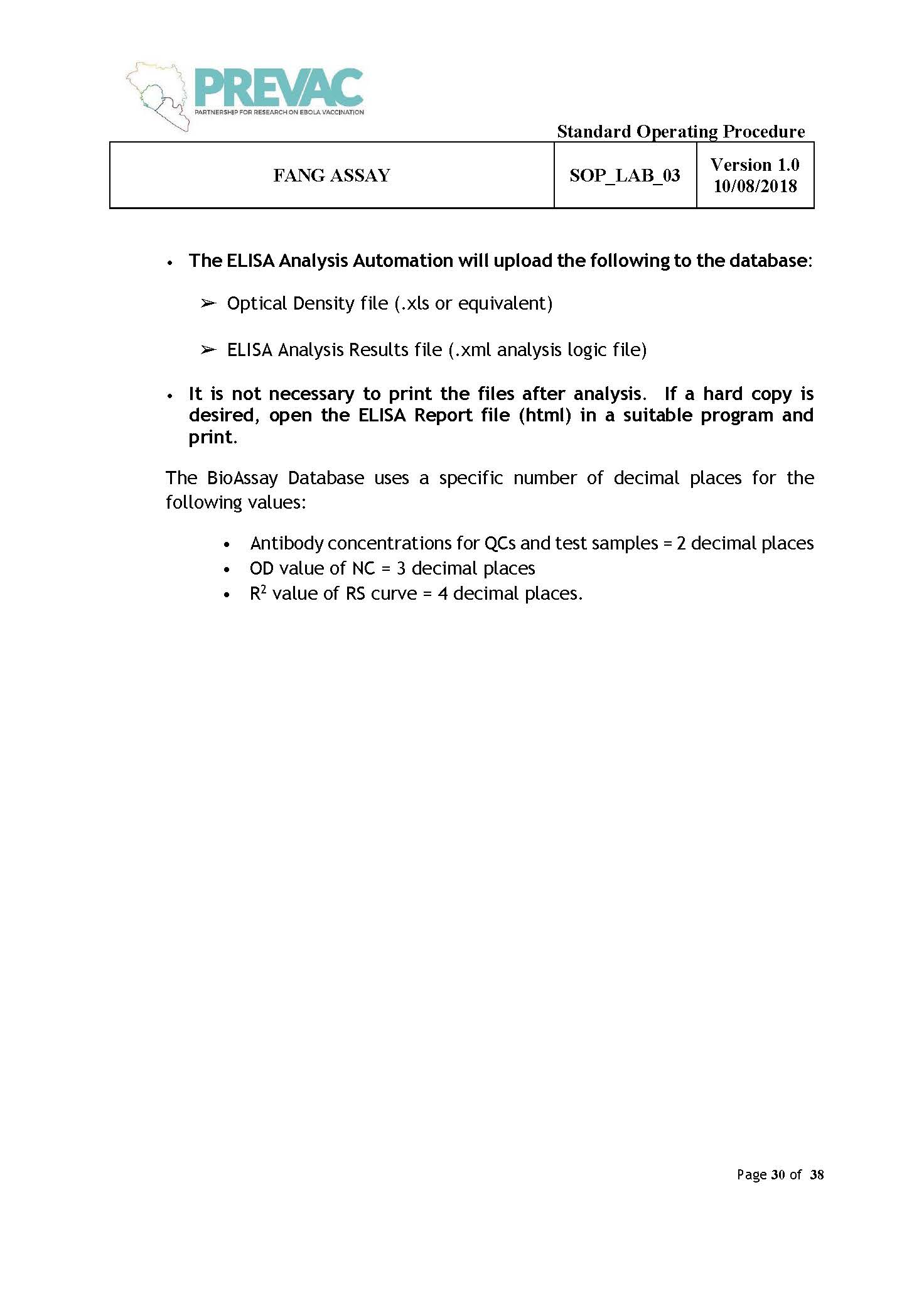

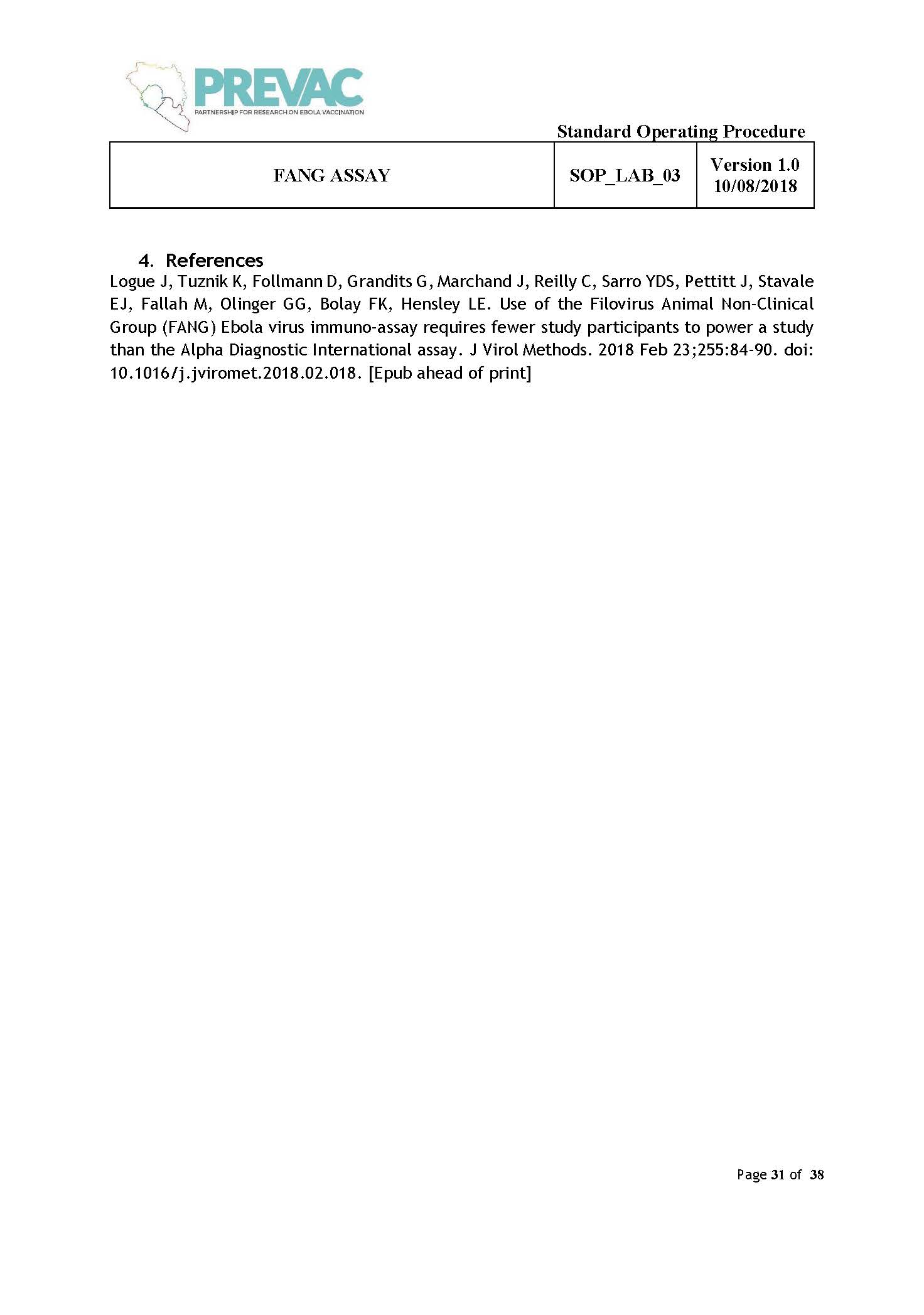

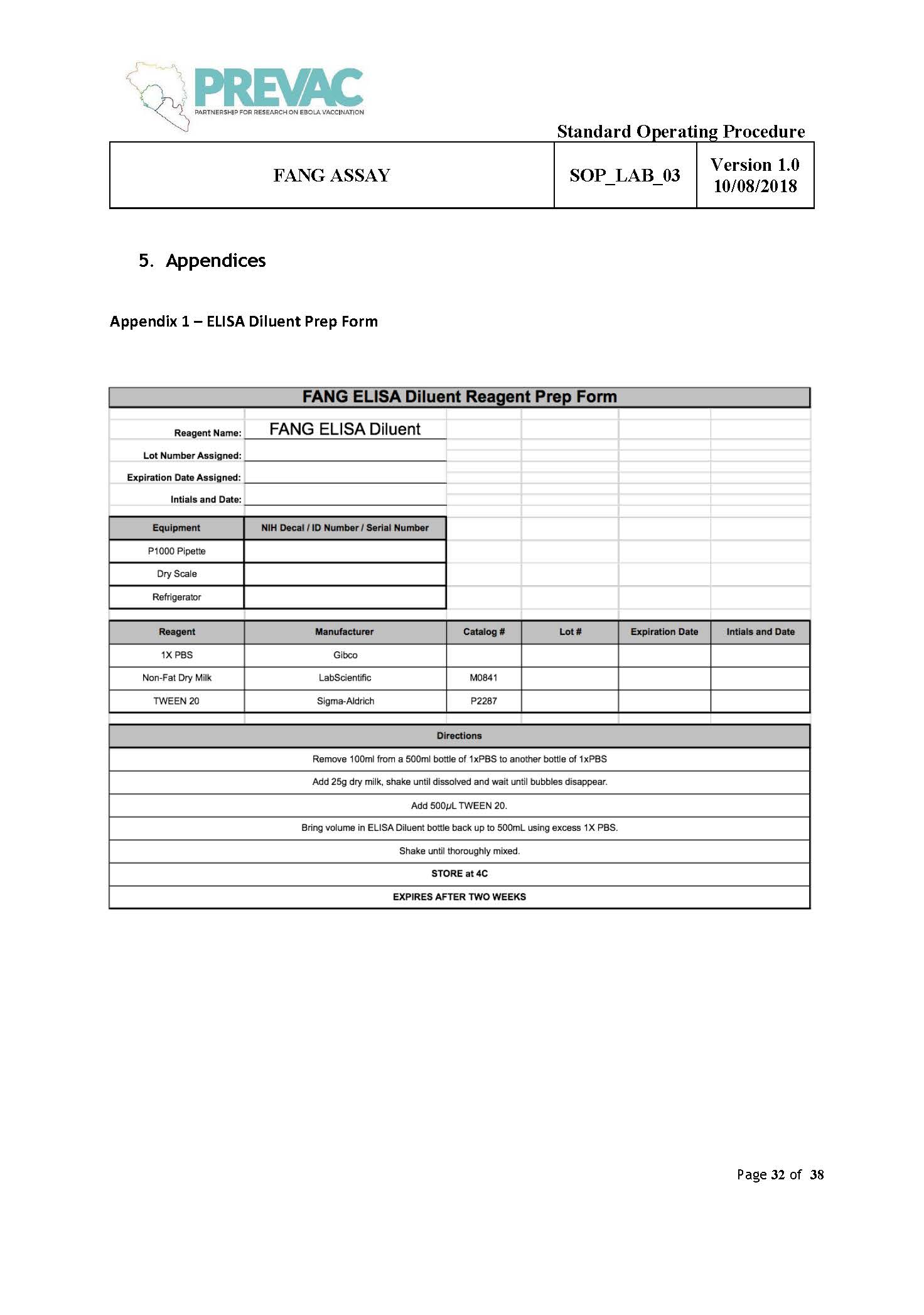

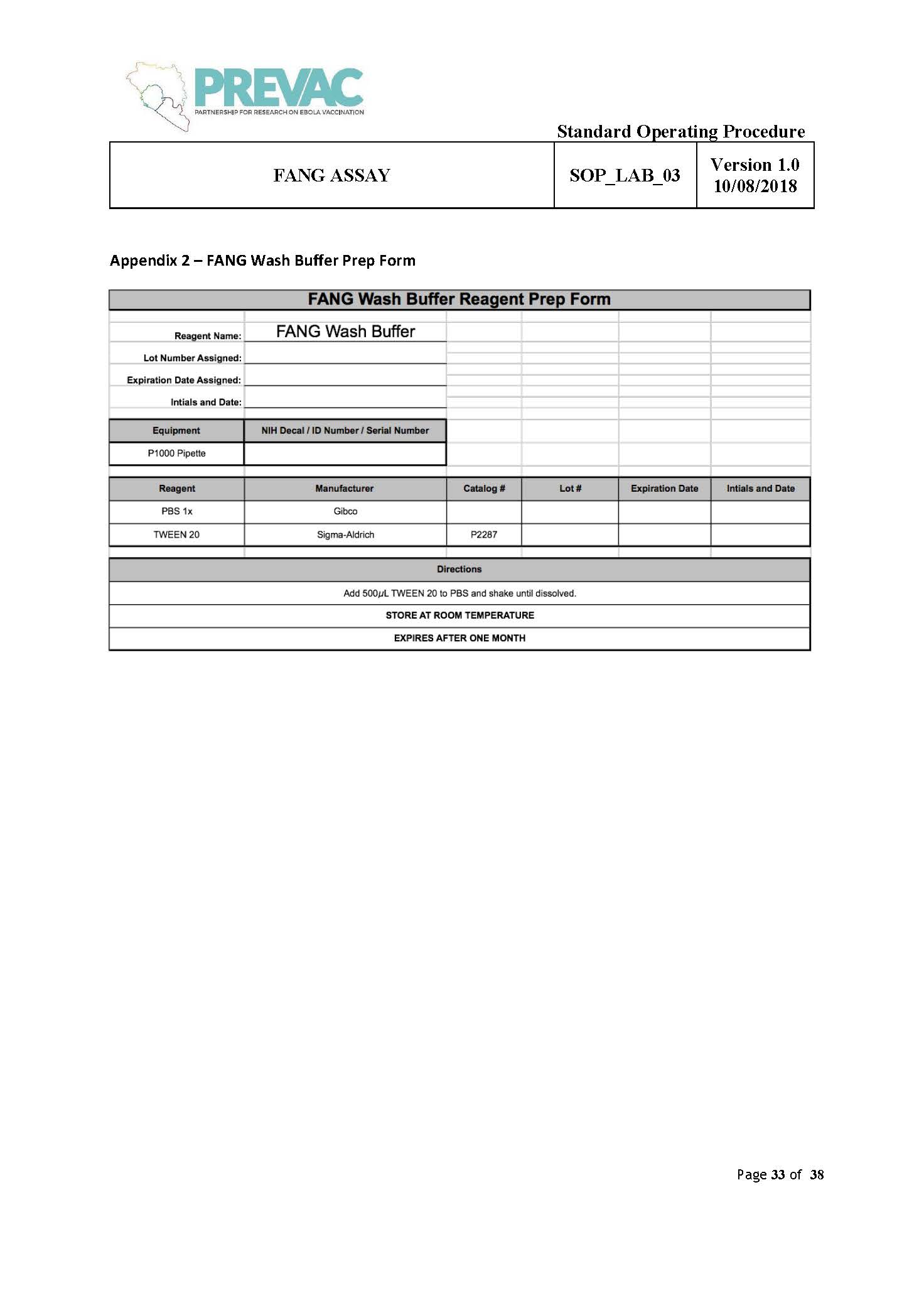

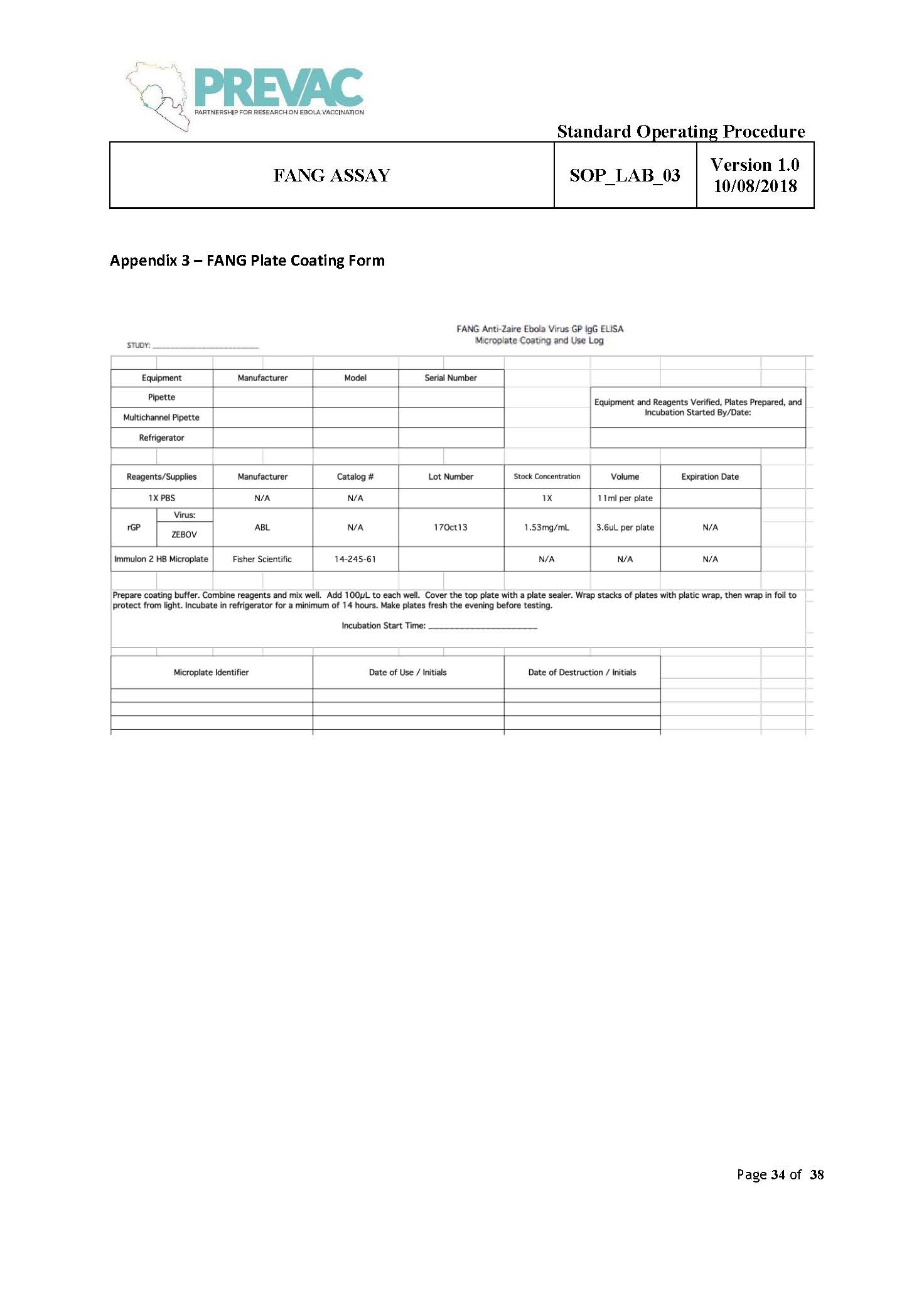

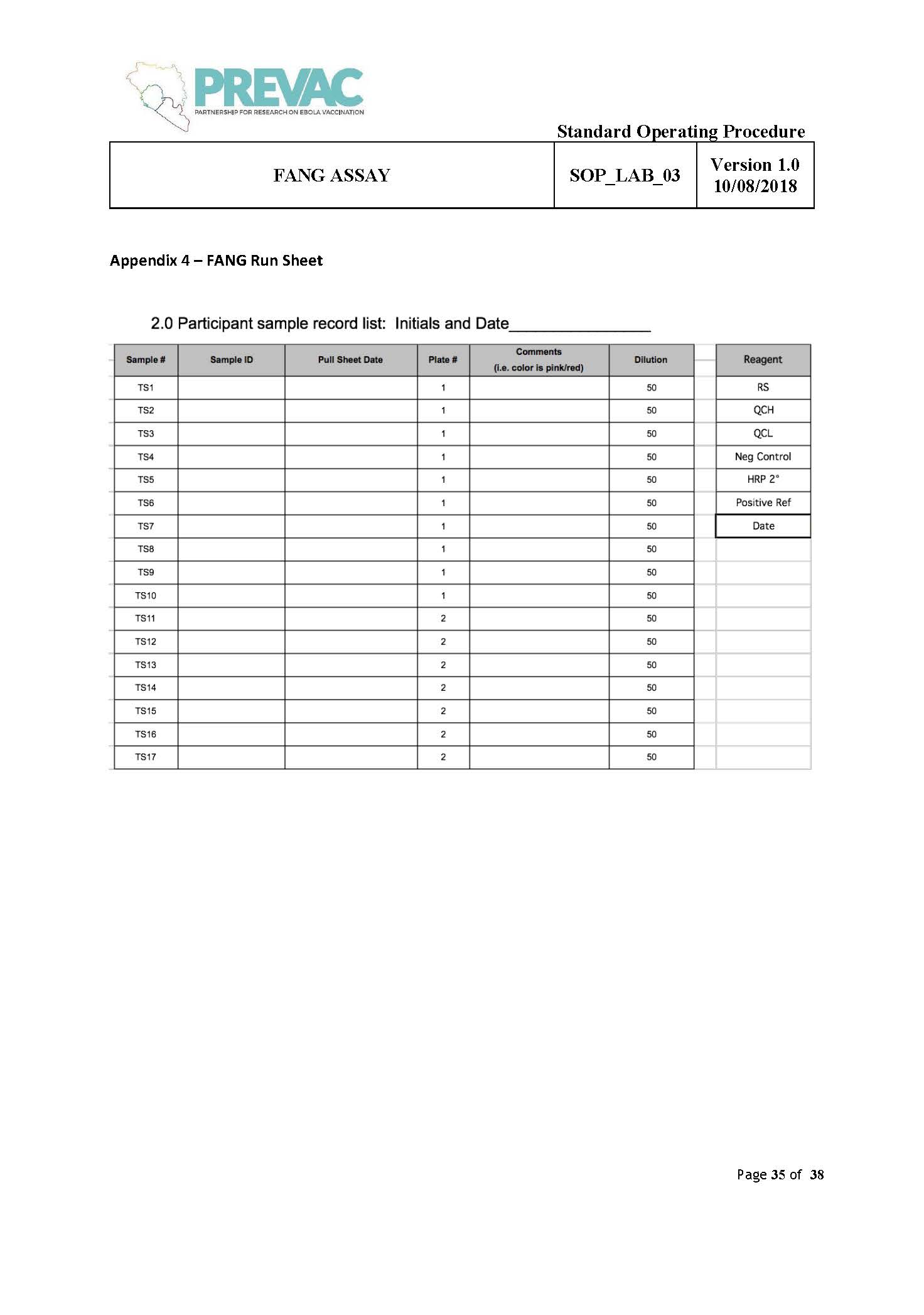

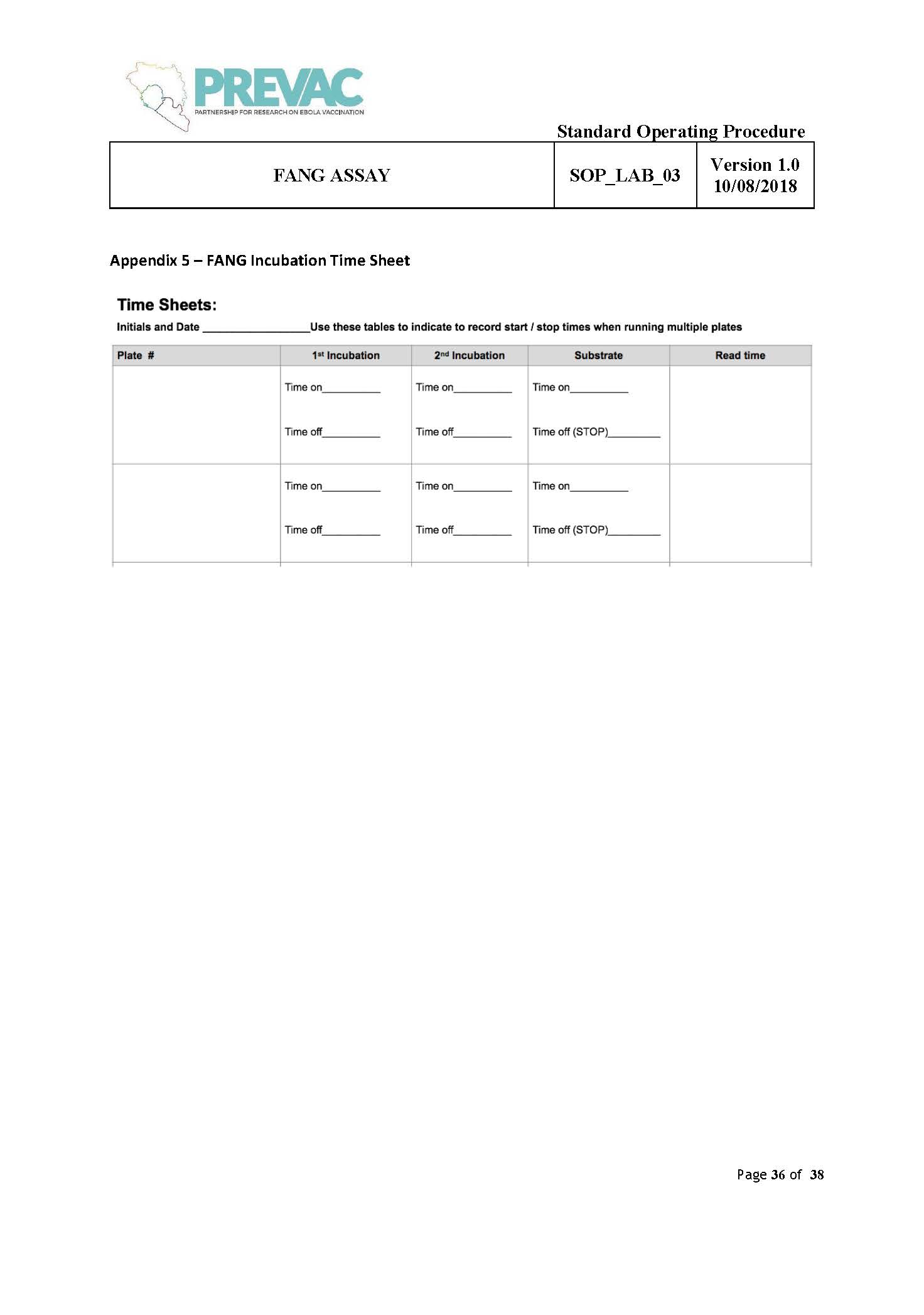

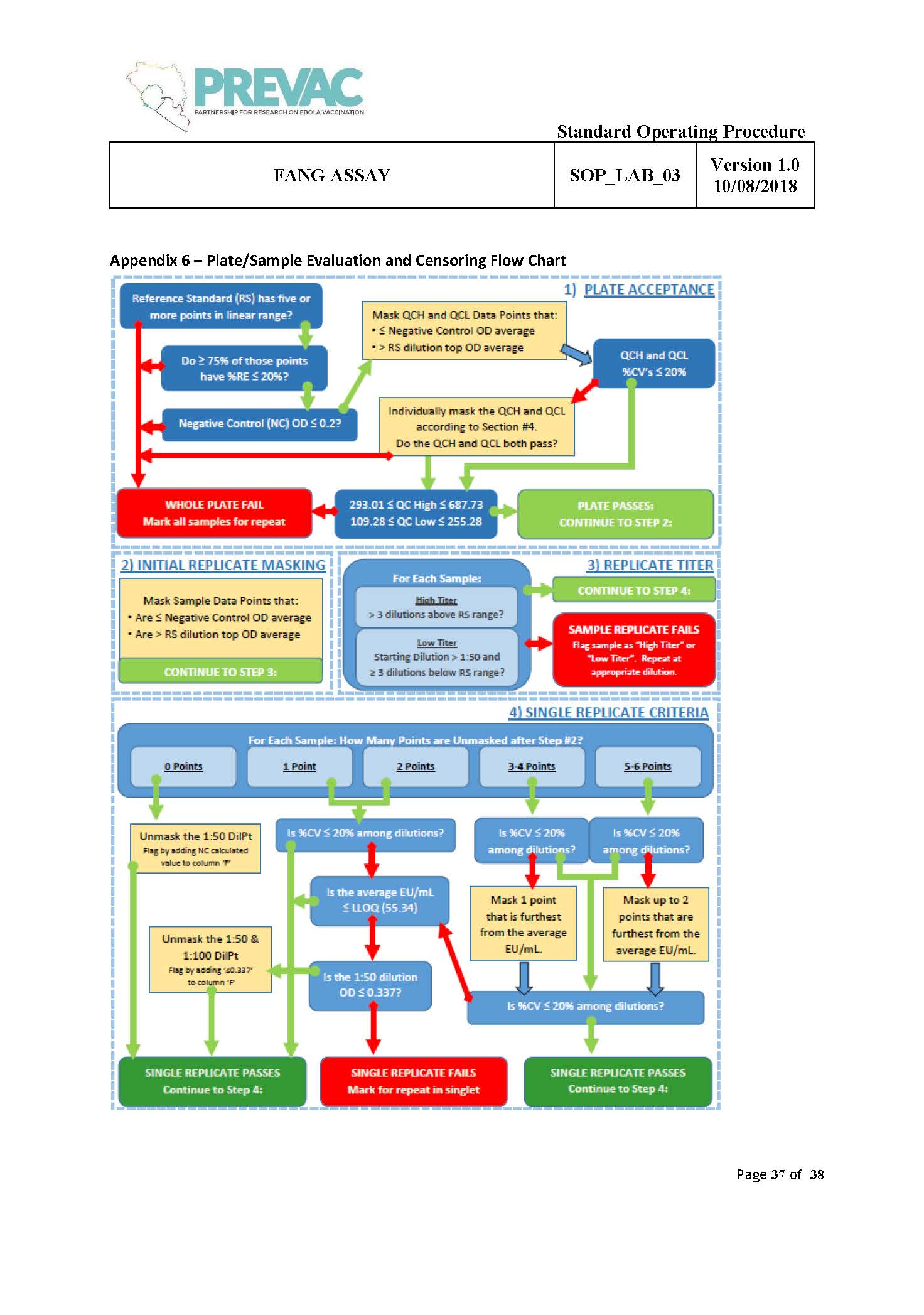

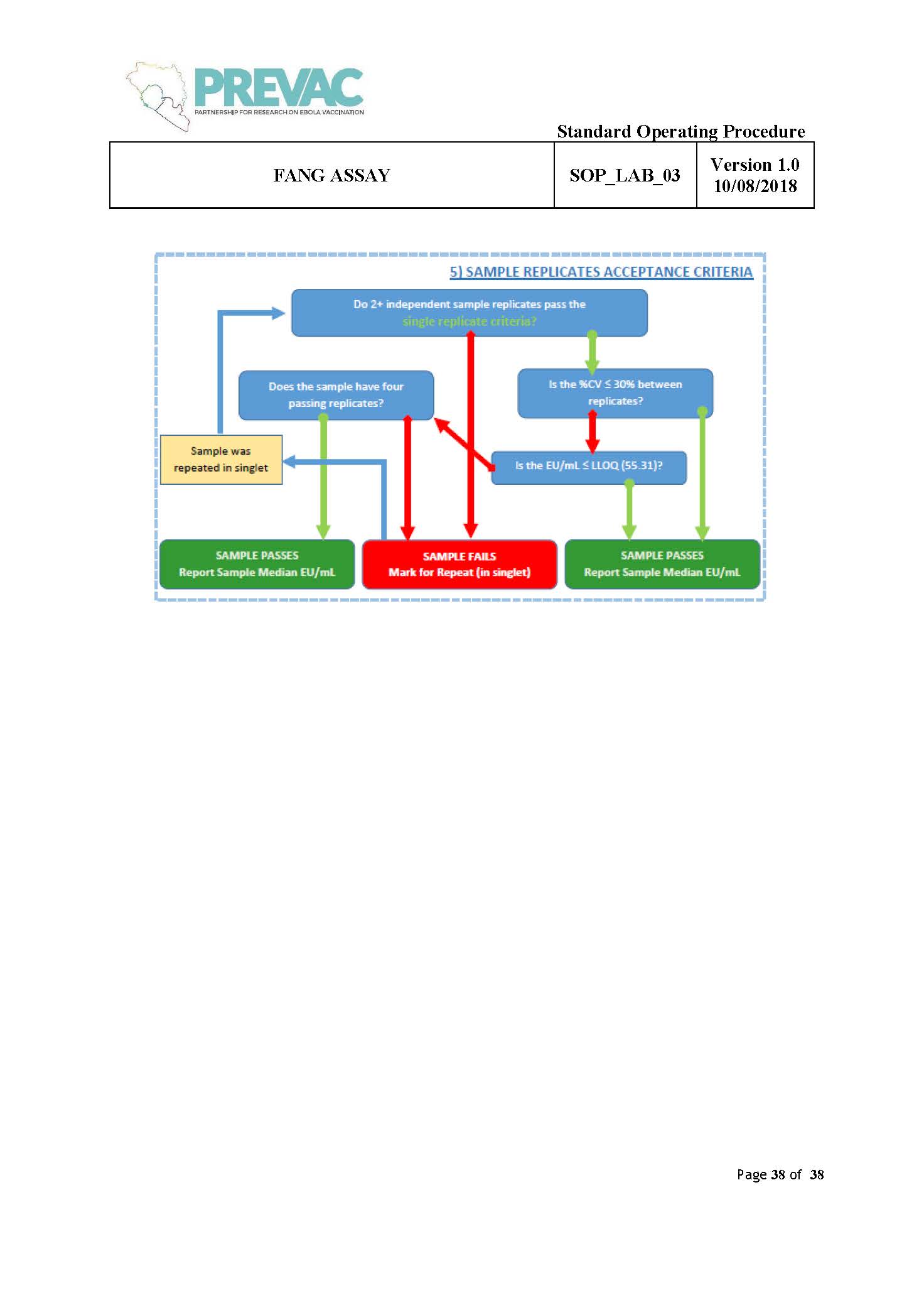


## **PREVAC Study Team**

Jamila Aboulhab^2^ M.D., Michelle Aguirre-MacKenzie^12^ B.S., Pauline Akoo^3^ M.B., Ch.B., Esther Akpa^2^ M.S.N., M.P.H., R.N., Robert Akpata^1^ M.D., Sara Albert^17^ B.A., M.P.H, Boni Maxime Ale^4^ M.D., M.Sc., M.P.H., Serry Alimamy-Bangura^11^ M.B., Ch.B., Pierre Andong^1^ M.St., Benetta C. Andrews^10^ M.D., Stephane Anoma^6^ M.D., Negin Atri^2^ M.P.H., C.P.H., Augustin Augier^6^ M.Com., Ken Awuondo^3^ M.Sc., Ahidjo Ayouba^30^ Ph.D., Moses Badio^10^ M.Sc., Aminata Bagayoko^1^ M.D., Abby Balde^17^ M.P.H., Joséphine Balssa^1,8^ Pharm.D., Lamin Molecule Bangura^11^ B.Sc., Kesha Barrington^17^ M.P.A., Eric Barte de Saint Fare^6^ B.A., Beth Baseler^17^ M.S., Ali Bauder^12^ B.A., P.M.P., Claire Bauduin^4^ M.Sc., Luke Bawo^10^ M.Sc., Abdoul Habib Beavogui^9^ M.D., Ph.D., Michael Belson^2^ B.S., Safaa Ben-Farhat^4^ M.Eng., Marion Bererd^6^ B.S., Nicolas Bernaud^1,18^ M.Sc., Teedoh Beyslow^10^ Pharm.D., Neirade Biai^30^ M.Sc., Jeanne Billioux^2^ M.D., Shere Billouin-Frazier^17^ M.Sc., Blandine Binachon^4^ M.D., M.P.H., Julie Blie^10^ M.Sc., Viki Bockstal^13^ Ph.D., Patricia Boison^17^ M.S., Fatorma Bolay^10^ Ph.D., Aliou Boly^6^ M.M., Rachael Elizabeth Bonawitz^12^ M.D., M.S., Anne-Gaëlle Borg^6^ M.C.M., Samuel Bosompem^2^ Pharm.D. M.Sc., Courtney Bozman^28^ M.Sc., Tyler Brady^2^ M.P.H., Sarah Browne^10^ R.N., B.S.N., Ryan Bullis^12^ Ph.D.,Barbara Cagniard^1^ Ph.D., Kelly Cahill^2^ R.N, M.Sc., C.C.R.C., R.A.C., Yingyun Cai^28^ Ph.D., Aissata Abdoulaye Camara^6^ M.Sc., Aboubacar Keira Camara^1^ M.D., Alseny Modet Camara^6^ M.D., Antoine Campagne^1^ Ph.D., Cécilia Campion^4^ M.Sc., Alexandre Cantan^4,29^ B.Sc., Jennifer Cash^17^ B.S., Siew Pin Chai^13^ B.Bio-Med.Sc., Francois Chambelin^1^ M.Hist., Michael Chea^10^ B.Sc., Geneviève Chêne^4^ M.D., Ph.D., Edouard Choi^3^ Ph.D., Michelle Chouinard^6^ M.S.W., Florence Chung^1^ Ph.D., Lucy Chung^2^ Pharm.D., Séverine Ciancia^1^ M.J., Papa Ndiaga Cisse^14^ Ph.D., Elfrida Cline-Cole^17^ M.A., Céline Colin^4^ M.Sc., Beth-Ann Coller^12^ , Ph.D., Djélikan Siaka Conde^1^ M.D^.^, Katherine Cone^2^ M.S.W., LCSW-C, C-SWHC, Laurie Connor^12^ M.S., Nicholas Connor^3^ M.Sc., Joseph Boye Cooper^10^ M.Sc., Sandrine Couffin-Cardiergues^1^ Ph.D., Fatoumata Coulibaly^1^ B.S., Mariam Coulibaly^20^ Pharm.D., M.Sc., Page Crew^2^ Pharm.D., M.P.H, B.C.P.S., Sandrine Dabakuyo-Yonli^4^ Pharm.D., Ph.D., Djeneba Dabitao^20^ Pharm.D., Ph.D., Thierry Damerval^1^ Ph.D., Bionca Davis^5^ M.P.H., Gibrilla Fadlu Deen^11^ M.D., M.Sc., Eline Dekeyster^13^ Ph.D., Jean-François Delfraissy^1^ M.D., Ph.D., Christelle Delmas^1^ M.Sc., Mahamadou Diakite^20^ Pharm.D., D.Phil, Alpha Diallo^1,8^ M.D., M.P.H., Fatoumata Abdoulaye Diallo^6^, M.D., Mamadou Saliou Diallo^6^ M.D., M.P.H., Ayouba Diarra^20^ M.Sc., Samba Diarra^20^ M.Sc., Ph.D., Oualy Diawara^19^ B.S., Ilo Dicko ^20^ M.D, M.P.H., Bonnie Dighero-Kemp^28^ B.Sc., Samba Diop^21^ M.Sc., Ph.D., Waly Diouf^14^ Ph.D., Saurabh Dixit^2^ Ph.D., Barry Djenabou^6^ M.Sc., Laurie Doepel^2^ B.A., Eric D'Ortenzio^1,7,8^ M.D., M.P.H., Seydou Doumbia^20^ M.D., Ph.D., Moussa Moise Doumbia^19^ M.D., Macaya Douoguih^13^ M.D., M.P.H., Nelson Dozier^28^ M.Sc., Natasha Dubois Cauwelaert^1,8^ Ph.D., Alain DuChêne^5^ B.S., Michael Duvenhage^17^ N.DIP.IT., Risa Eckes^2^ R.N., Elizabeth Elliott^2^ M.Sc., Luisa Enria^3^ Ph.D., Hélène Espérou^1^ M.D., Cécile Etienne^1^ M.Sc., Allison Eyler^17^ H.S.D., Lawrence Fakoli^10^ M.Sc., Mosoka Fallah^10^ Ph.D., Marie-Alix Fauvel^1^ M.Sc., Sylvain Faye^14^ Ph.D., John Fayiah^10^ M.Sc., Suzanne Fleck^3^ Ph.D., Vemy Fofana^6^ B.Comp, Karine Fouth Tchos^2^ M.D., M.P.H., Kokulo Franklin^10^ M.phil., M.Sc., Daniela Fusco^1^ Ph.D., Auguste Gaddah^13^ Ph.D., Marylène Gaignet^1^ M.Sc., Katherine Gallagher^3^ Ph.D., Julie Gardner^12^, B.S., Harrison Gichini^28^ M.Sc., Julia Garcia Gozalbes^1^ M.D., Greg Grandits^5^ M.S, Maima Gray^10^ B.Pharm., Brian Greenwood^3^ M.D., Nico Grobler^13^ Ph.D., Robin Gross^28^ M.Sc., Louis Grue^17^ R.N., B.S., B.S.N., Birgit Grund^27^ Ph.D., Oumar Guindo^20^ M.Sc., Pharm.D., Swati Gupta^12^ Dr.P.H., M.P.H., Fadima Haidara^19^ M.D., Benjamin Hamzé^1^ Pharm.D., Emma Hancox^3^ M.Sc., Jean-Christophe Hébert^1^ M.S.L., Jenny Hendriks^13^ Ph.D., Patricia Hensley^3^ M.Ph., Lisa E. Hensley^28^ Ph.D., M.S.P.H., Betsey Herpin^2^, M.S.N, Elisabeth Higgs^2^ M.D., D.T.M.H., M.I.A., Trudi Hilton^3^ B.Pharm., M.Sc., Mickael Hneino^1^, Ph.D., Tracey-Ann Hoeltermann^28^, B.Sc., M.P.H., , Horace Preston Holley^17^ M.D., Marie Hoover^16^ Ph.D., Natasha Howard^3^ Ph.D., Melissa Hughes^12^ B.A., M.B.A., C.P.M., P.M.P., Skip Irvine^12^ B.S., David Ishola^3^ M.D., Ph.D., Yvonne Jato^2^ M.P.H., Madison Joe^10^ M.Sc., Melvin Johnson^10^ M.Sc., Aboubacar Sidiki Kaba^6^ M.D., Jonathan Kagan^2^ Ph.D., Kade Kallon^17^, M.Sc., Michael Kamara^3^ MB.ChB, M.Sc., Myriam Kante^4^ B.S., Judith Katoudi^6^ M.D., M.P.H., Cheick Mohamed Keita^6^ M.D., Sakoba Keita^15^ M.D., Seykou Keita^22^ M.D., Stephen B. Kennedy^10^ M.D., M.Sc., Babajide Keshinro^13^ M.B.B.S., F.W.A.C.P., Hassan Kiawu^10^ M.Sc., Mark Kieh^10^ M.D., M.S.M.H.C., Brent Killinger^12^ B.A., Moumouni Kinda^6^ M.D., M.B.A., Matthew Kirchoff^2^ Pharm.D., M.Sc., M.B.A., Gregory Kocher^28^ M.Sc., Mamoudou Kodio^19^ Pharm.D., Brian Kohn^3^ B.Sc., Lamine Koivogui^23^ Pharm.D., Ph.D., Richard Kojan^6^ M.D., Cece Francis Kolié^6^ Pharm.D., Jacques Seraphin Kolié^6^ M.D., David Kollie^10^ B.Sc., Stacy Kopka^17^ M.S., Bockarie Koroma^11^ B.Pharm., Dickens Kowuor^3^ B.Sc., M.Sc., Ph.D., Catherine Kpayieli-Freeman^10^ M.Sc., Liane Kwast^13^ M.Sc., Christine Lacabaratz^1,18^ Ph.D., Boris Lacarra^1^ M.D., Laurie Lambert^17^ B.S., Courtney Lambeth^12^ B.S., Solange Lancrey-javal^1,8^ Pharm.D., H. Clifford Lane^2^ M.D., Shadrach Langba^10^ B.Sc., Bolarinde Lawal^3^ M.Sc., Andrew Wen-Tseng Lee^12^ M.D., Shona Lee^3^ Ph.D., Shelley Lees^3^ Ph.D., Annabelle Lefevre^1^ M.D., Bailah Leigh^11^ M.D., M.Sc., Frederic Lemarcis^1^ Ph.D., Yves Lévy^1,18^ M.D., Ph.D., Claire Levy-Marchal^1^ M.D., Maarten Leyssen^13^ M.D., Ph.D., Edouard Lhomme^26^ M.D., Ph.D., Janie Liang^28^ M.Sc., Mameni Linga^10^ M.Sc., Ken Liu^12^ Ph.D., Brett Lowe^3^ M.Phil., Julia Lysander^10^ M.Sc., Ibrah Mahamadou^6^ Pharm.D., Irina Maljkovic-Berry^28^, Ph.D.*,* Marvington Mambiah^10^ A.Sc., Daniela Manno^3^ M.D., Ph.D., Jonathan Marchand^2,17^ M.S., Lindsay Marron^28^ M.Sc., Moses B.F. Massaquoi^10^ M.D., M.Sc., Laure Masson^1^ M.I.B.L., Charly Matard^4^ B.S., Steven Mazur^28^ B.S., John McCullough^16^ B.S., Katherine McFadyen^13^ M.P.H., Chelsea McLean^13^ Ph.D., Noémie Mercier^1^ Pharm.D., Pauline Michavila^6^ B.Bus., Tracey Miller^17^ R.N., B.S.N., Niouma Pascal Millimouno ^6^ M.D., Alejandra Miranda^17^ M.S., Soumaya Mohamed^6^ B.J., Tom Mooney^3^ B.A., Dally Muamba^6^ M.D., James Mulbah^11^ B.Pharm., Rita Lukoo Ndamenyaa^6^ M.D., M.Sc., James Neaton^5^ Ph.D., Désiré Neboua^1^ M.D., Micki Nelson^12^ B.S.N., M.S., Kevin Newell^17^ M.P.H., M.Ed., Vinh-kim Nguyen^24^ M.D., Yusupha Njie^3^ B.Sc., Wissedi Njoh^17^ M.S.N., Anna Novotney-Barry^13^ M.SC., Matthew Onorato^12^ B.S., Uma Onwuchekwa^22^ B.Sc., Susan Orsega^2^ M.S.N., FNP-BC, Inmaculada Ortega-Perez^1,8^ Ph.D., M.P.H., Cynthia Osborne^17^ B.S., Tuda Otieno^3^ M.SC., Davy Oulaï^4^ M.D., Sushma Patel^12^ M.S., P.M.P., Danielle Peart^2^ B.S., Martine Peeters^30^ Ph.D., James Pettitt^28^ M.Sc., Nathan Peiffer-Smadja^1^ M.D., Ph.D., Robert Phillips^3^ M.Sc., Jerome Pierson^2^ Ph.D., Peter Piot^3^ M.D., Ph.D., Micheal Piziali^2^ J.D., M.Sc., Stéphany Pong^1,8^ Pharm.D., Elena Postnikova^2^ Ph.D., Calvin Proffitt^17^ M.A., Alexandre Quach^1^ M.D., Sinead Quigley^1^ M.S.Sc., Nadeeka Randunu^2,17^ B.Sc., M.B.A., Laura Richert^26^ M.D., Ph.D., Priscille Rivière^1^ M.Sc., Cynthia Robinson^13^ M.D., Céline Roy^4,29^, Ph.D., Amy Falk Russell^12^ M.S., Philip Sahr^10^ M.D., Katy Saliba^2^, M.Sc., Ph.D., Mohamed Samai^11^ M.B.B.S., Ph.D., Sibiry Samake^20^ Pharm.D., M.Sc., Jen Sandrus^17^ A.A., Ibrahim Sanogo^20^ Ms.P., M.D., Yeya Sadio Sarro^20^ Pharm.D., Ph.D., Serge Sawadogo^6^ M.D., M.Sc., Sani Sayadi^6^ M.D., M.P.H., Maxime Schvartz^1^ M.D., Christine Schwimmer^4^ Ph.D., Fatou Secka^3^ B.Sc., M.Sc., MB.ChB, Heema Sharma^28^M.Sc., Denise Shelley^17^ M.S., Bode Shobayo^10^ M.Sc., Sophia Siddiqui^2^ M.D., M.P.H., Jakub Simon^12^ M.D., Shelly Simpson^17^ M.S., Billy Muyisa Sivahera^6^ M.D., Karen Slater^3^, Mary Smolskis^2^ B.S.N., M.A., Elizabeth Smout^3^ M.D., M.Sc., Emily Snowden^3^ M.A., Anne-Aygline Soutthiphong^4,29^ M.Sc., Amadou Sow^6^ M.Sc., Samba O. Sow^22^ M.D., M.Sc., Ydrissa Sow^2^ M.D., M.P.H., Michael Stirratt^25^ Ph.D., Jeroen Stoop^13^ Ph.D., Guna Subramaniam^13^ M.Sc., Léa Surugue^1^ M.J., Nathalie Swales^13^ M.Sc., Sienneh Tamba^10^ R.N., B.S.N., Chan Tang^13^ B.Sc., Cheick Tangara^20^ M.Sc., Milagritos D. Tapia^22^ M.D., Julius Teahton^10^ M.Sc., Jemee Tegli^10^ M.Sc., Monique Termote^4^ M.Sc., Guillaume Thaurignac^30^ M.SC., Rodolphe Thiebaut^4^ M.D., Ph.D., Greg Thompson^5^ B.S., John Tierney^2^ B.S.N., M.P.M., Daniel Tindanbil^3^ M.Sc., Abdoulaye Touré^23^ Pharm.D., M.P.H., Ph.D., Elvis Towalid^10^ B.Pharm, Stacey Traina^12^ B.S., Awa Traore^19^ Pharm.D., Tijili Tyee^10^ Pharm.D., David Vallée^1^ Pharm.D., Renaud Vatrinet^1^ Ph.D., Corine Vincent^4^ M.Sc., Susan Vogel^2^ R.N, B.S.N., Cedrick Wallet^4^ M.Sc., Travis Warren^2^ Ph.D., Deborah Watson-Jones^3^ M.D., Ph.D., Wade Weaver^28^ M.Sc., Deborah Wentworth^5^ M.P.H., Cecelia Wesseh^10^ B.Sc., Hilary Whitworth^3^ Ph.D., Jimmy Whitworth^3^ Ph.D., Aurelie Wiedemann^1,18^ Ph.D., Wouter Willems^13^ Ph.D, Barthalomew Wilson^10^ M.Sc., Jayanthi Wolf^12^ Ph.D., Alie Wurie^11^ M.D., M.Sc., Delphine Yamadjako^17^ M.S., Marcel Yaradouno^6^ M.Sc., Quiawiah Yarmie^10^ M.Sc., Yazdan Yazdanpanah^1,7,8^ M.D., Ph.D., Shuiqing Yu^28^ B.S., Zara Zeggani^6^ M.Sc., Huanying Zhou^28^ B.S.

## Affiliations

^1^ French Institute for Health and Medical Research (Inserm), 75013 Paris, France

^2^ National Institute of Allergy and Infectious Diseases, Bethesda, MD, USA or under contract/subcontract to NIAID

^3^ London School of Hygiene & Tropical Medicine, London, UK

^4^ Univ. Bordeaux, INSERM, Institut Bergonié, CHU de Bordeaux, CIC-EC 1401, Euclid/F-CRIN clinical trials platform, F-33000 Bordeaux, France

^5^ School of Public Health, University of Minnesota, Minneapolis, MN, USA

^6^ The Alliance for International Medical Action, Alima, B.P.15530 Dakar, Sénégal

^7^ AP-HP, Hôpital Bichat-Claude Bernard, Service de Maladies Infectieuses et Tropicales, Paris F-75018, France

^8^ ANRS Emerging Infectious Diseases, Paris, France

^9^ Centre National de Formation et de Recherche en Santé Rurale de Maferinyah, Maferinyah, Guinea

^10^ Partnership for Research on Ebola Virus in Liberia (PREVAIL), Monrovia, Liberia

^11^ College of Medicine and Allied Health Sciences (COMAHS), University of Sierra Leone, Freetown, Sierra Leone

^12^ Merck Sharp & Dohme Corp, Inc., Kenilworth, NJ, USA

^13^ Janssen Vaccines and Prevention BV Leiden, The Netherlands

^14^ Département de Sociologie, FLSH, Université Cheikh Anta DIOP, Dakar, Sénégal

^15^ Agence Nationale de Sécurité Sanitaire, Conakry, Guinea

^16^ Advanced BioMedical Laboratories, L.L.C., 1605 Industrial Hwy, Cinnaminson, NJ, USA

^17^ Leidos Biomedical Research, Inc. Frederick, MD 21704, USA

^18^ Vaccine Research Institute, Univ. Paris Est Créteil, Henri Mondor Hospital, Créteil, France

^19^ Centre pour le Développement des Vaccins, Ministère de la Santé, Bamako, Mali

^20^ University Clinical Research Center (UCRC), University of Sciences, Techniques and Technologies of Bamako (USTTB), Bamako, Mali

^21^ Liberia Institute for Biomedical Research Ethics Committee/National, Monrovia, Liberia

^22^ Center for Vaccine Development and Global Health, University of Maryland School of Medicine, 685 West Baltimore Street Baltimore, MD 21201-1509, USA

^23^ INSP (Institut Nationale de Santé Publique), Conakry, Guinea

^24^ École de santé publique de l’Université de Montréal, Montréal, Canada

^25^ National Institute of Mental Health, Bethesda, MD, USA

^26^ Univ. Bordeaux, INSERM, Institut Bergonié, CHU de Bordeaux, CIC-EC 1401, Euclid/F-CRIN clinical trials platform and U1219 BPH Inria Sistm, F-33000 Bordeaux, France

^27^ School of Statistics, University of Minnesota, Minneapolis, MN, USA

^28^ Integrated Research Facility at Fort Detrick (IRF-Frederick), National Institute of Allergy and Infectious Diseases, National Institutes of Health (NIH), Fort Detrick, Frederick, MD, USA

^29^ Univ. Bordeaux, INSERM, MART, UMS 54, F-33000 Bordeaux, France

^30^ Recherche Translationnelle Appliquée au VIH et aux Maladies Infectieuses, Institut de Recherche pour le Développement, University of Montpellier, INSERM, 34090, Montpellier, France.
